# Supplementary material for: Design and Activity of Novel Oxadiazole Based Compounds That Target Poly(ADP-ribose) Polymerase
Source: Molecules. 2022 Jan 21;27(3):703. doi: 10.3390/molecules27030703 (PMC8839658; doi:10.3390/molecules27030703)
Supplement: Supplementary file 1 [file molecules-27-00703-s001.zip › molecules-1436382-supplementary.pdf]

Design and activity of novel oxadiazole based compounds that target Poly (ADP-Ribose) Polymerase

Divakar Vishwanath, Swamy S. Girimanchanaika, Dukanya Dukanya, Shobith Rangappa, Jirui Yang, Vijay Pandey, Peter E. Lobie, Basappa Basappa

Supplementary data for newly synthesized molecules and their IC50 values determined against human breast cancer cells

Supporting information of ethyl 3-bromobenzoate (2a), and 3-bromobenzohydarzide (3a).

Ethyl 3-bromobenzoate (2a): white solid; 91% yield [1,2].

3-bromobenzohydarzide (3a): white solid; 82% yield; MP: 154-156 ^{o}C (lit.[2,3] 155-156 ^{o}C) [2,3].

1. Fan L, Zhang X, Sun Z, Zhang W, Ding Y, Fan W, Sun L, Zhao X, Lei H. Ancillary Ligands Dependent Structural Diversity of A Series of Metal−Organic Frameworks Based on 3,5-Bis(3-carboxyphenyl)pyridine. Cryst. Growth Des. 2013, 13, 2462−75.
2. Kerzare D, Chikhale R, Bansode R, Amnerkar N, Karodia N, Paradkar A, Khedekar P. Design, Synthesis, Pharmacological Evaluation and Molecular Docking Studies of Substituted Oxadiazolyl-2-Oxoindolinylidene Propane Hydrazide Derivatives. J. Braz. Chem. Soc. 2016, 27(11), 1998-2010.
3. Nisa M, Munawar MA, Iqbal A, Ahmed A, Ashraf M, Qurra-tul-Ann AG, Khan MA. Synthesis of novel 5-(aroylhydrazinocarbonyl)escitalopram as cholinesterase inhibitors. Eur. J. Med. Chem. 2017, 138, 396–406.

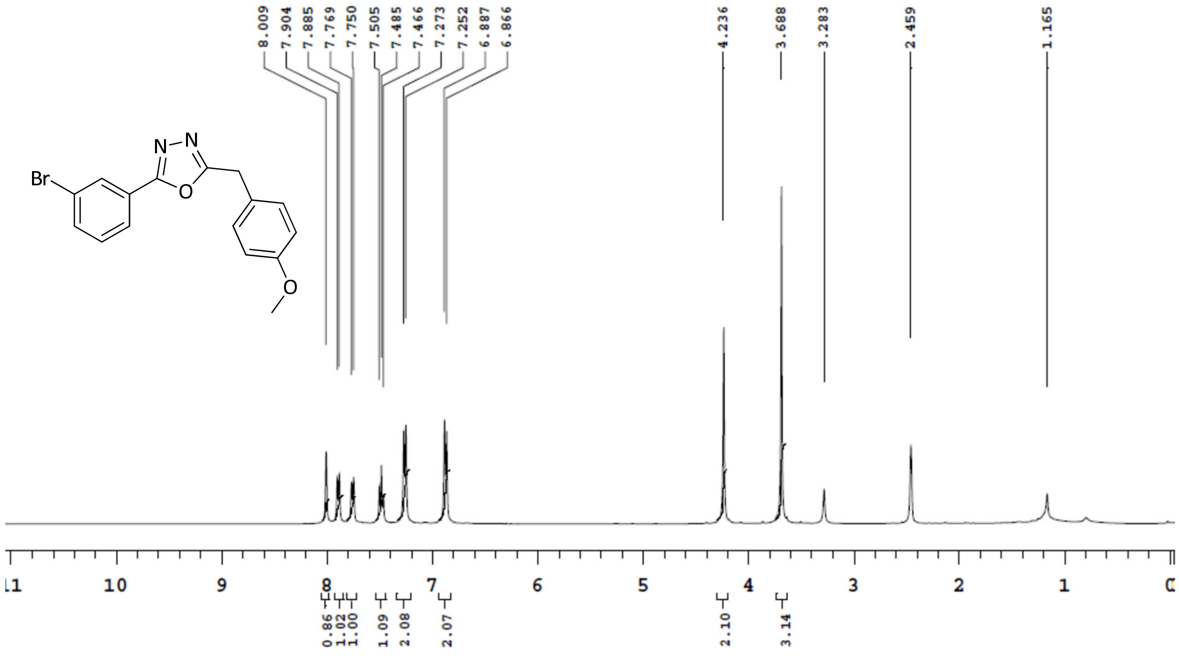

^{1}H NMR spectrum of 4b

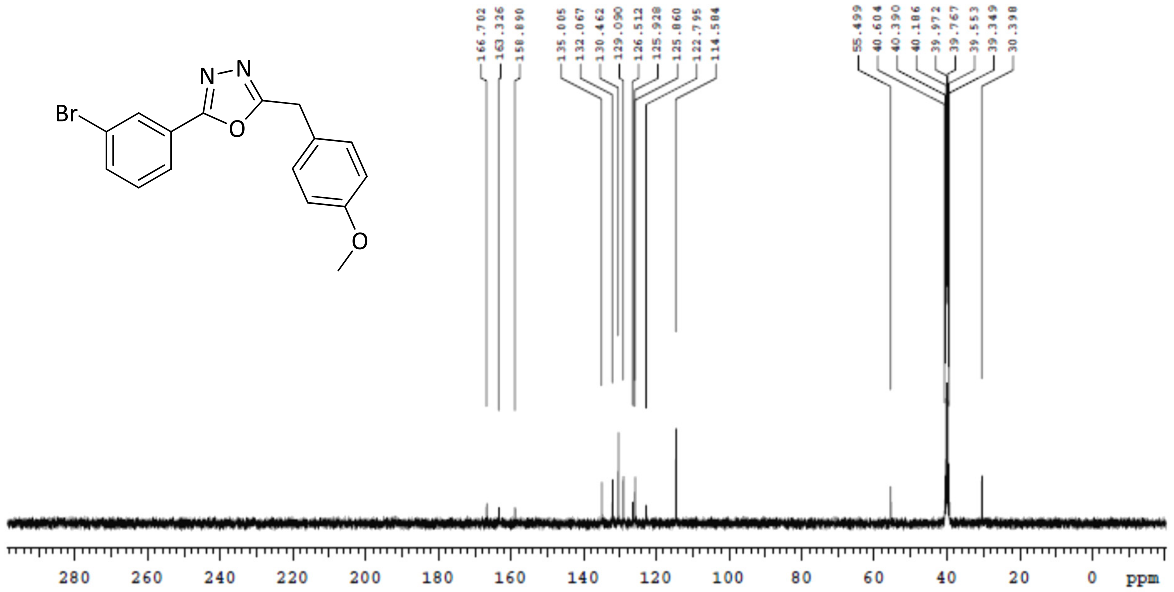

^{13}C NMR spectrum of 4b

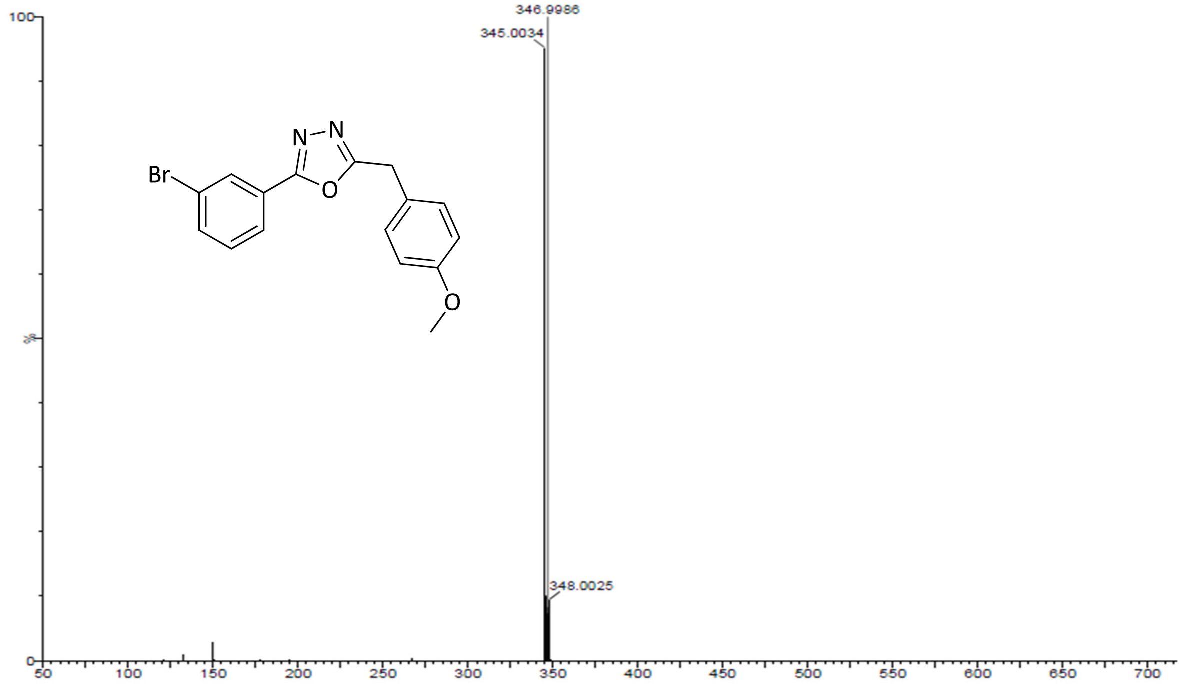

Mass spectrum of 4b

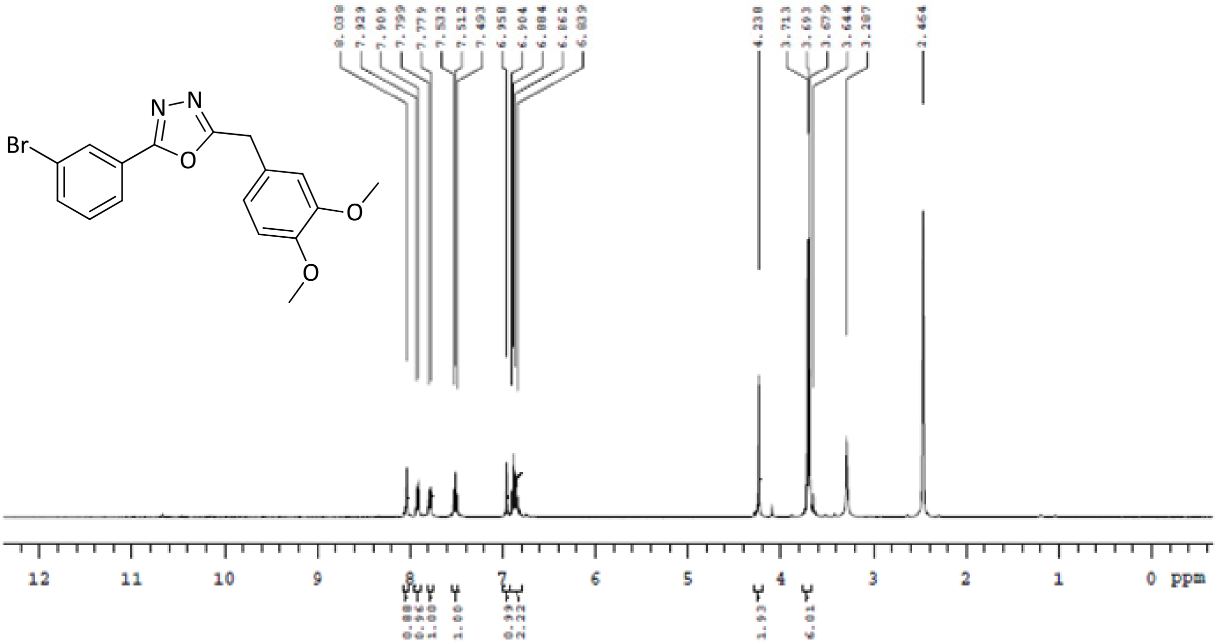

^{1}H NMR spectrum of 4c

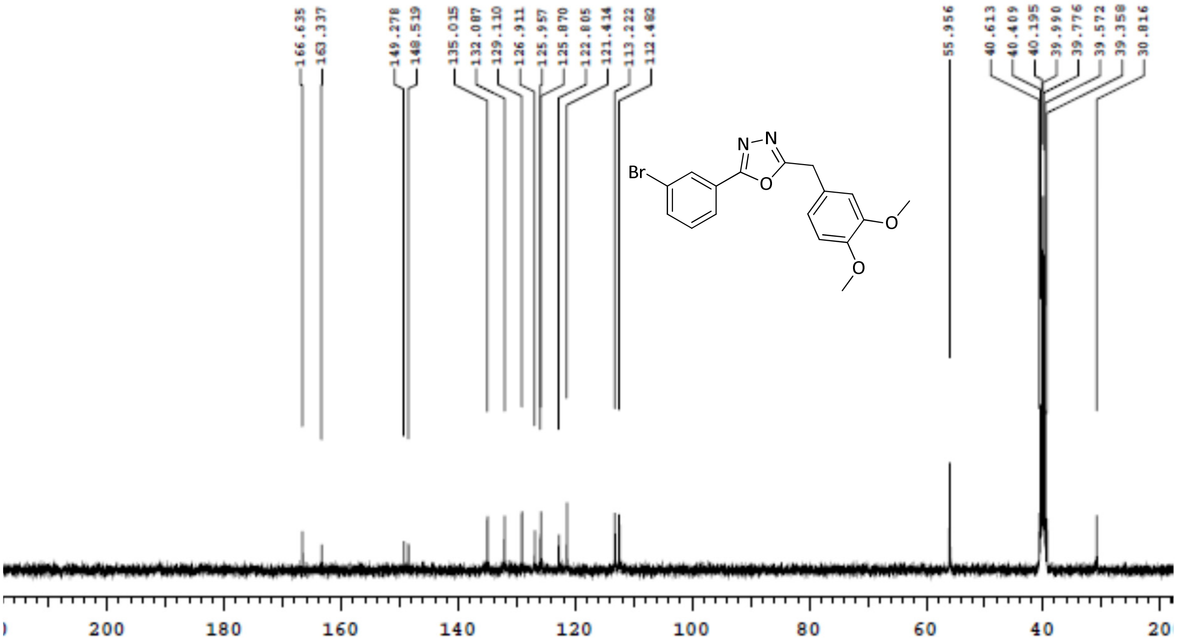

^{13}C NMR spectrum of 4c

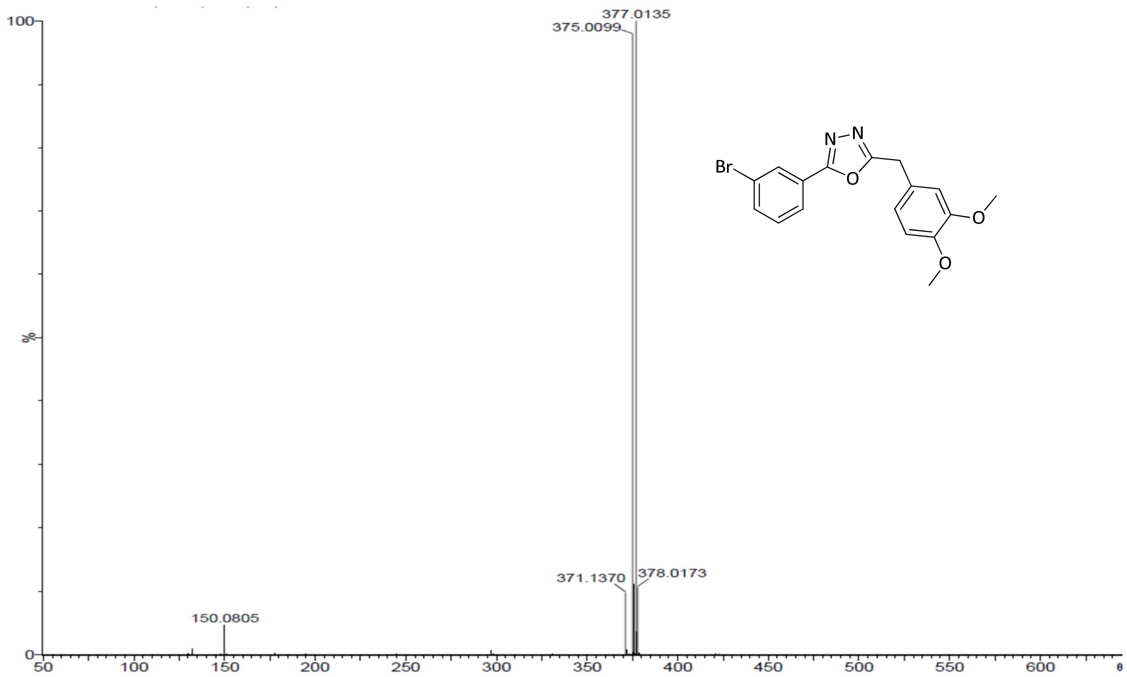

Mass spectrum of 4c

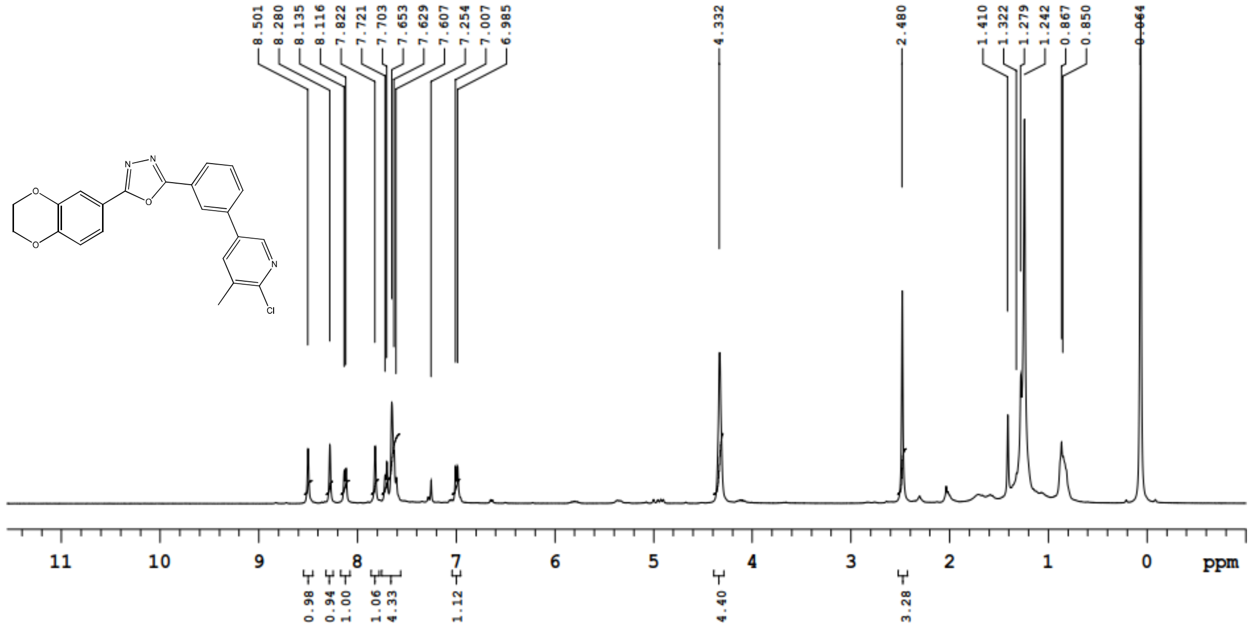

^{1}H NMR spectrum of 5a

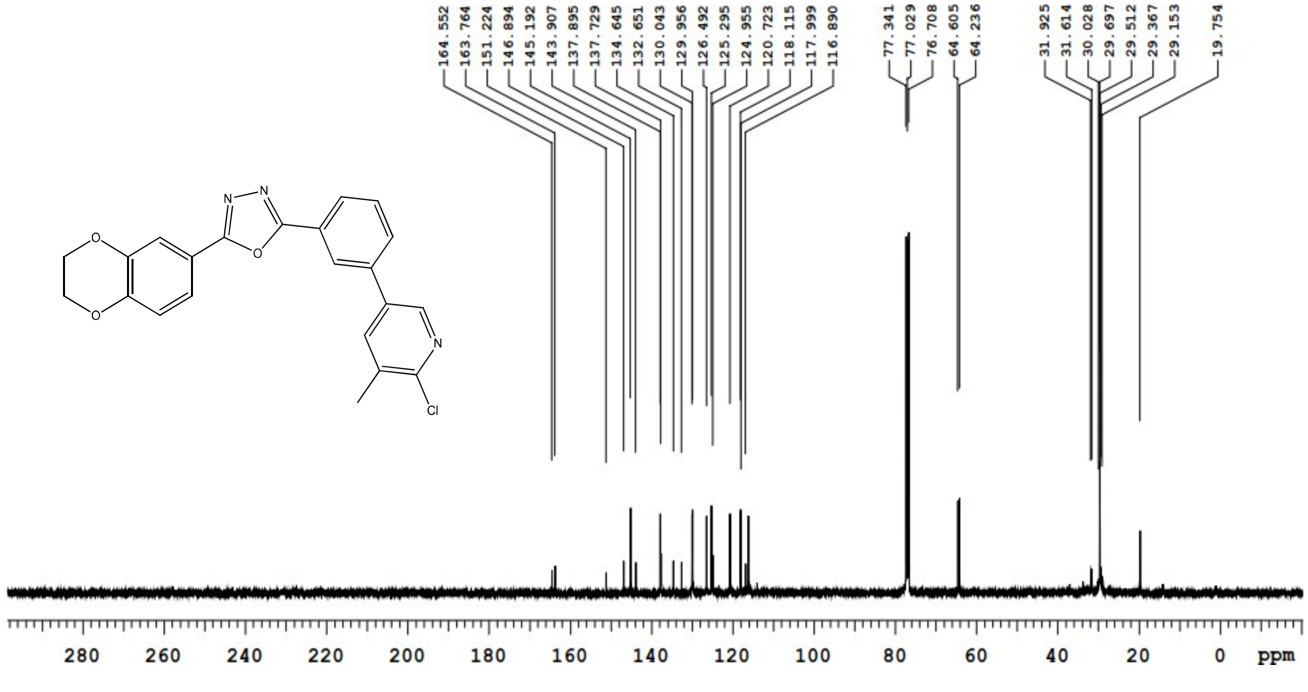

^{13}C NMR spectrum of 5a

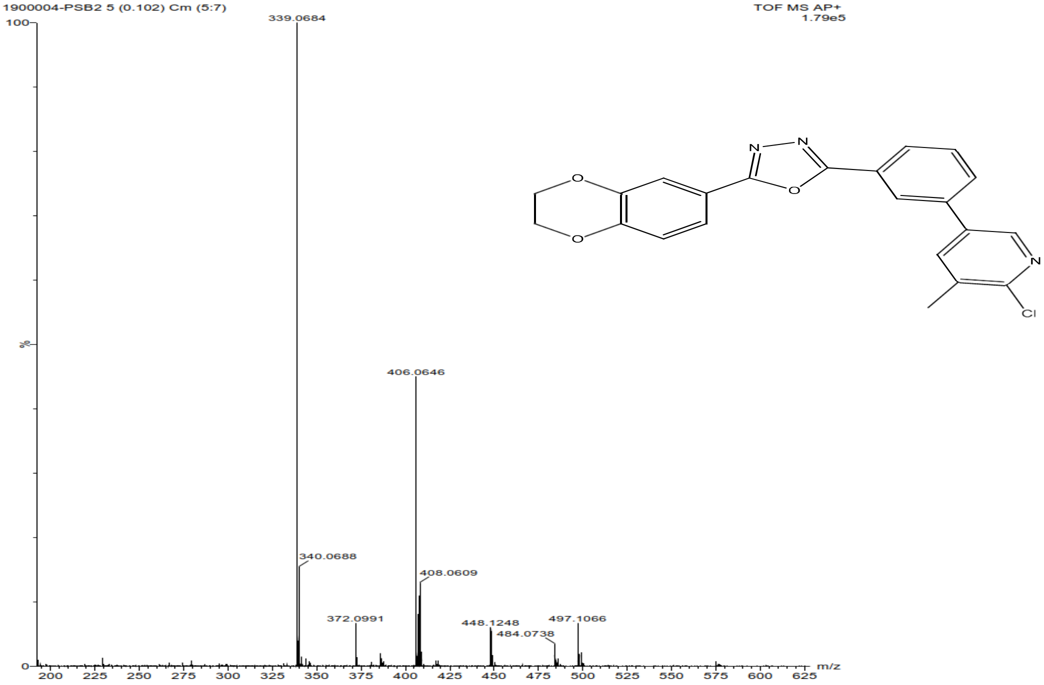

Mass spectrum of 5a

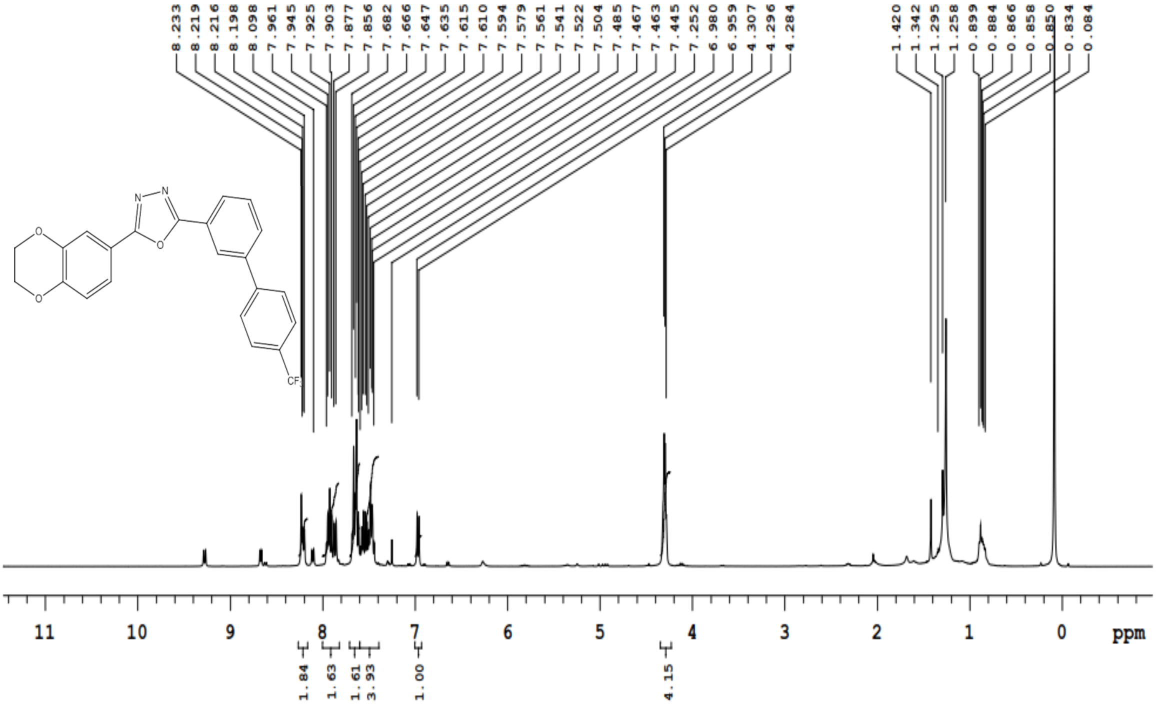

^{1}H NMR spectrum of 5b

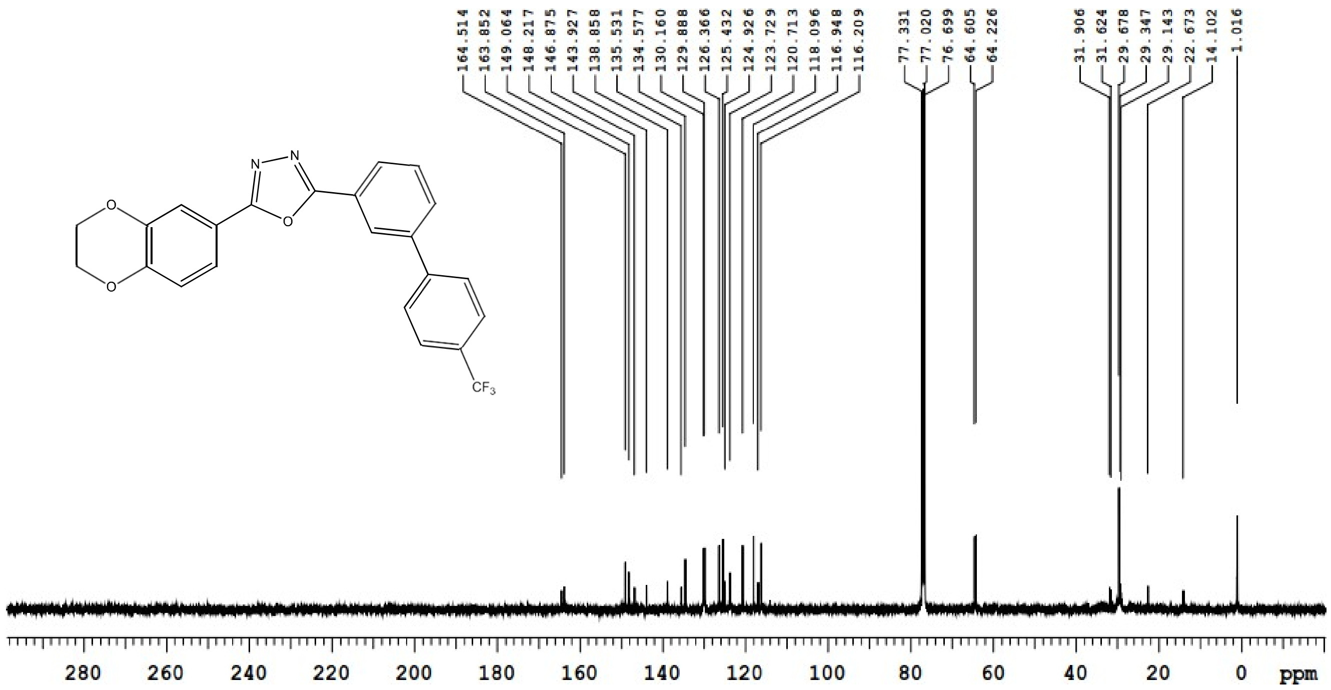

^{13}C NMR spectrum of 5b

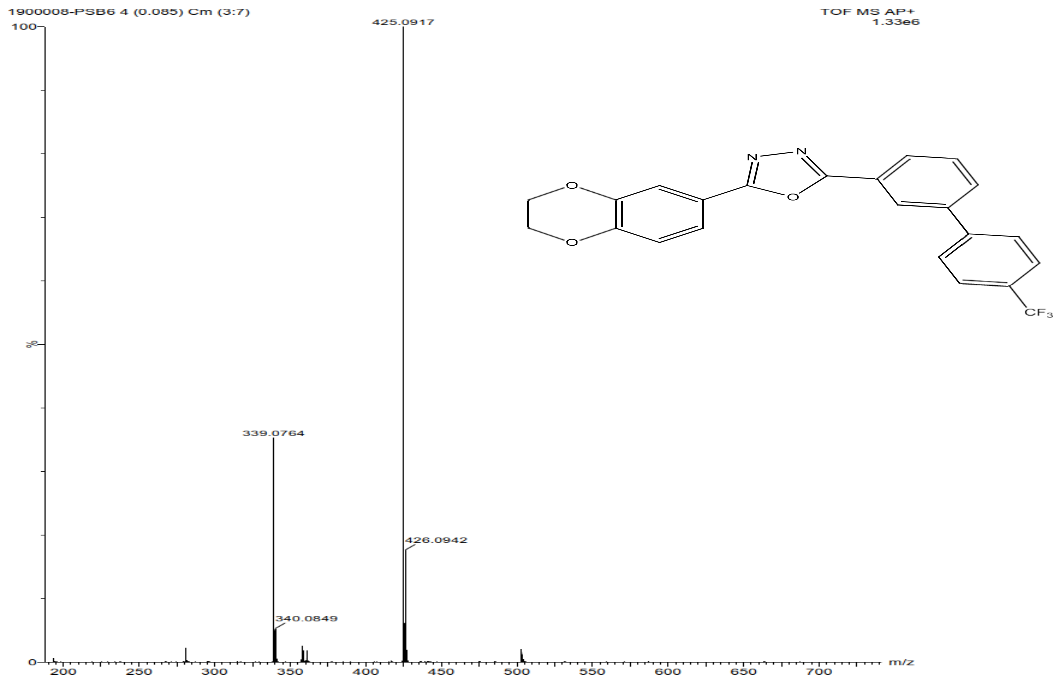

Mass spectrum of 5b

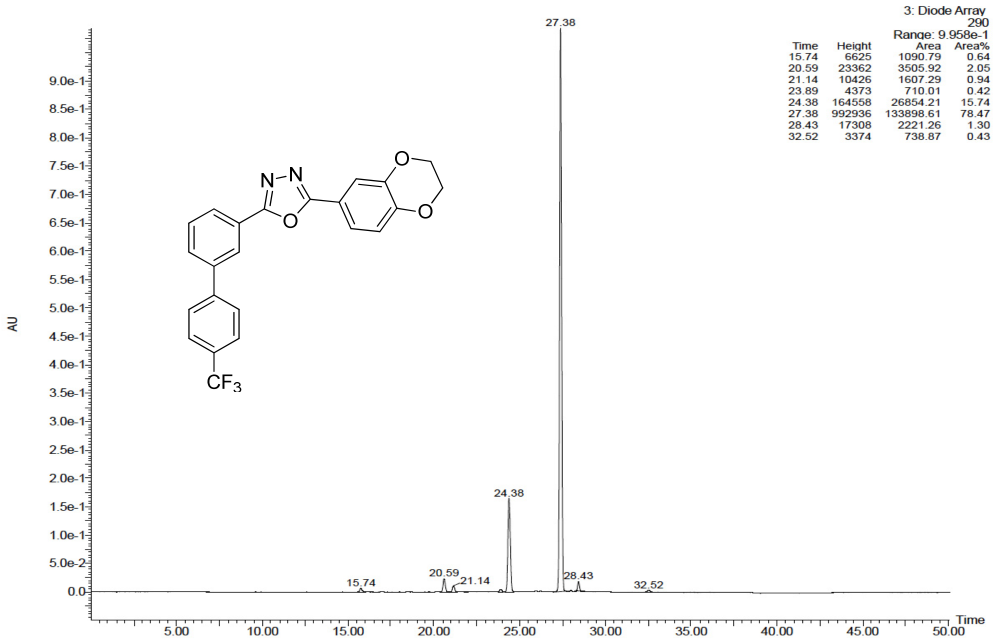

HPLC of 5b

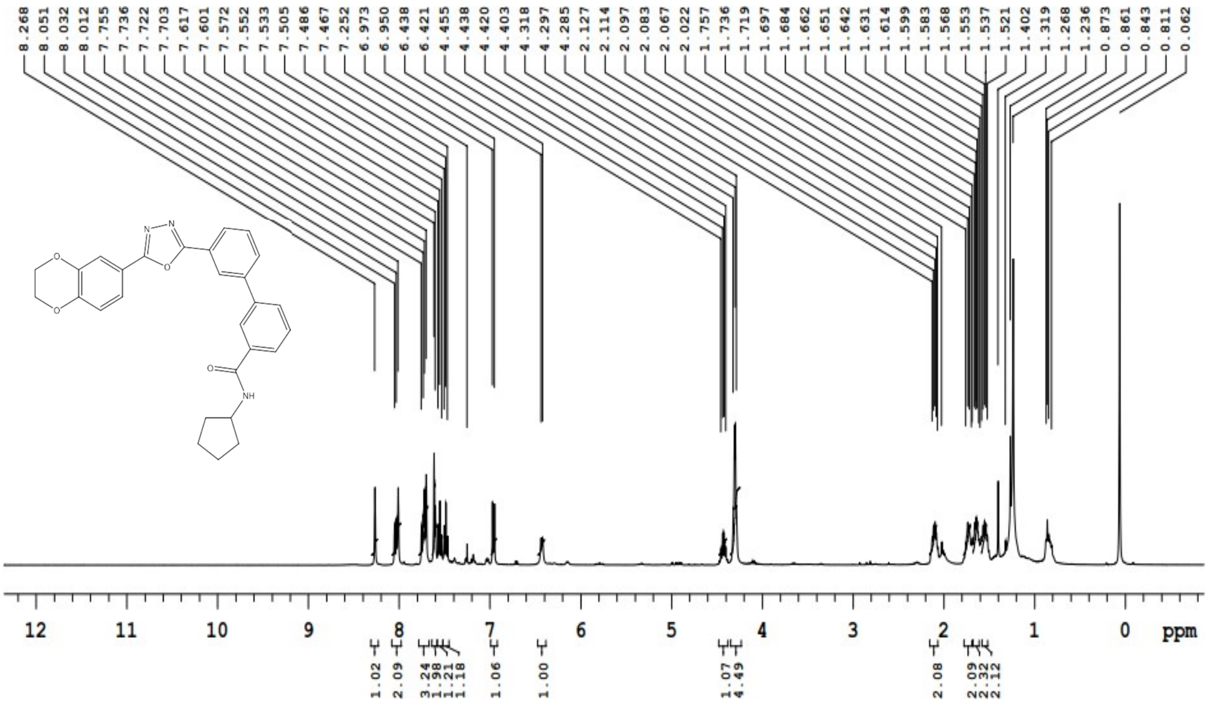

¹H NMR spectrum of 5c

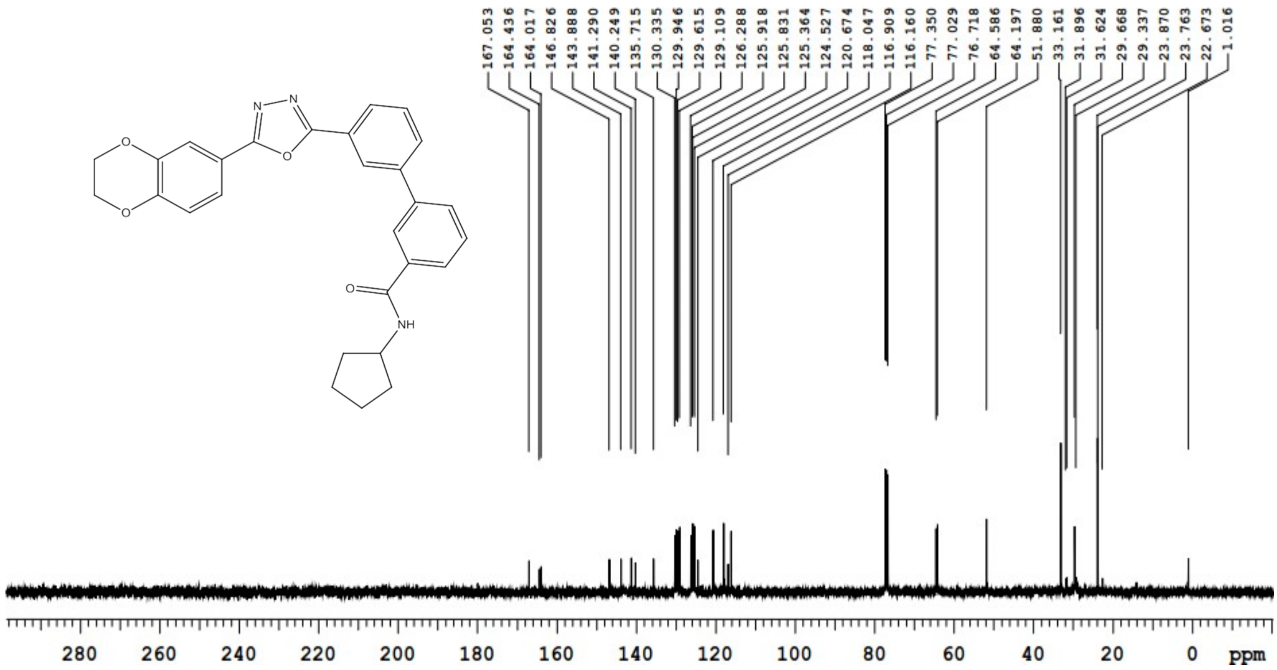

^{13}C NMR spectrum of 5c

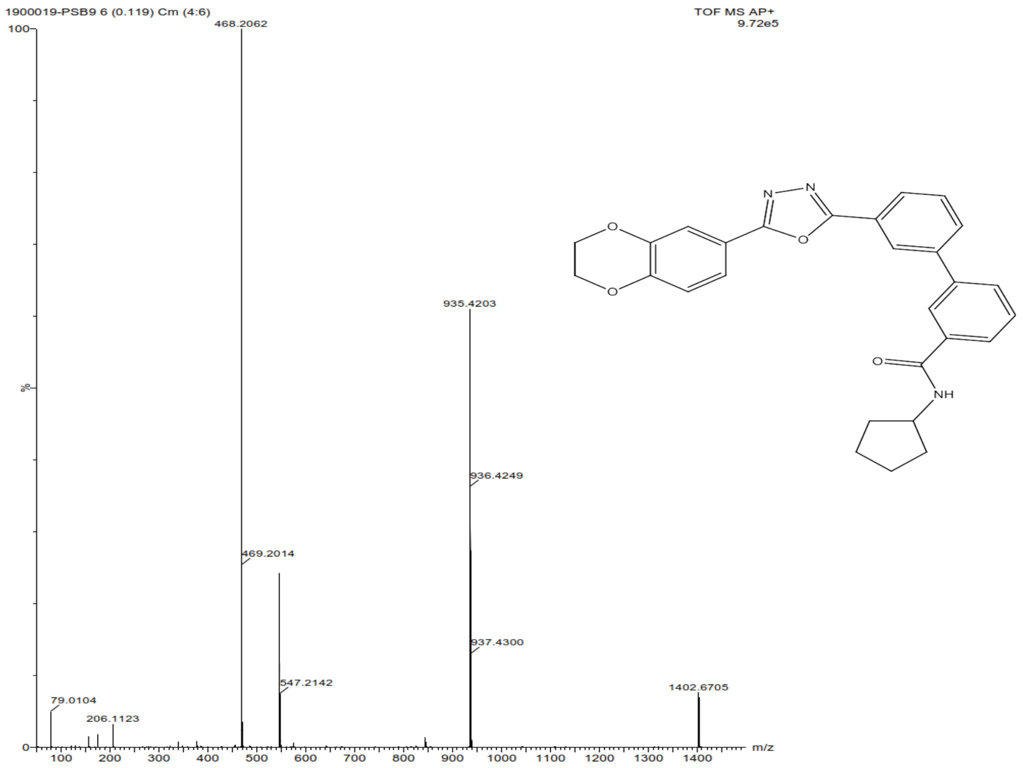

Mass spectrum of 5c

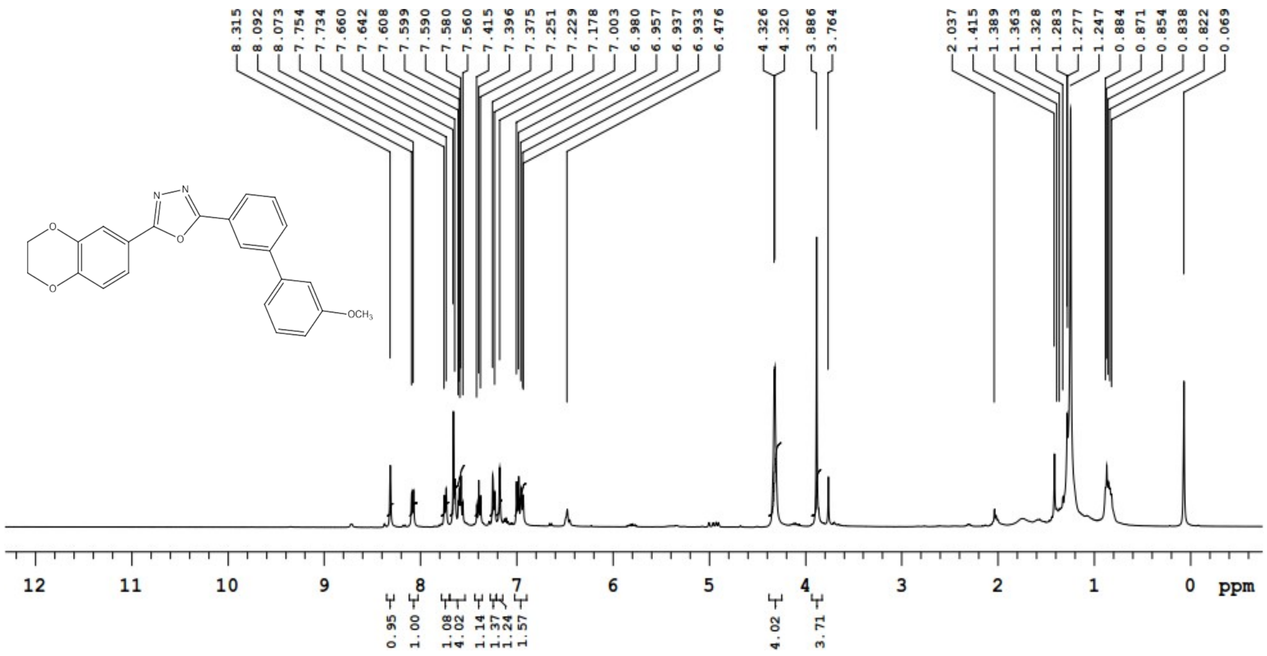

^{1}H NMR spectrum of 5d

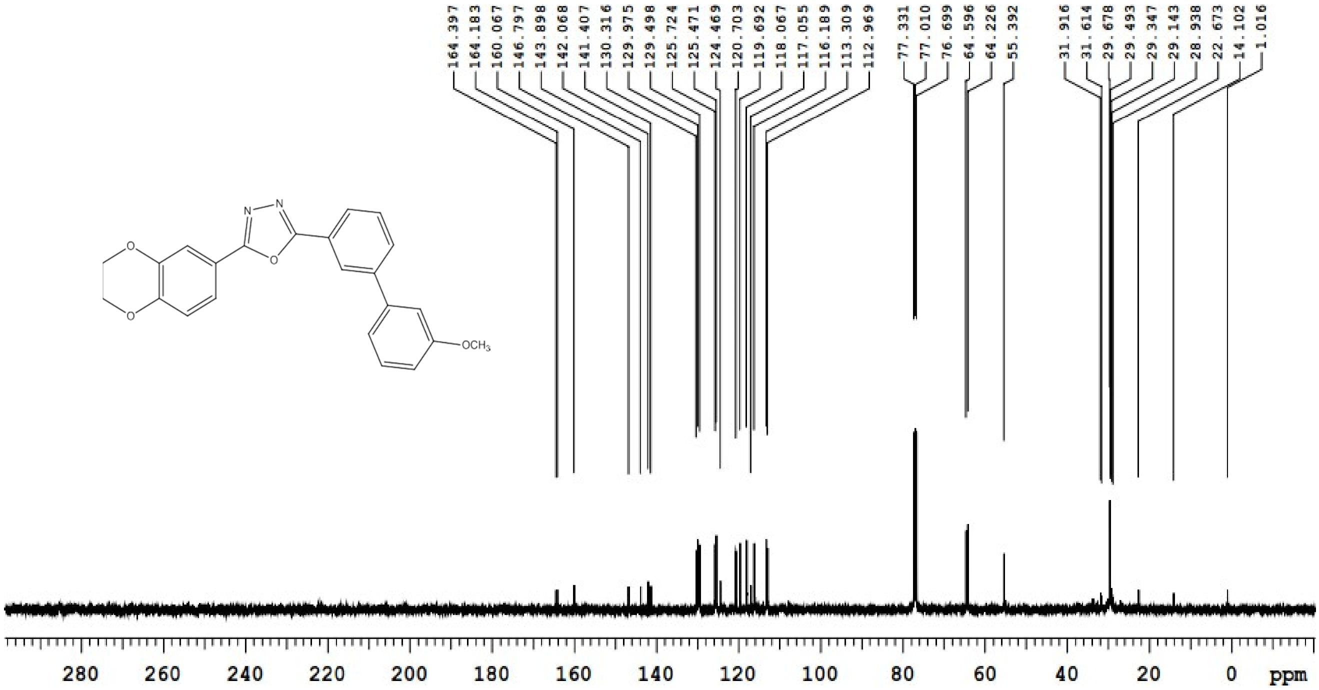

^{13}C NMR spectrum of 5d

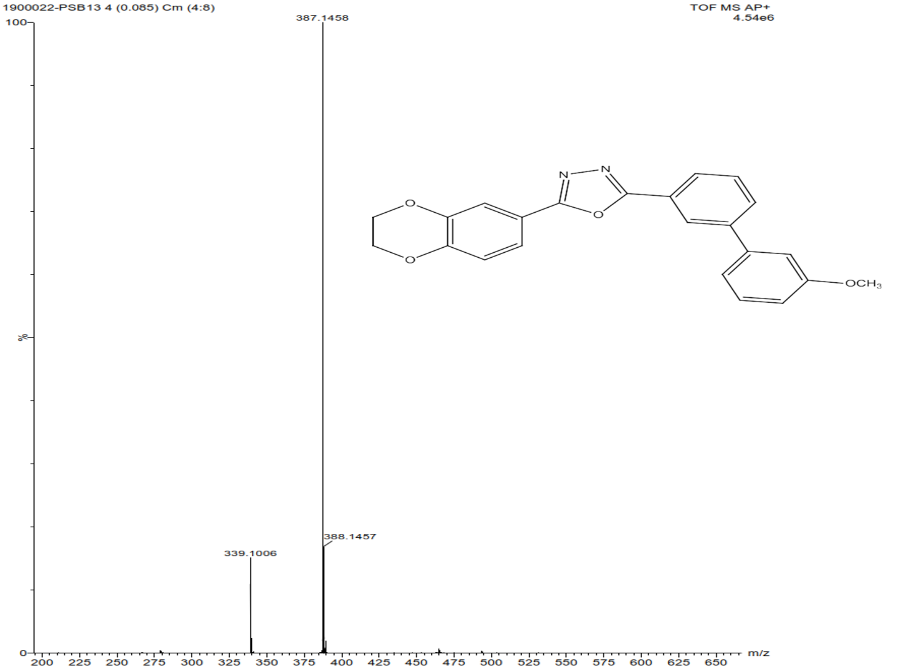

Mass spectrum of 5d

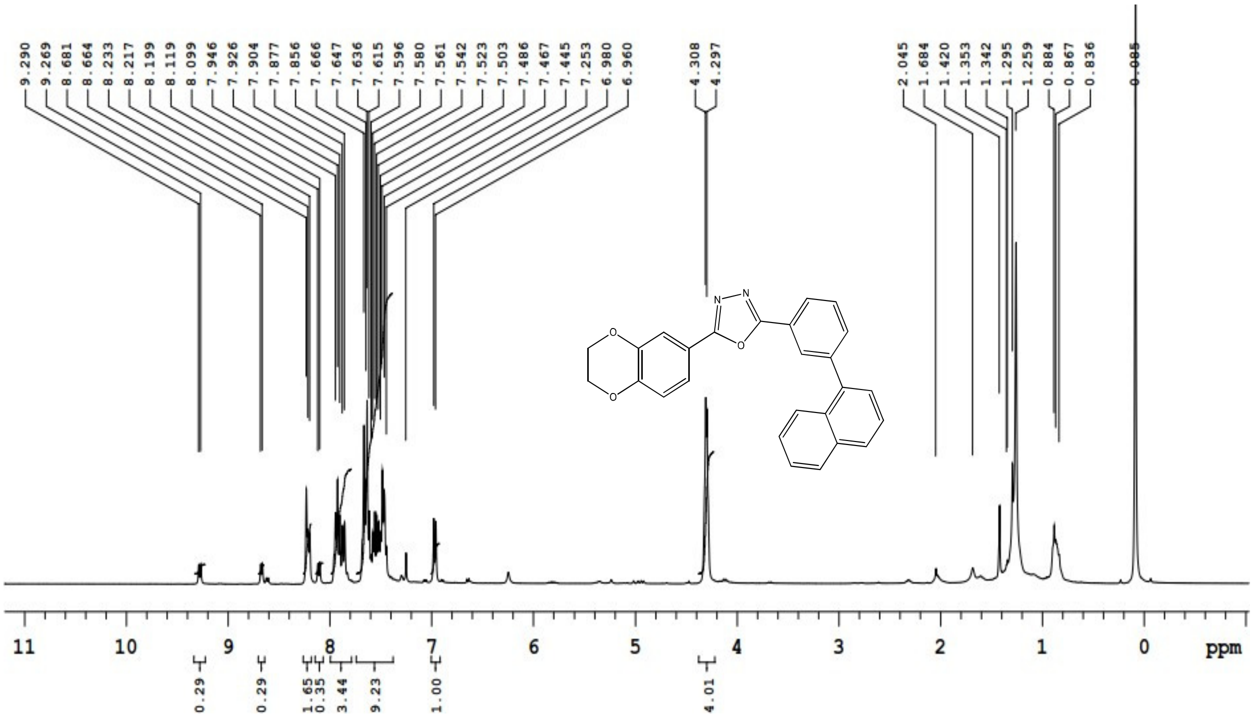

^{1}H NMR spectrum of 5e

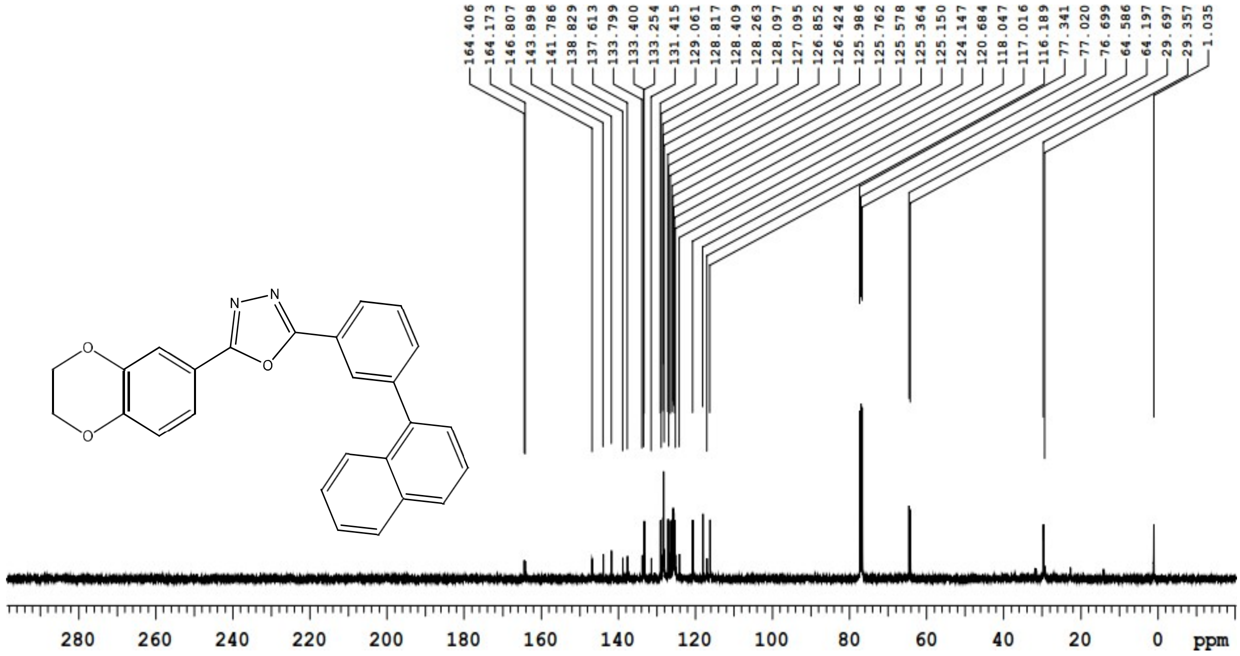

^{13}C NMR spectrum of 5e

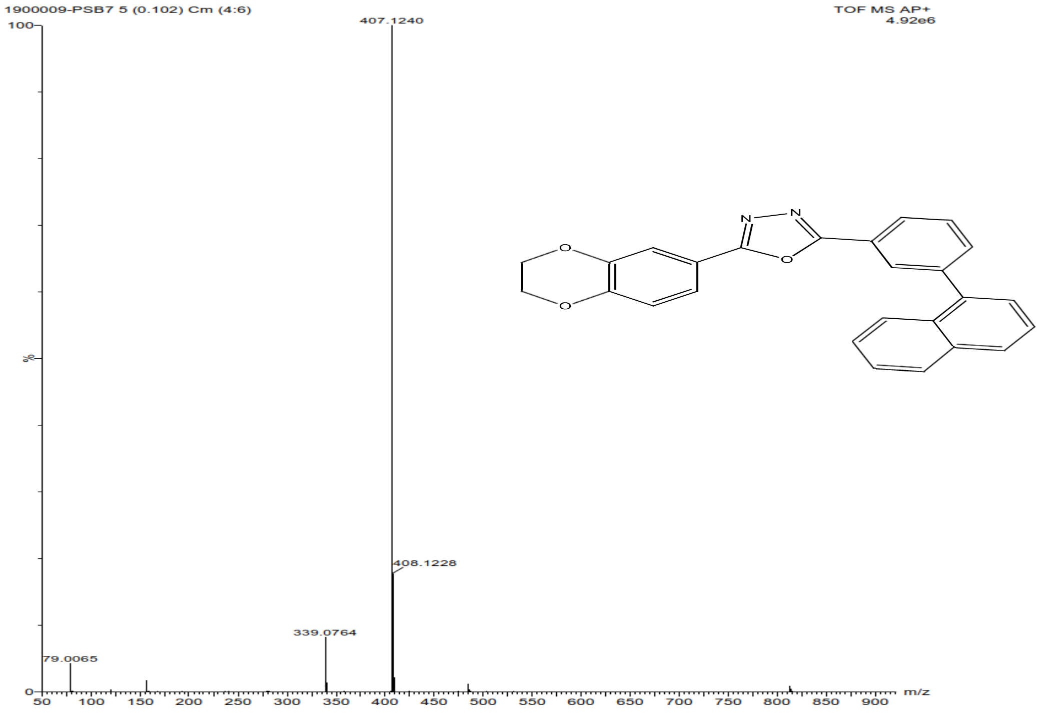

Mass spectrum of 5e

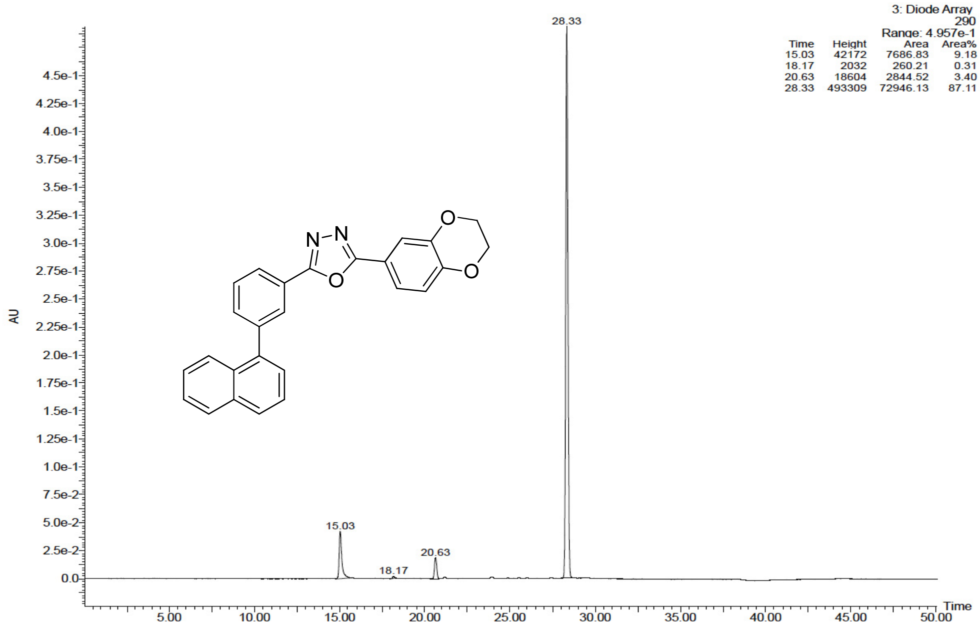

HPLC of 5e

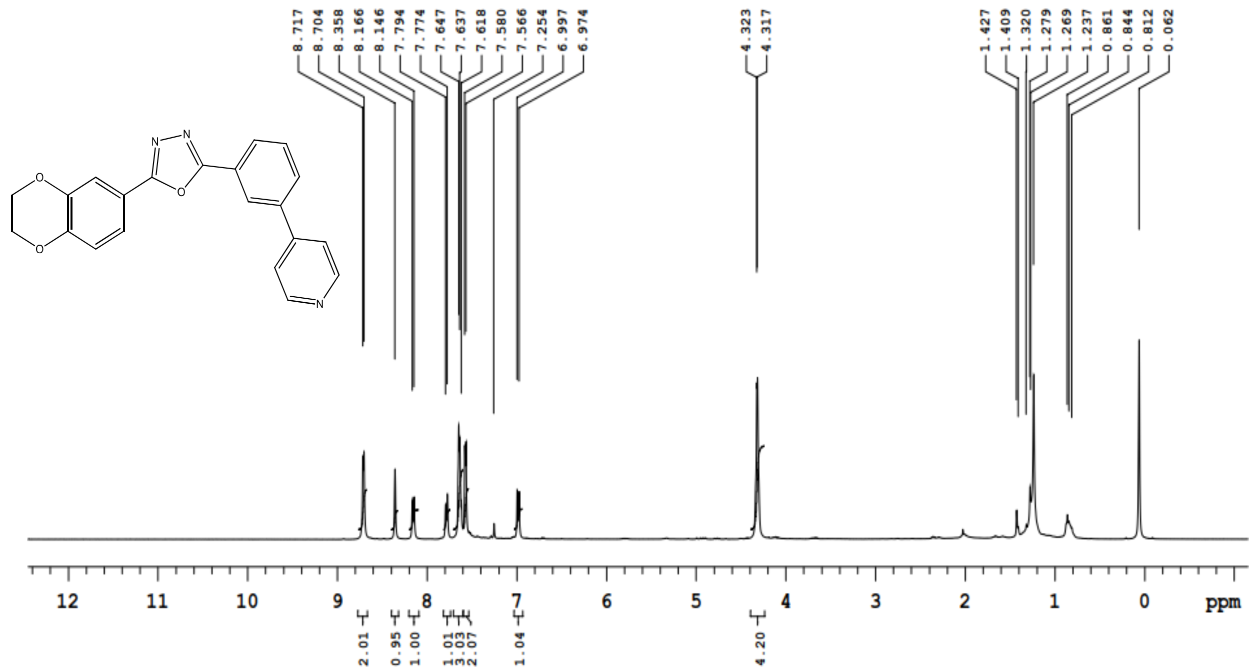

^{1}H NMR spectrum of 5f

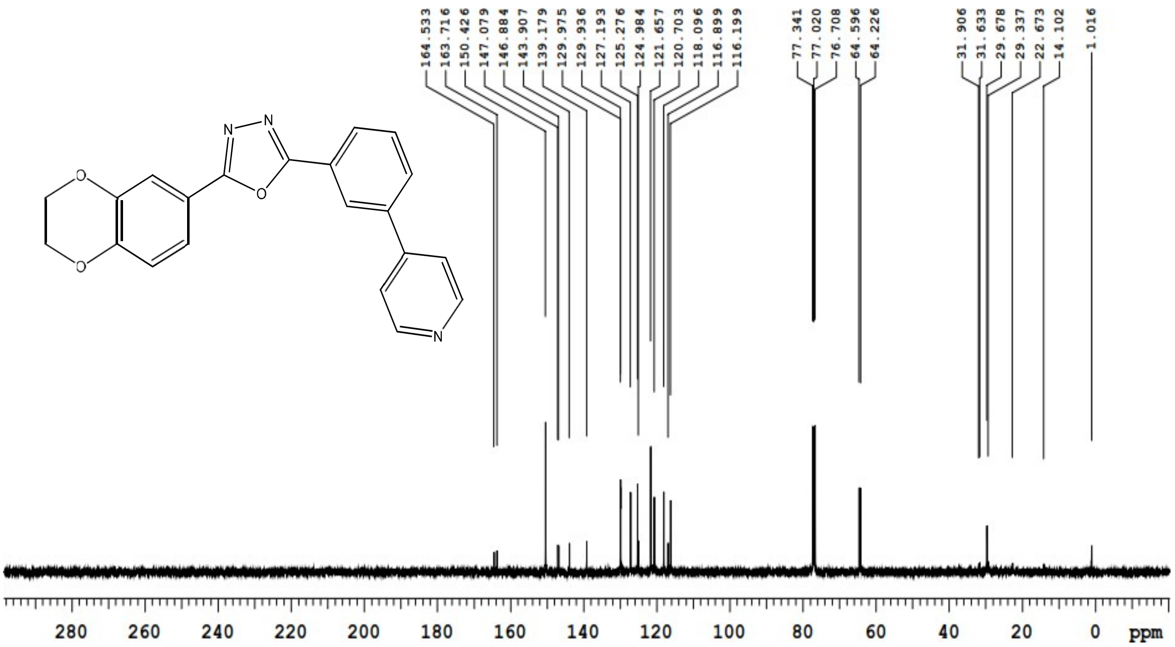

^{13}C NMR spectrum of 5f

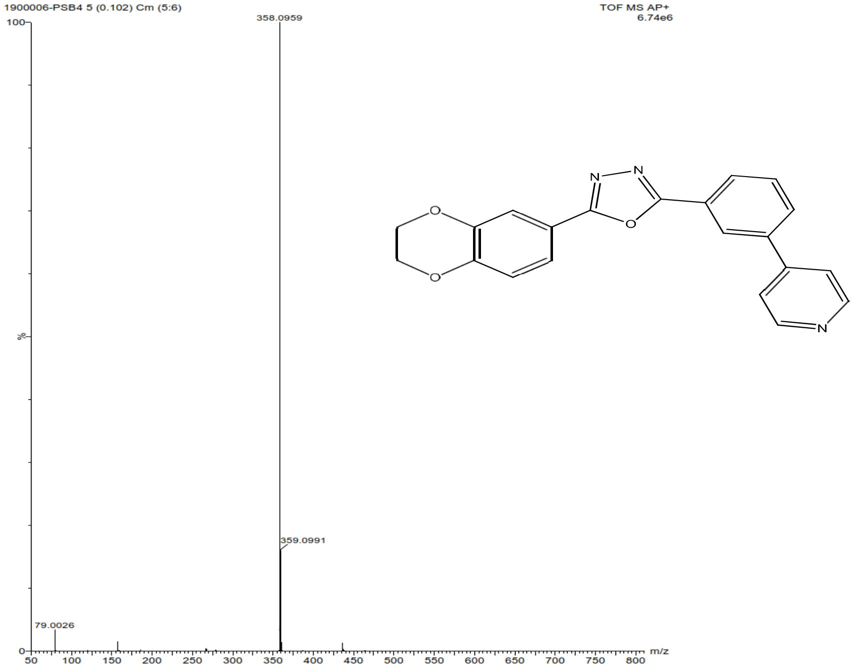

Mass spectrum of 5f

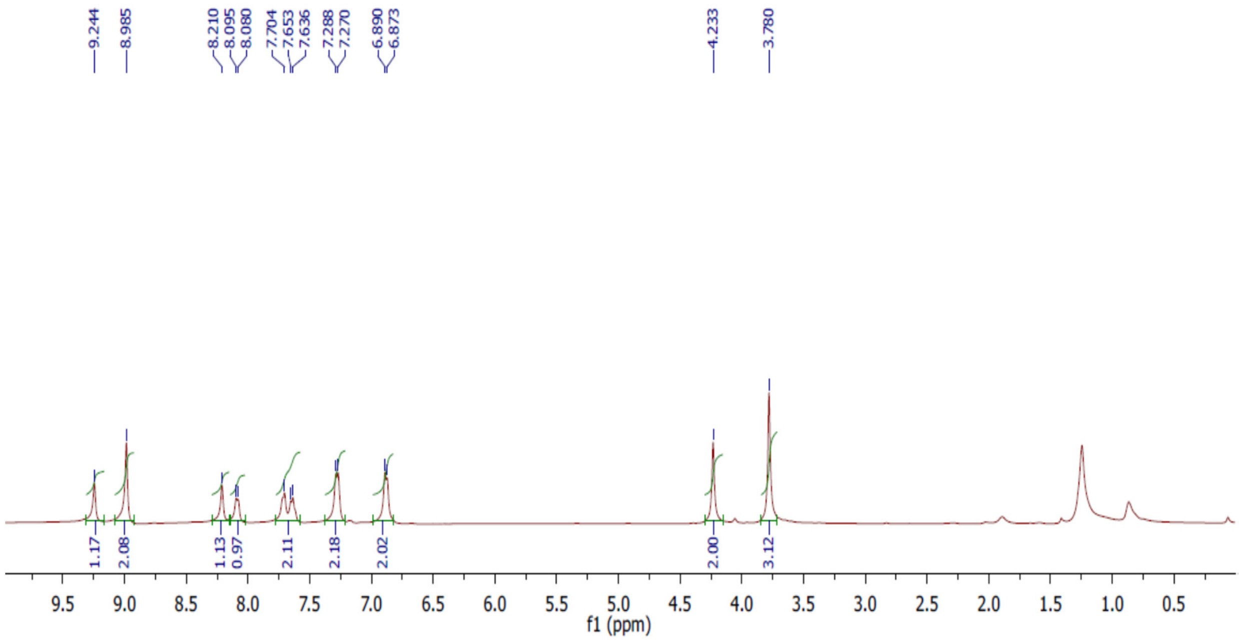

^{1}H NMR spectrum of 5g

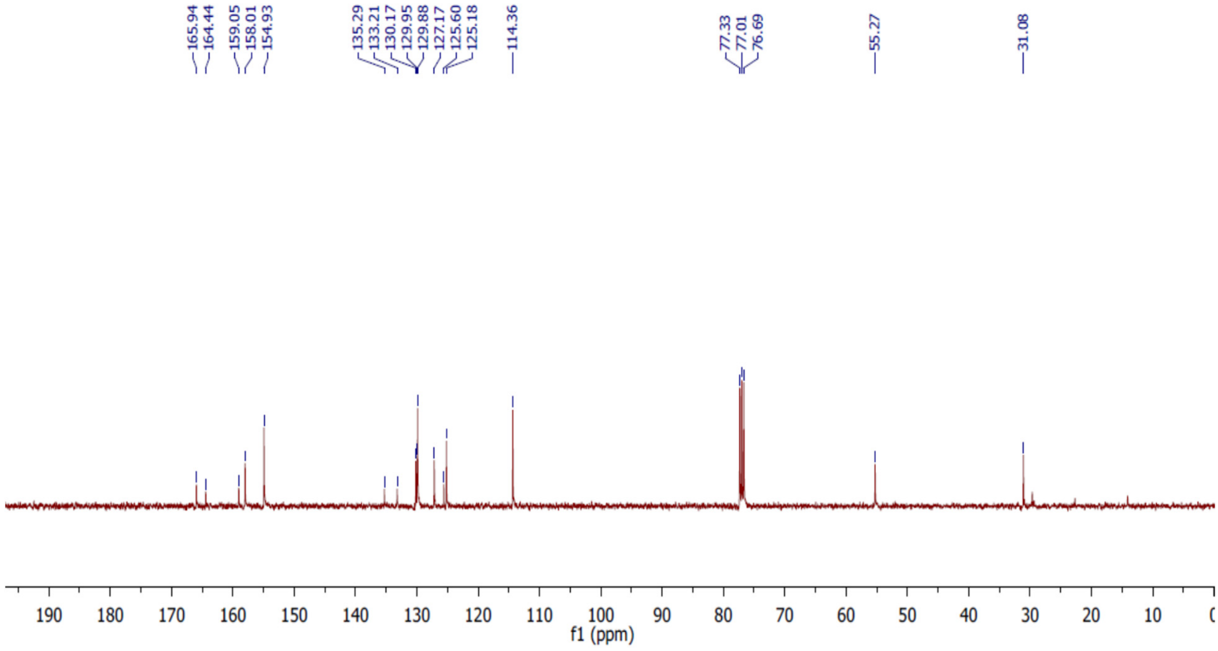

^{13}C NMR spectrum of 5g

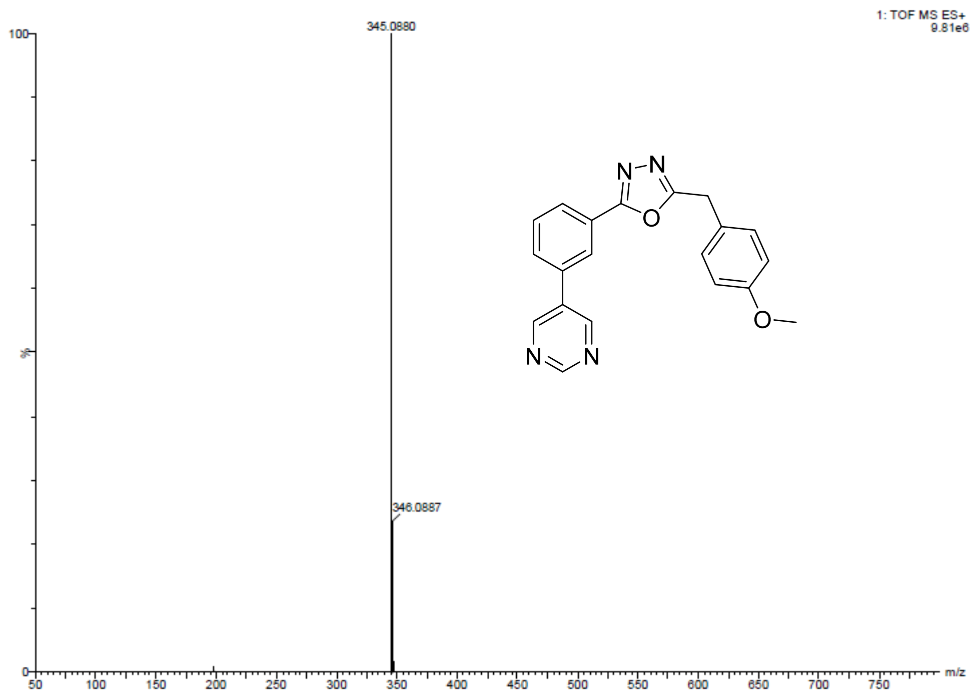

Mass spectrum of 5g

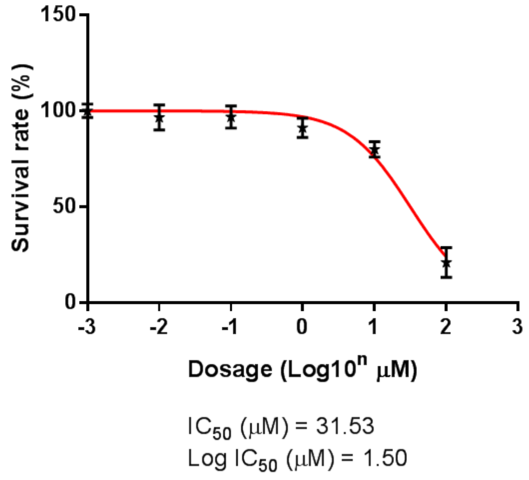

- **Cell line: MCF7 (2000 cells/per well[96])**
- **Treated time: 72hrs**
- **Assay: MTT (90mins incubated)**
- **Data: 5g**

Conc. (μM)	Viability	
	AVE.	± SD.
0	100.00	3.58
0.01	96.55	6.55
0.1	96.81	5.70
1	91.16	5.05
10	79.95	4.05
100	20.93	7.71

Log curve for the compound 5g

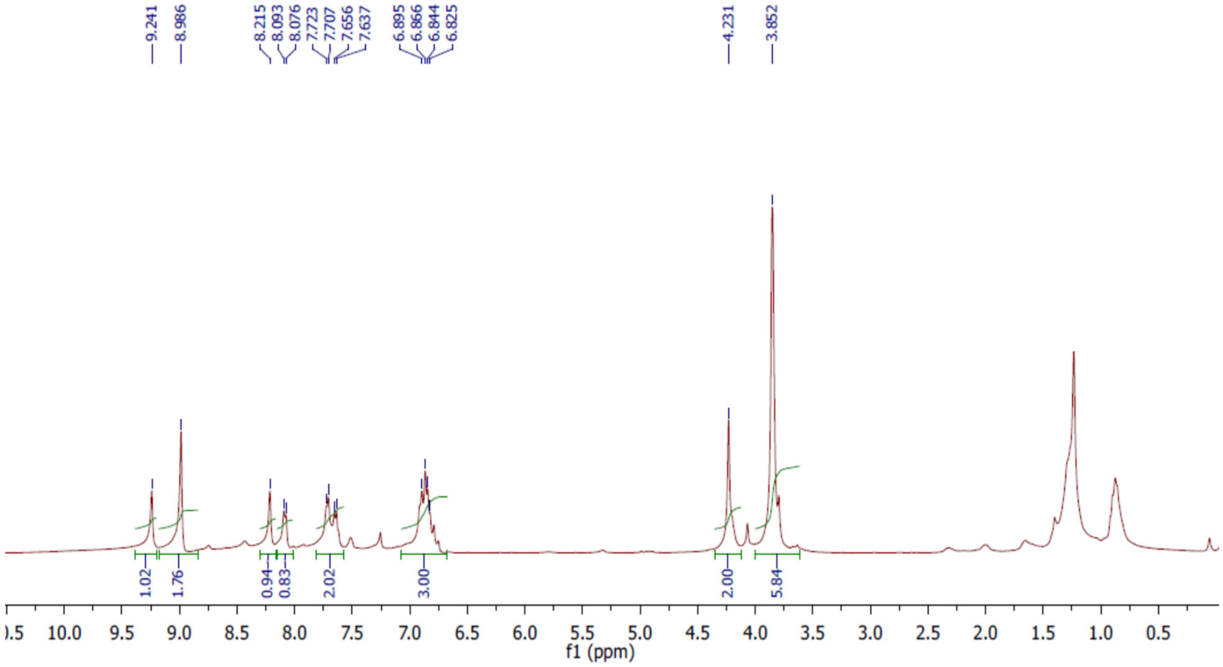

^{1}H NMR spectrum of 5h

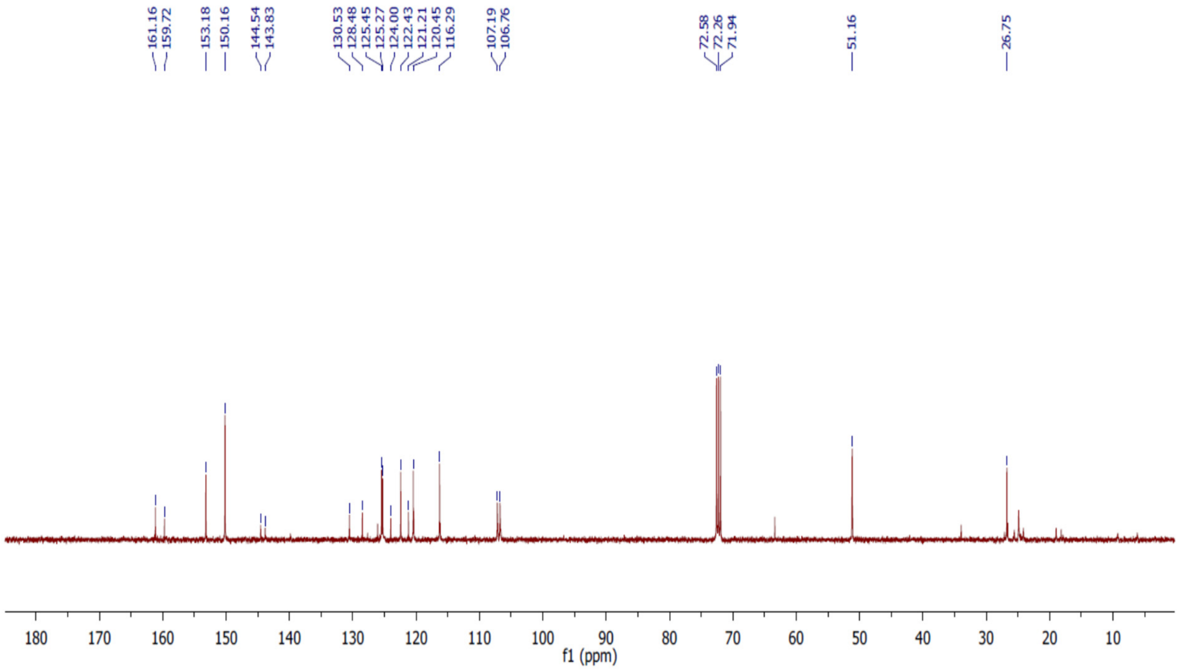

^{13}C NMR spectrum of 5h

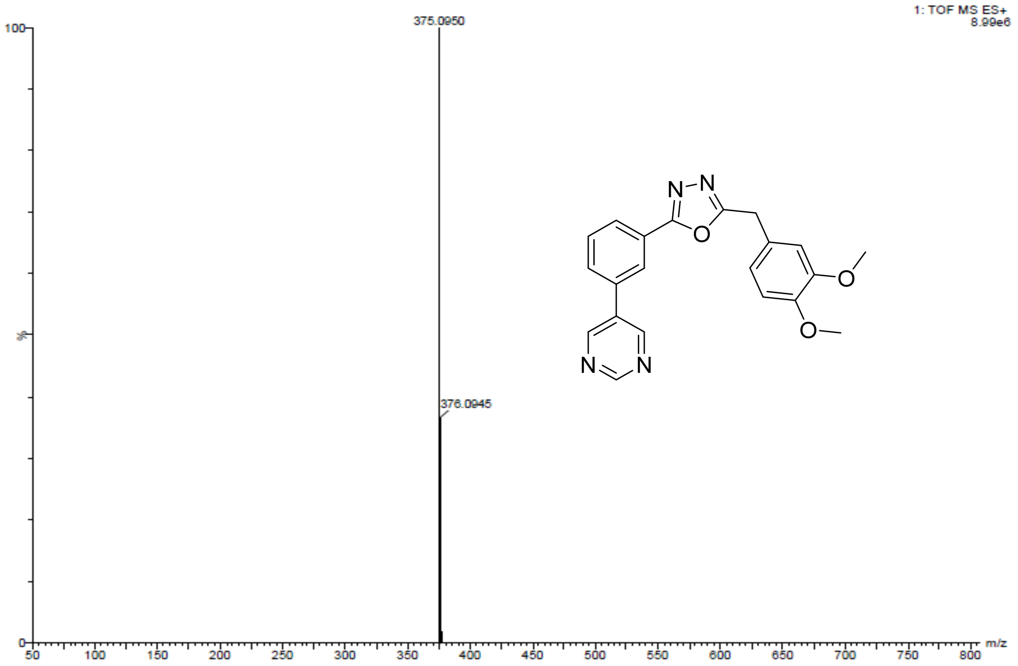

Mass spectrum of 5h

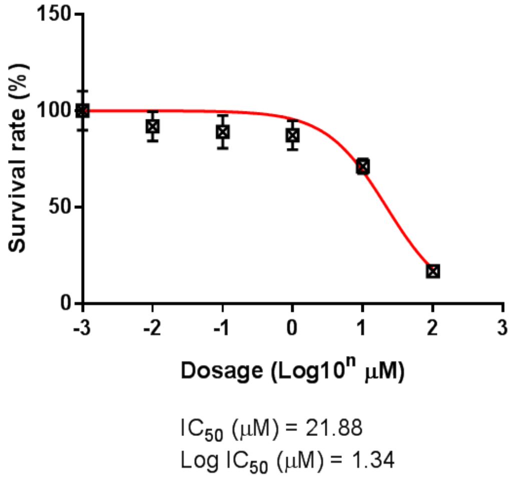

- Cell line: MCF7 (2000 cells/per well[96])
- Treated time: 72hrs
- Assay: MTT (90mins incubated)
- Data: 5h

Conc. (μM)	Viability	
	AVE.	± SD.
**0**	100.00	10.16
**0.01**	91.92	7.63
**0.1**	89.07	8.50
**1**	87.37	7.57
**10**	71.25	3.83
**100**	16.82	0.48

Log curve for the compound 5h

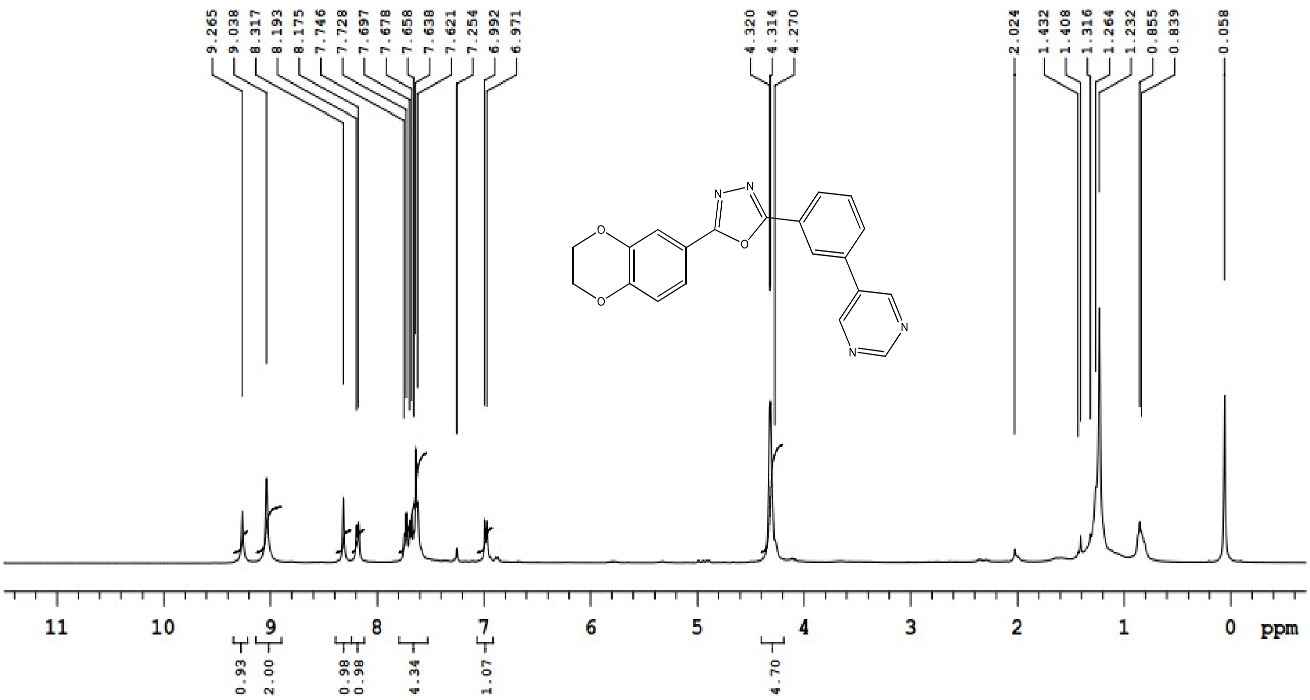

^{1}H NMR spectrum of 5i

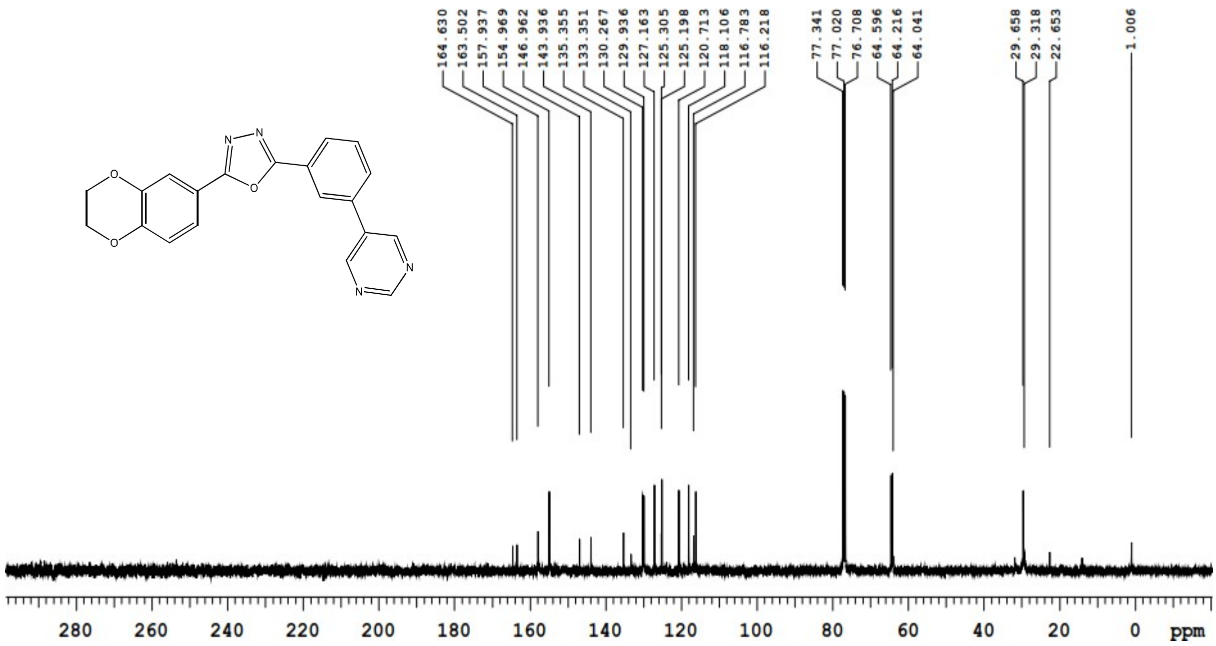

^{13}C NMR spectrum of 5i

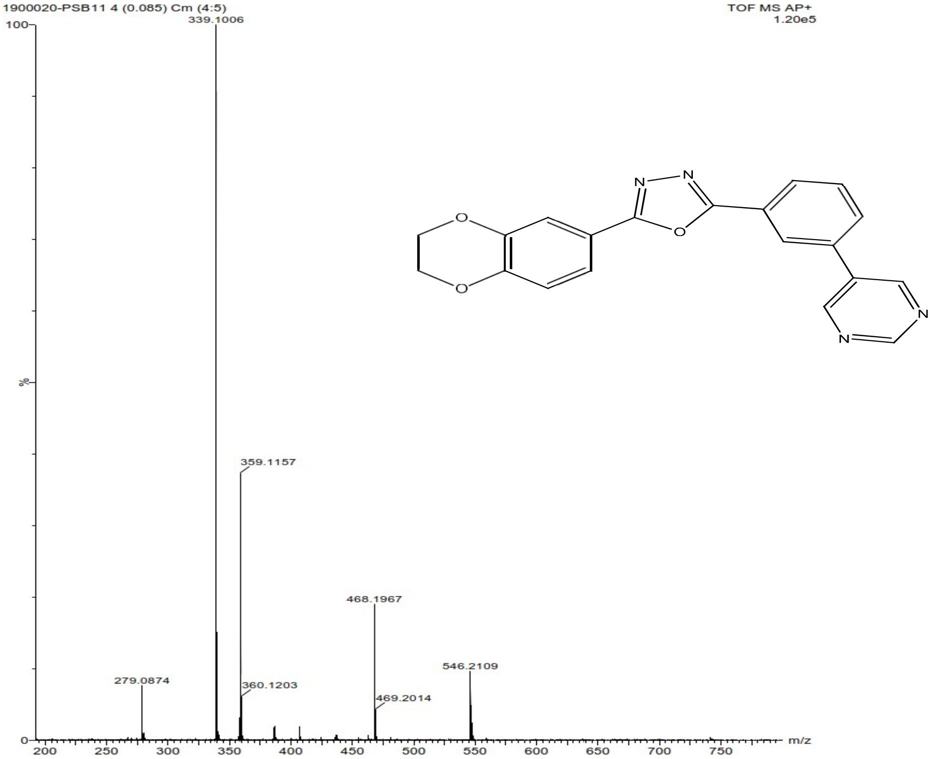

Mass spectrum of 5i

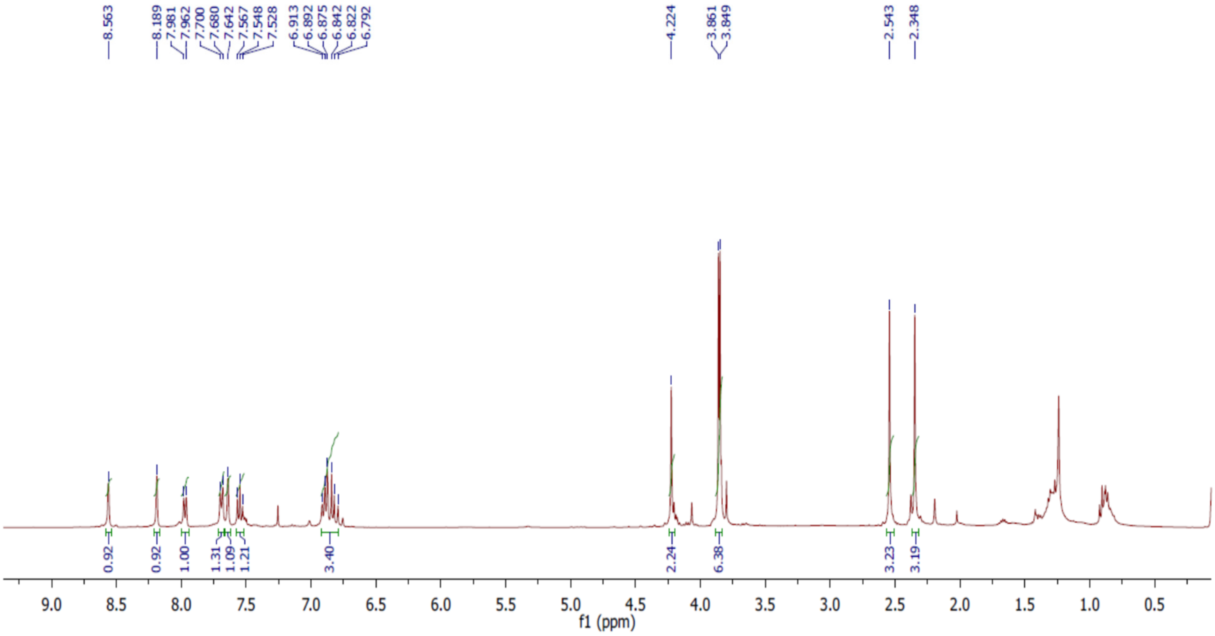

^{1}H NMR spectrum of 5j

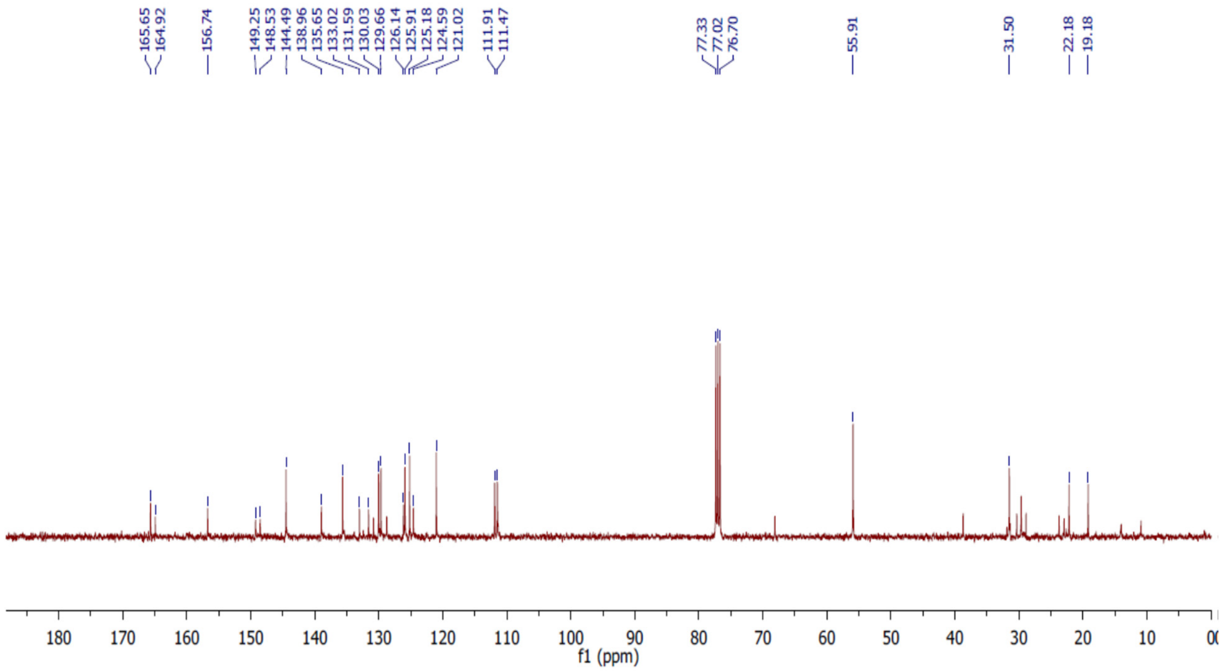

^{13}C NMR spectrum of 5j

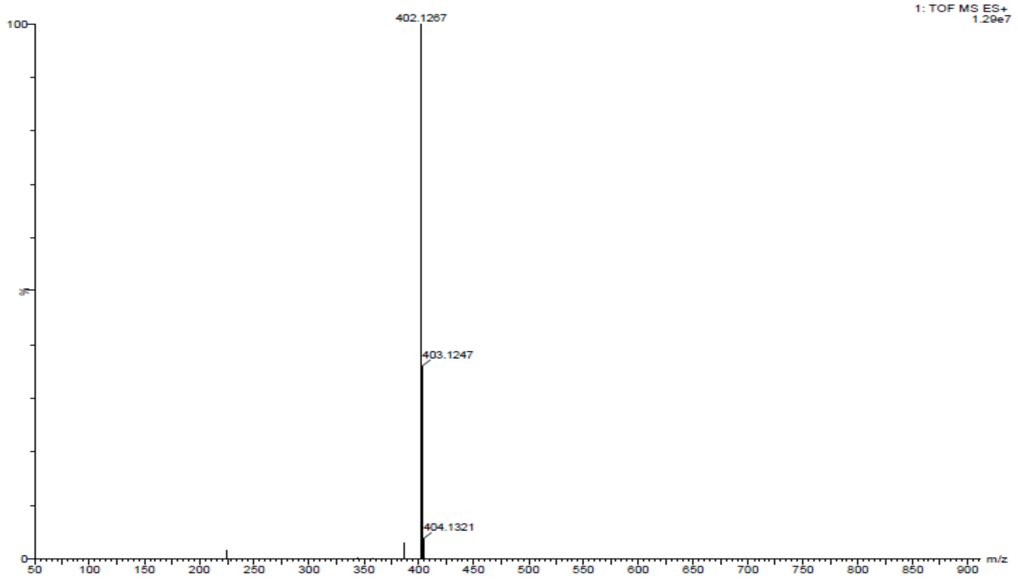

Mass spectrum of 5j

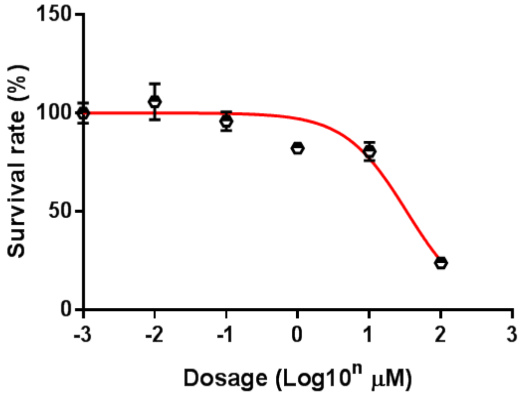

IC_{50} (μM) = 33.22
Log IC_{50} (μM) = 1.52

- **Cell line: MCF7 (2000 cells/per well[96])**
- **Treated time: 72hrs**
- **Assay: MTT (90mins incubated)**
- **Data: 5j**

**Conc. (μM)**	**Viability**	
	**AVE.**	**± SD.**
**0**	100.00	5.17
**0.01**	105.63	9.17
**0.1**	95.87	4.86
**1**	82.10	2.86
**10**	80.47	4.66
**100**	23.84	2.67

Log curve for the compound 5j

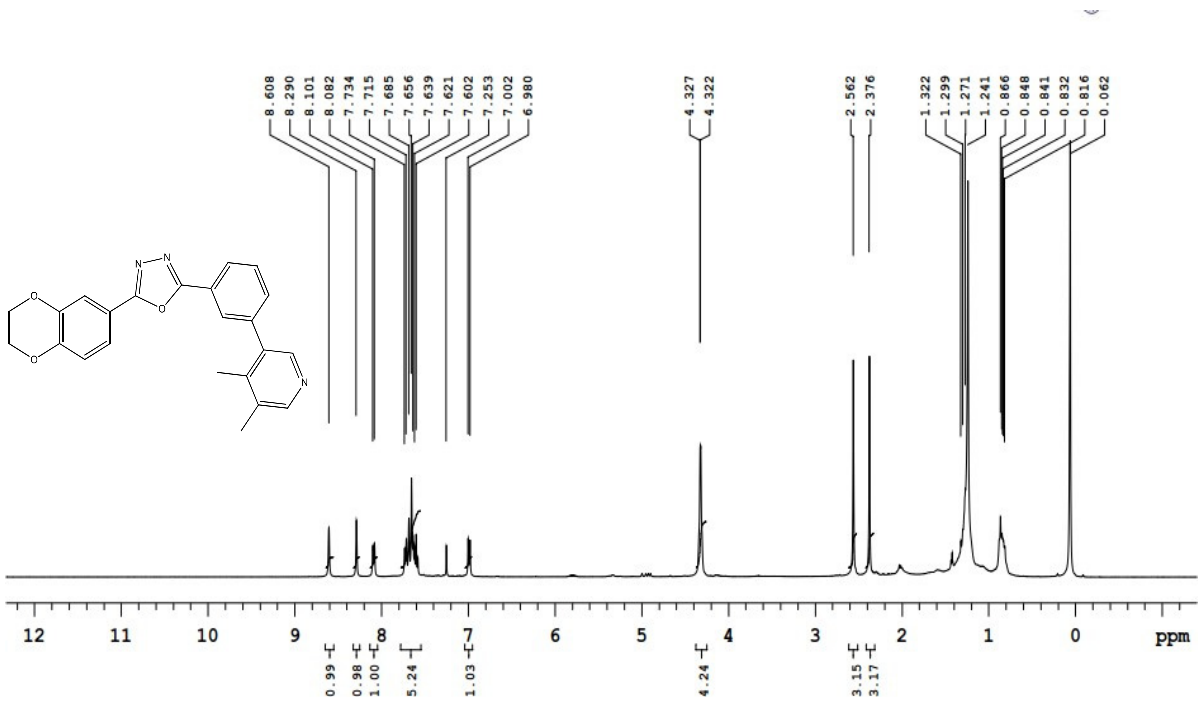

^{1}H NMR spectrum of 5k

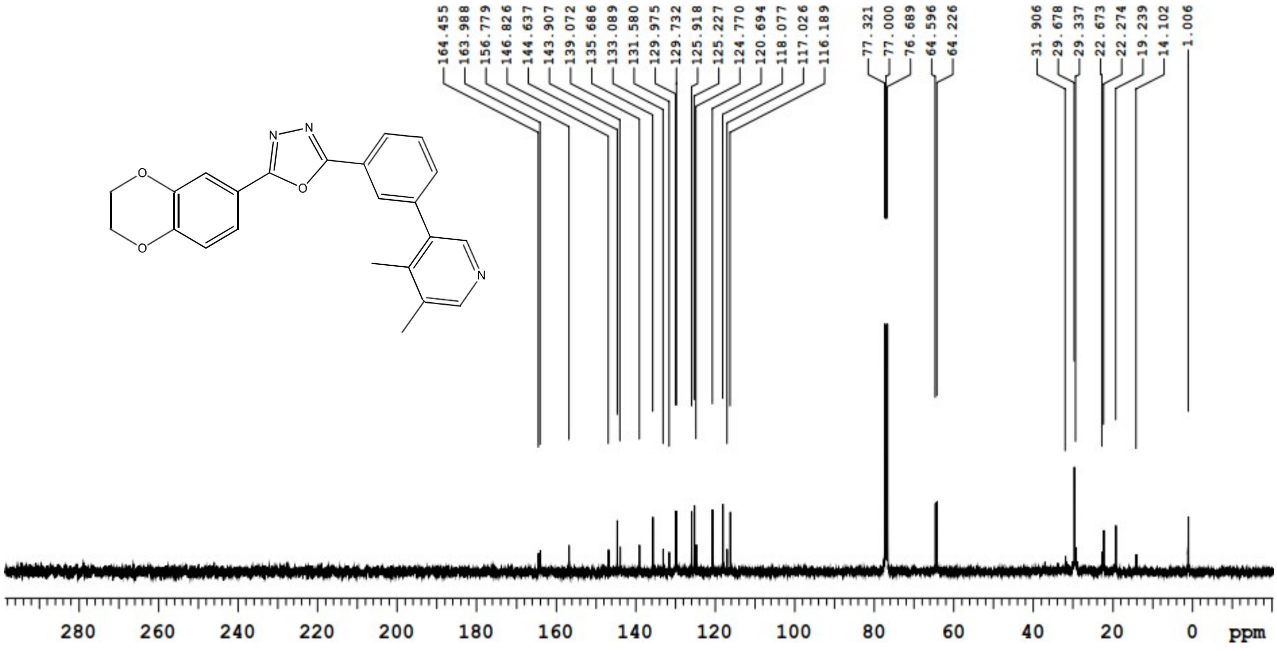

^{13}C NMR spectrum of 5k

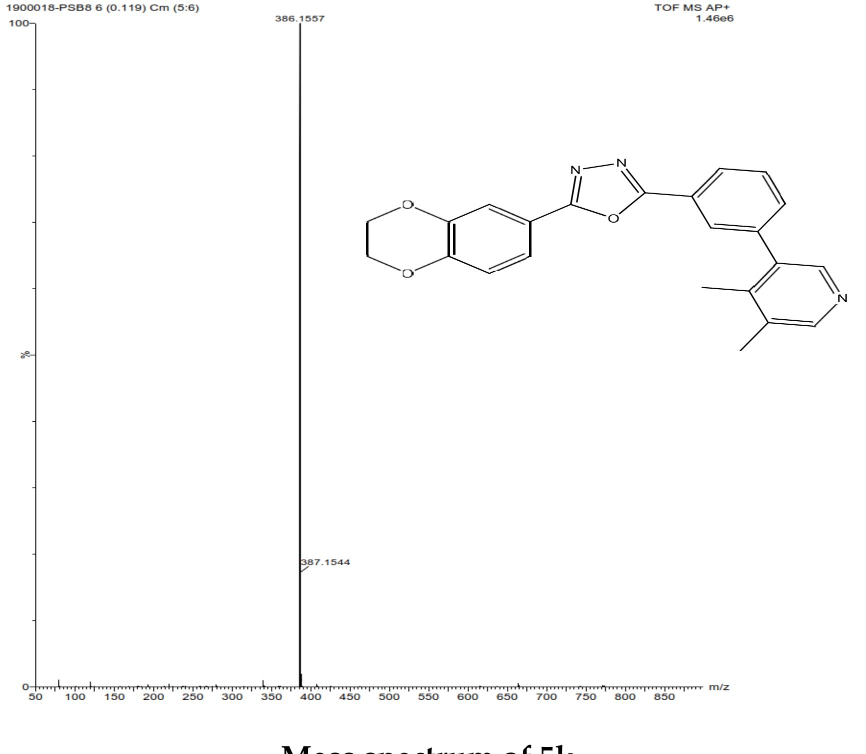

Mass spectrum of 5k

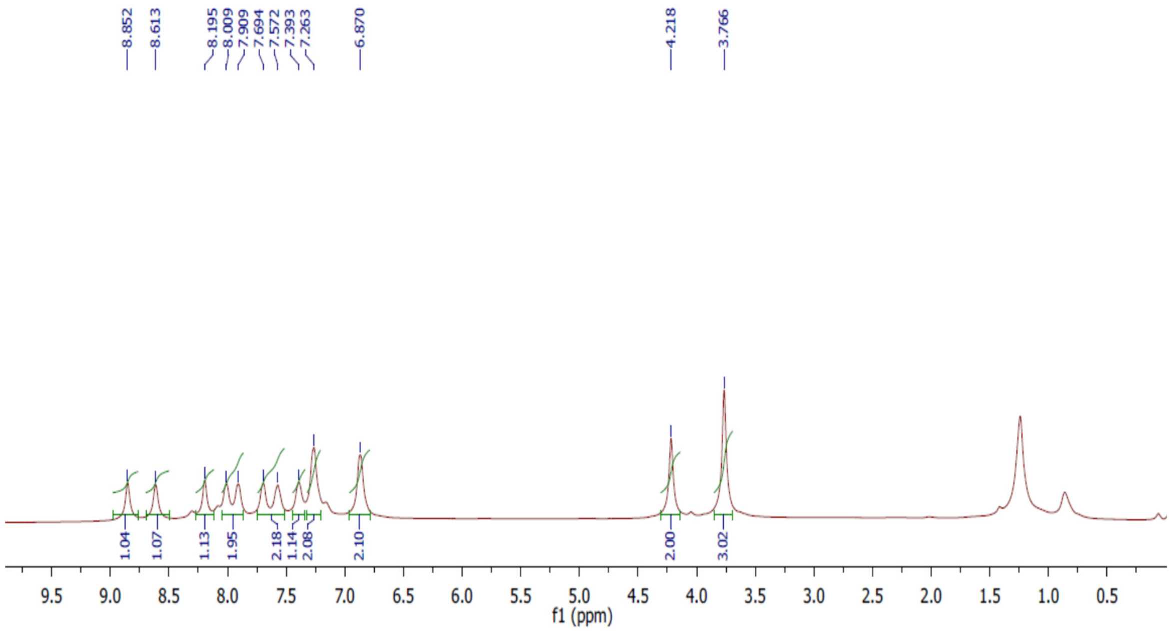

^{1}H NMR spectrum of 5l

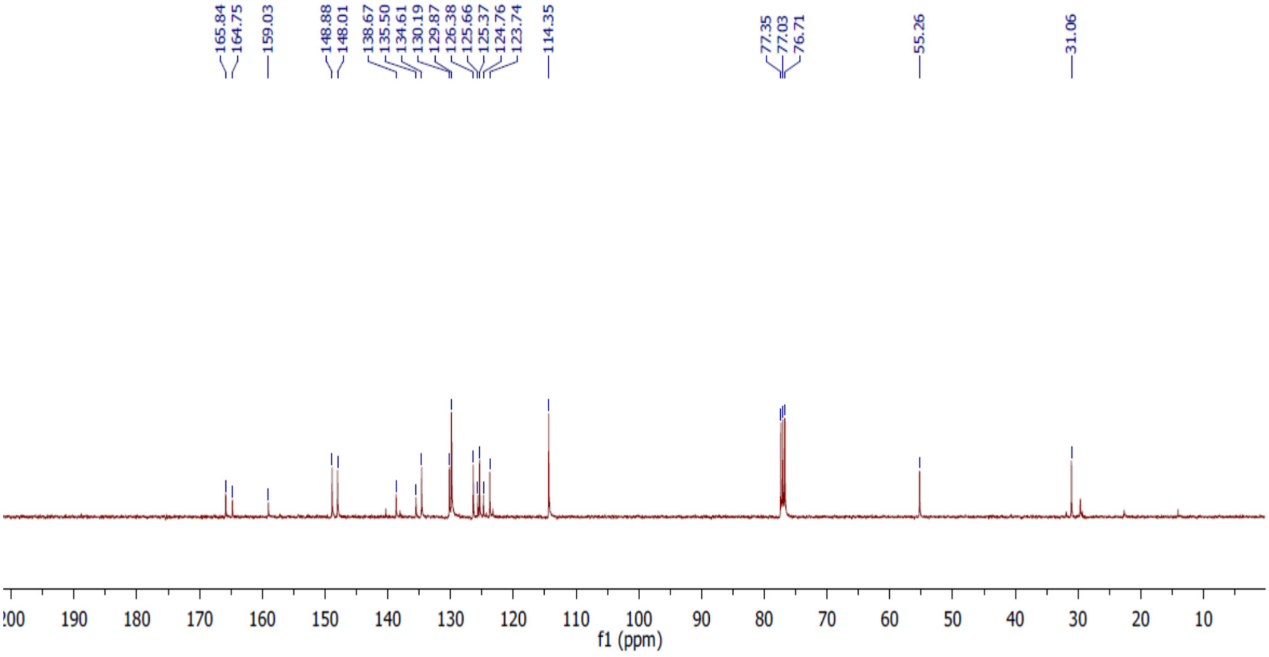

^{13}C NMR spectrum of 5l

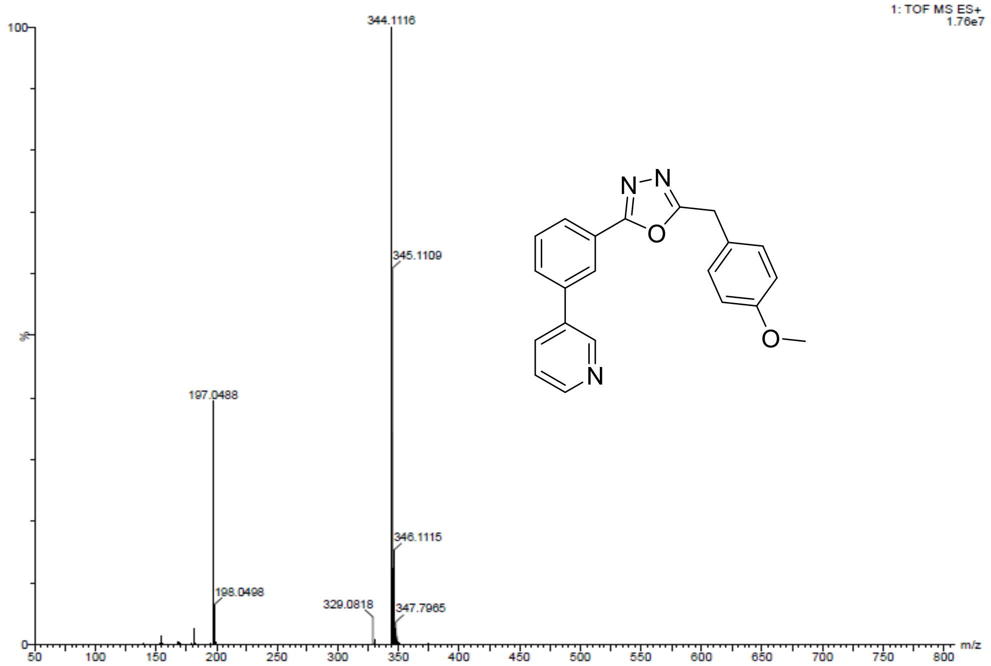

Mass spectrum of 5l

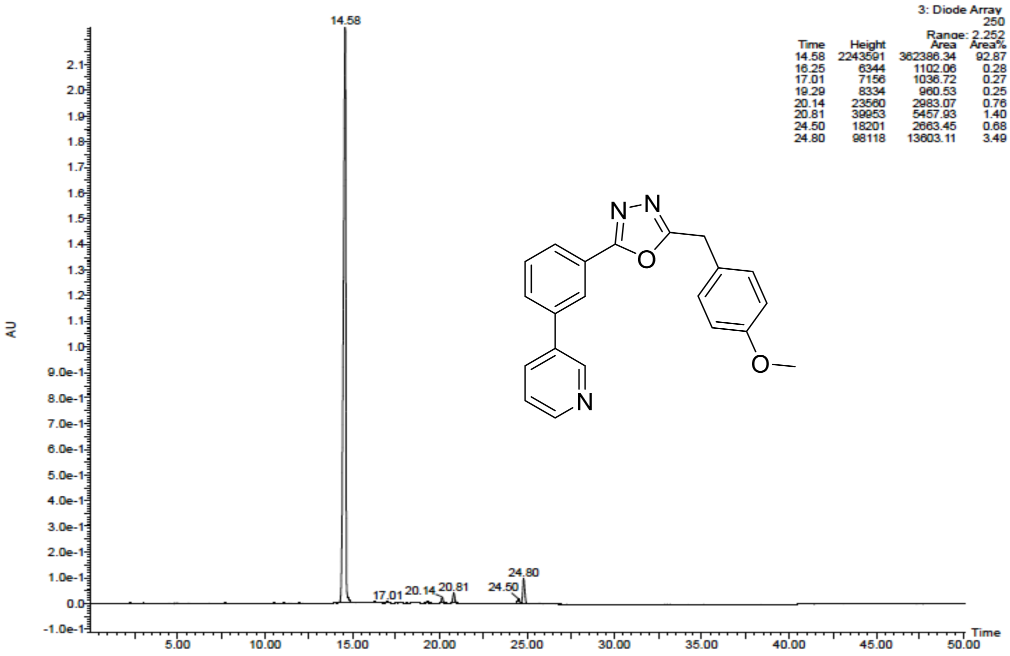

Time	Height	Area	Area%
14.58	2243591	362386.34	92.87
16.25	6344	1102.06	0.28
17.01	7156	1036.72	0.27
19.29	8334	960.53	0.25
20.14	23560	2983.07	0.76
20.81	39953	5457.93	1.40
24.50	18201	2663.45	0.68
24.80	98118	13603.11	3.49

HPLC of 5l

- Cell line: MCF7 (2000 cells/per well[96])
- Treated time: 72hrs
- Assay: MTT (90mins incubated)
- Data: 5l

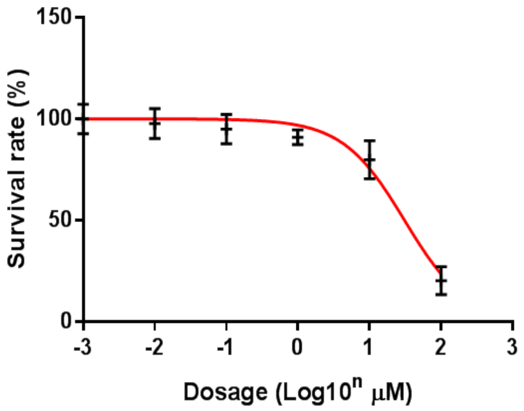

IC_{50} (μM) = 30.85
Log IC_{50} (μM) = 1.49

Conc. (μM)	Viability	
	AVE.	± SD.
0	100.00	7.25
0.01	97.71	7.35
0.1	94.95	7.22
1	90.96	3.61
10	79.84	9.35
100	20.22	6.91

Log curve for the compound 5l

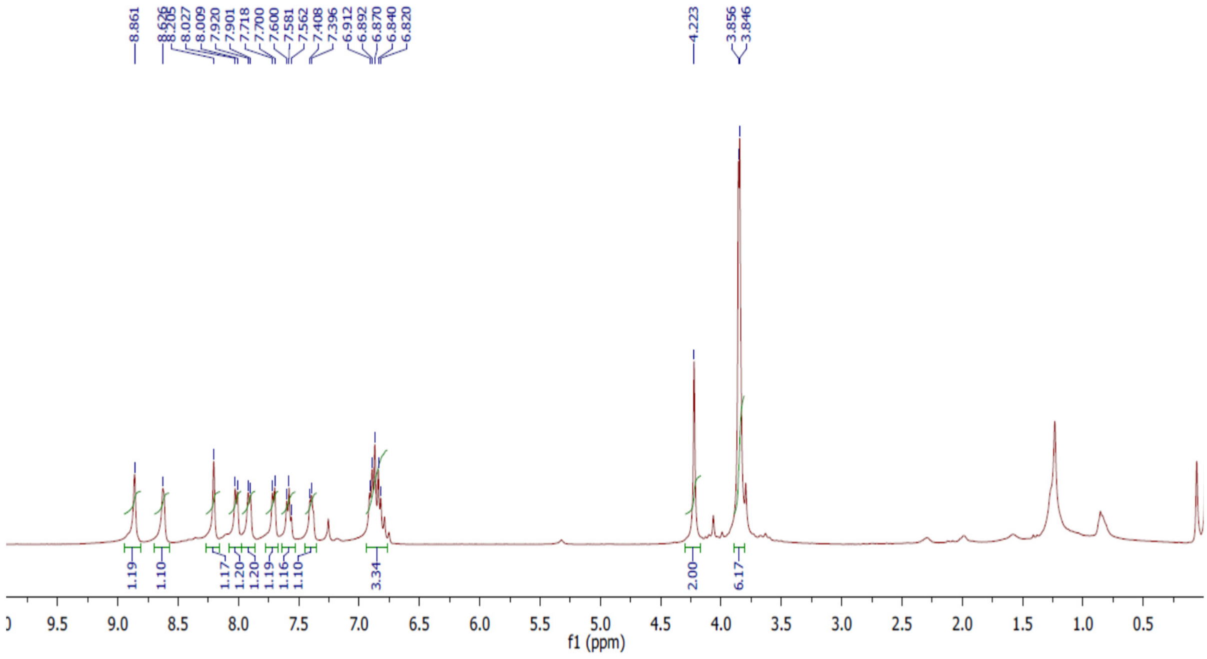

^{1}H NMR spectrum of 5m

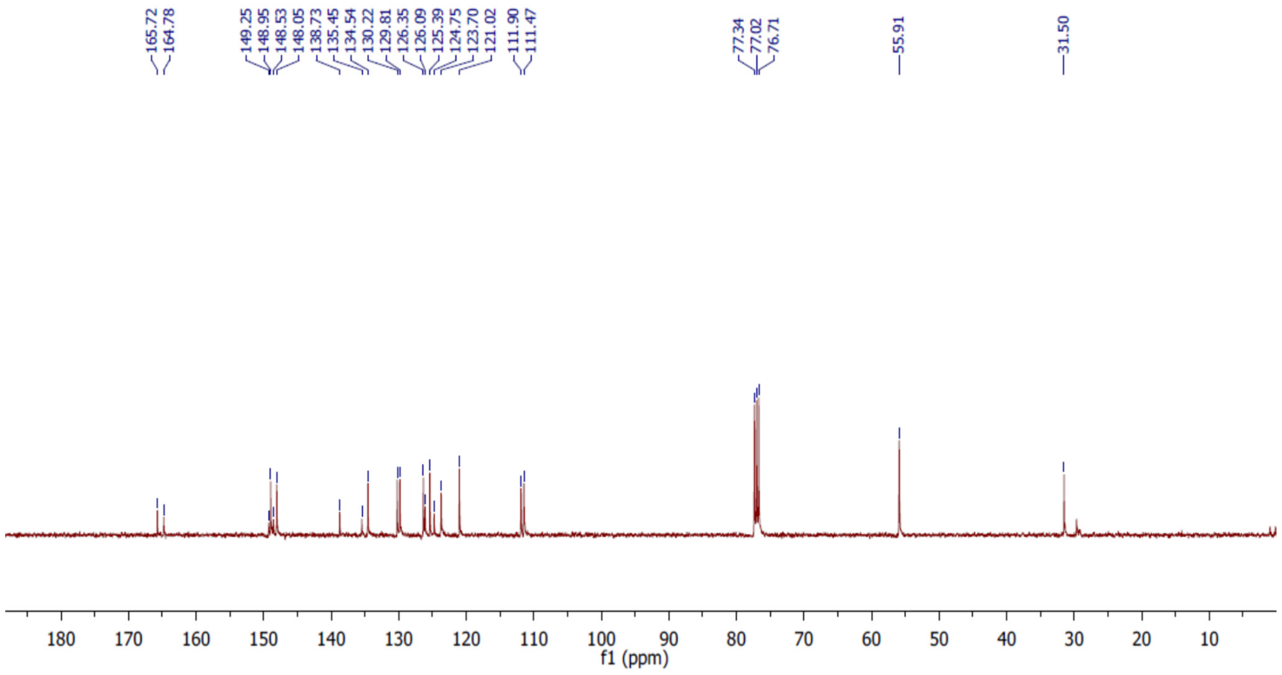

^{13}C NMR spectrum of 5m

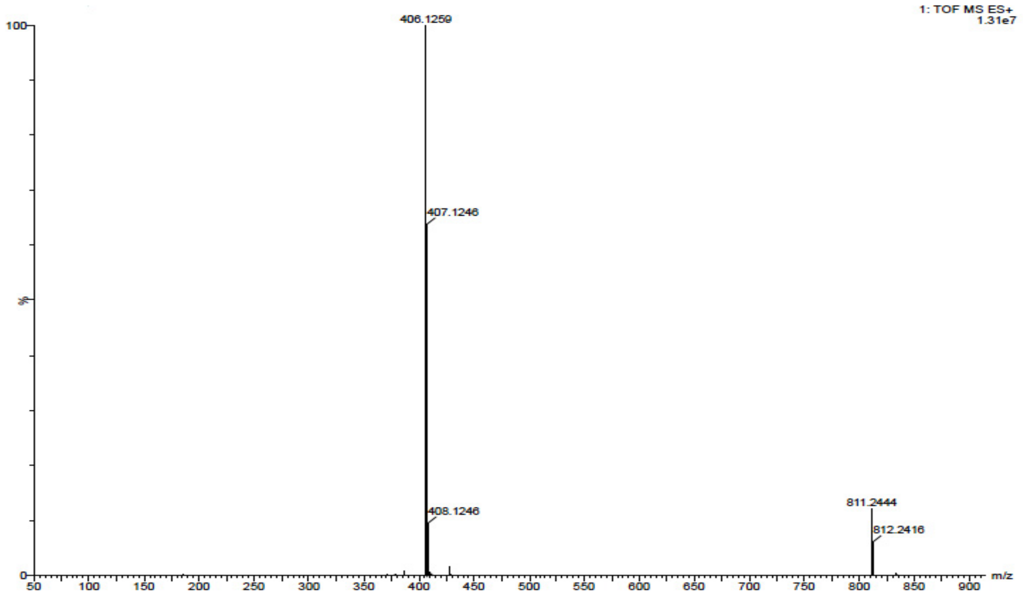

Mass spectrum of 5m

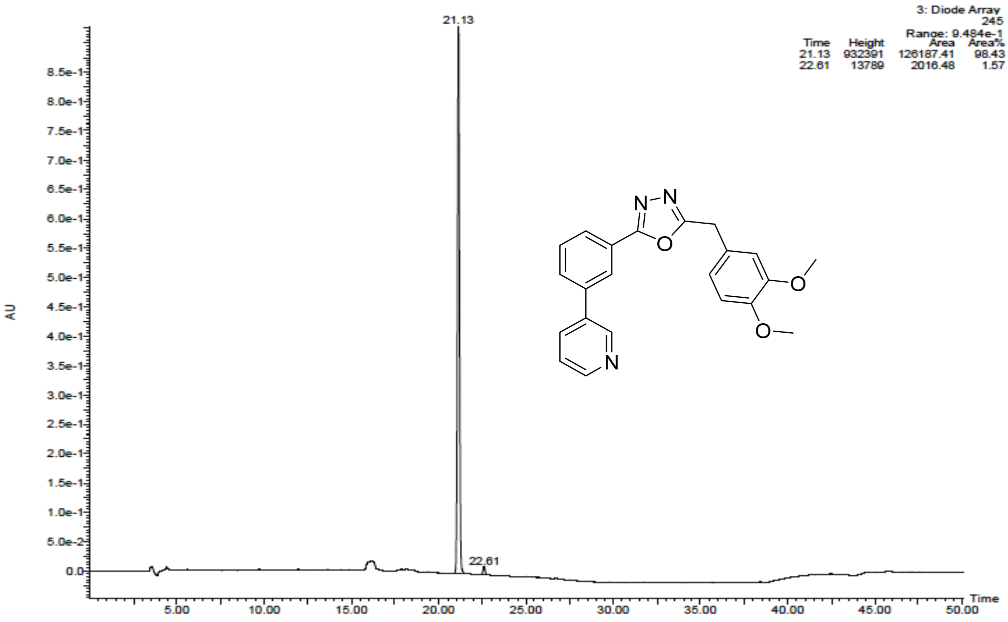

HPLC of 5m

- Cell line: MCF7 (2000 cells/per well[96])
- Treated time: 72hrs
- Assay: MTT (90mins incubated)
- Data: 5m

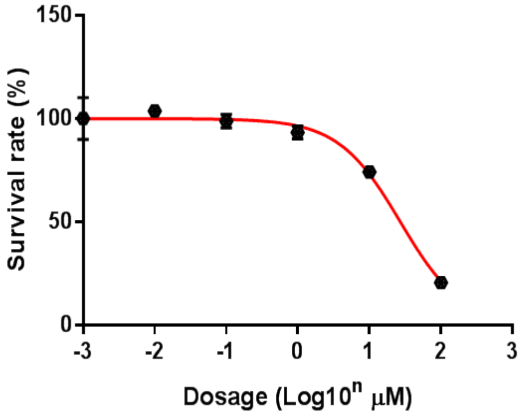

IC$_{50}$ (μM) = 27.13
Log IC$_{50}$ (μM) = 1.43

Conc. (μM)	Viability	
	AVE.	± SD.
0	100.00	10.16
0.01	103.64	1.22
0.1	98.80	3.31
1	93.32	3.10
10	74.25	3.03
100	20.61	1.96

Log curve for the compound 5m

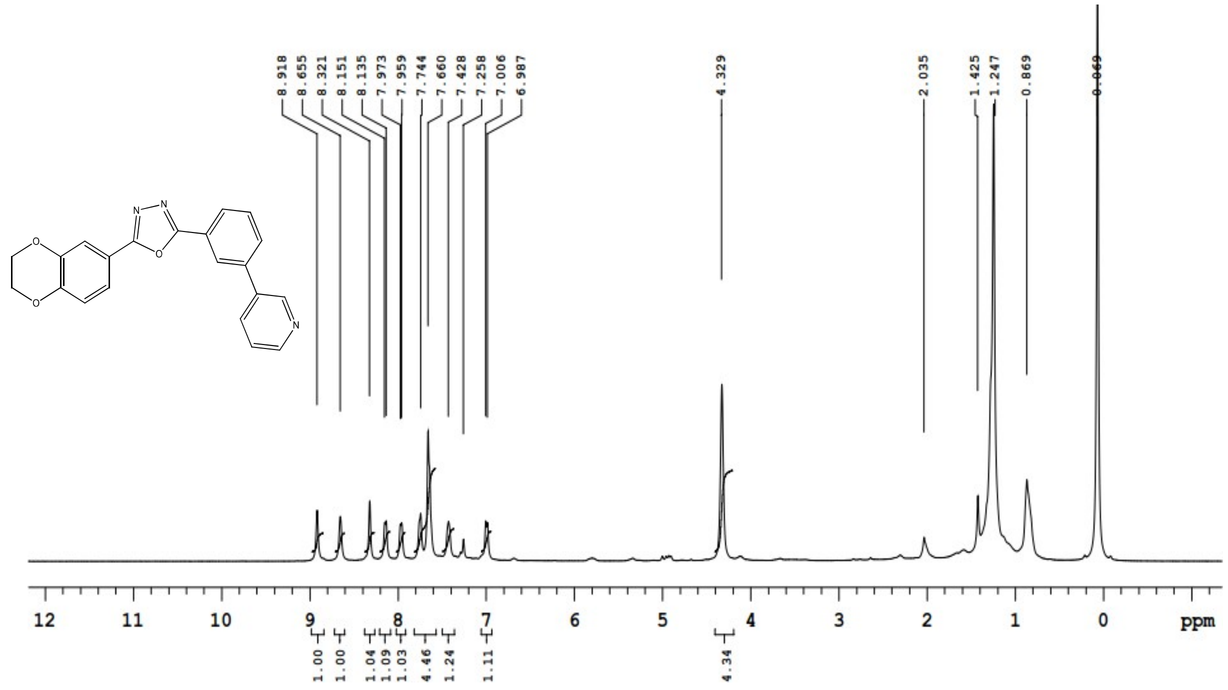

^{1}H NMR spectrum of 5n

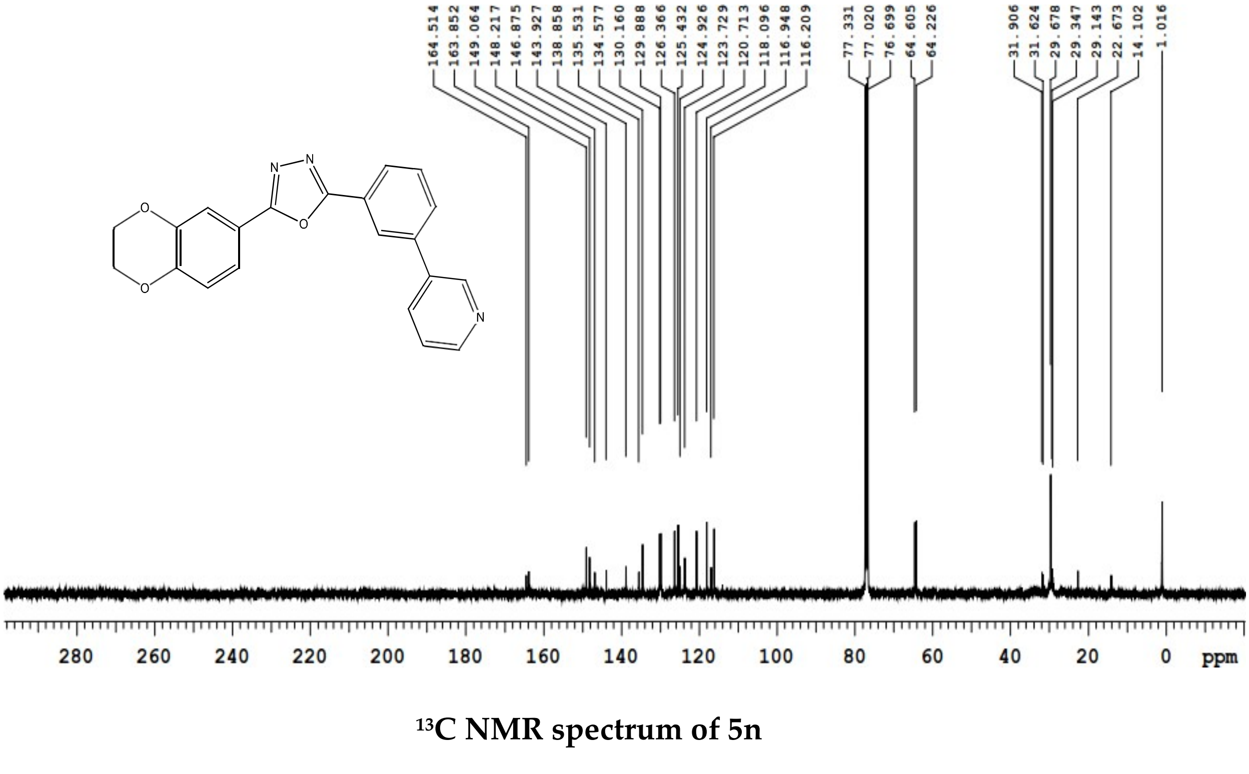

^{13}C NMR spectrum of 5n

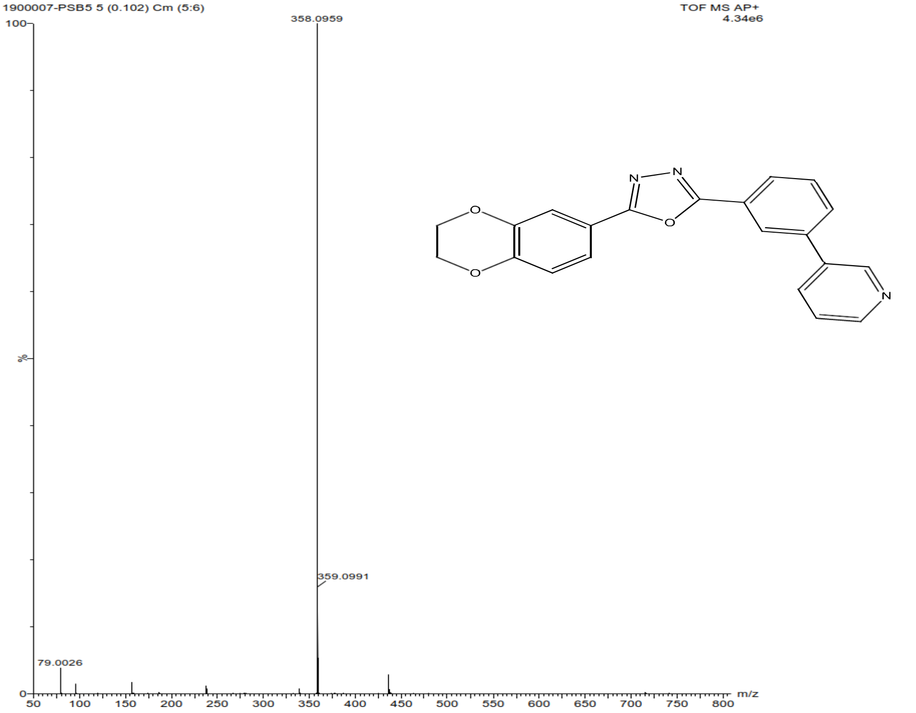

Mass spectrum of 5n

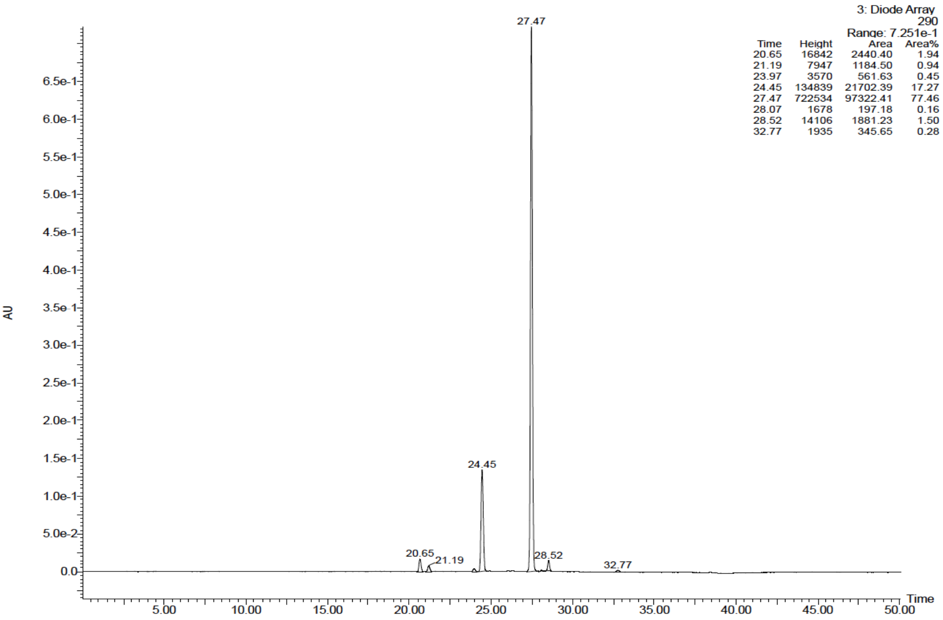

HPLC of 5n

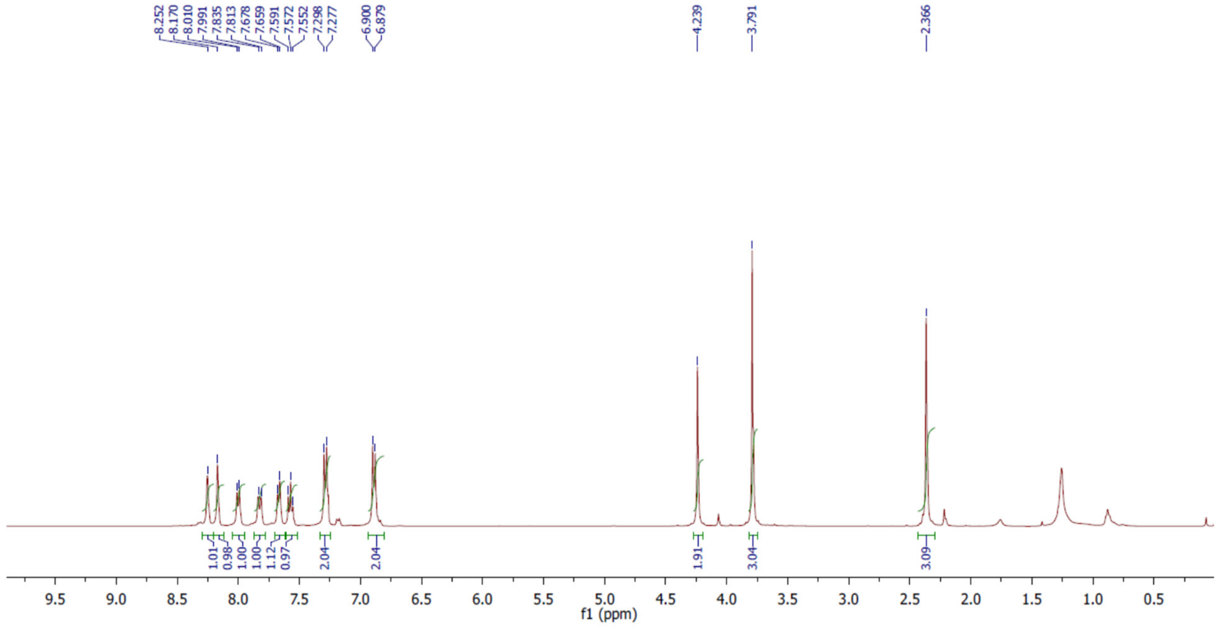

^{1}H NMR spectrum of 5o

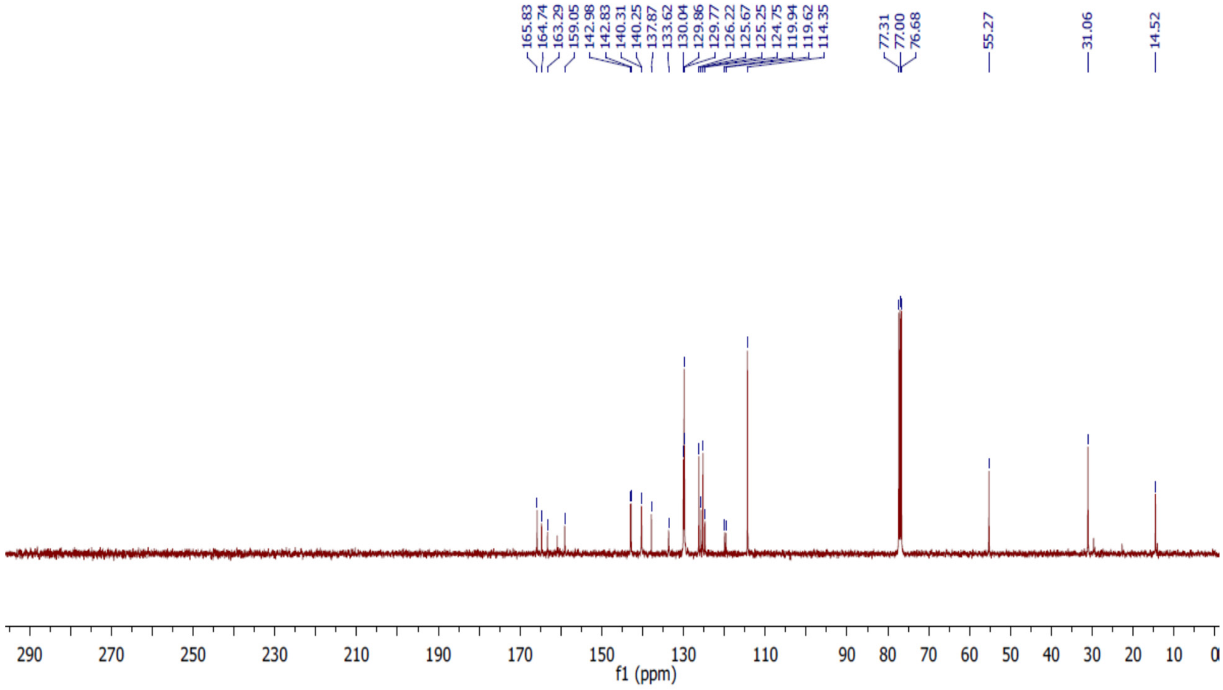

^{13}C NMR spectrum of 5o

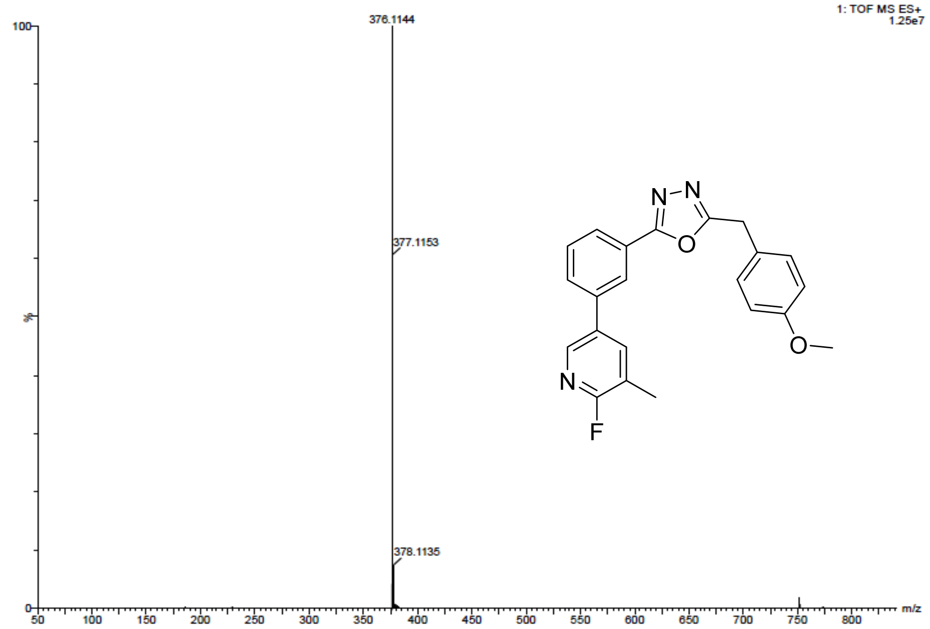

Mass spectrum of 5o

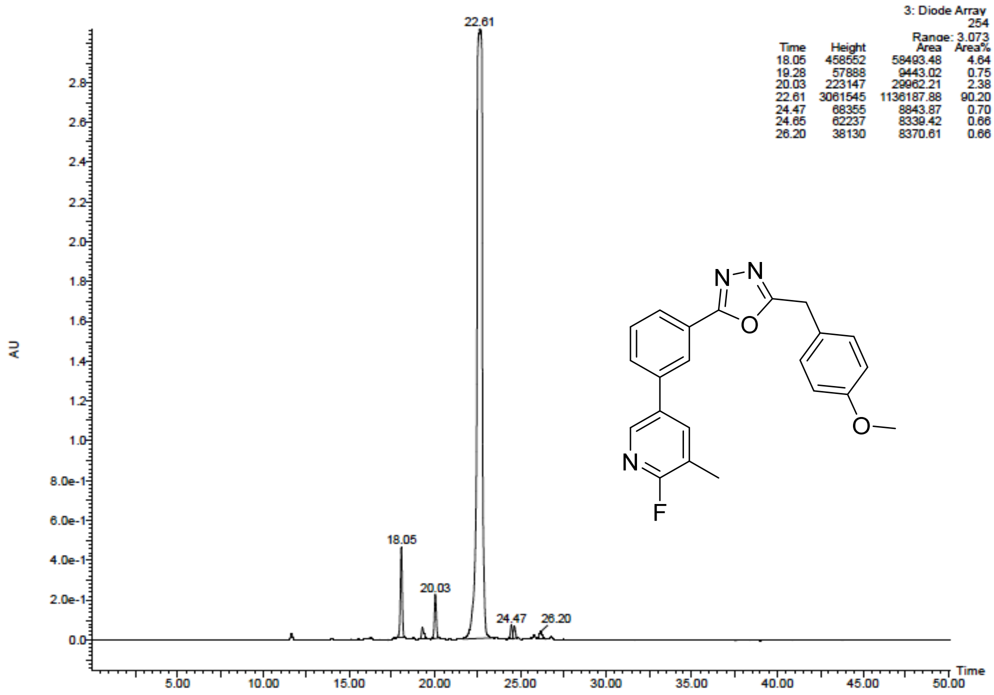

HPLC of 5o

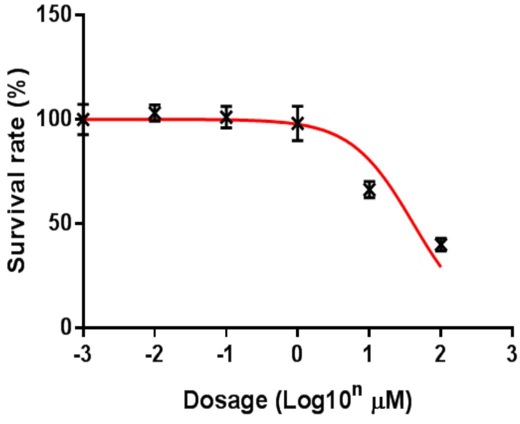

IC$_{50}$ (μM) = 41.23
Log IC$_{50}$ (μM) = 1.62

- Cell line: MCF7 (2000 cells/per well[96])
- Treated time: 72hrs
- Assay: MTT (90mins incubated)
- Data: 5o

Conc. (μM)	Viability	
	AVE.	± SD.
**0**	100.00	7.25
**0.01**	103.08	3.85
**0.1**	101.02	5.22
**1**	98.02	8.25
**10**	66.33	3.83
**100**	39.89	3.02

Log curve for the compound 5o

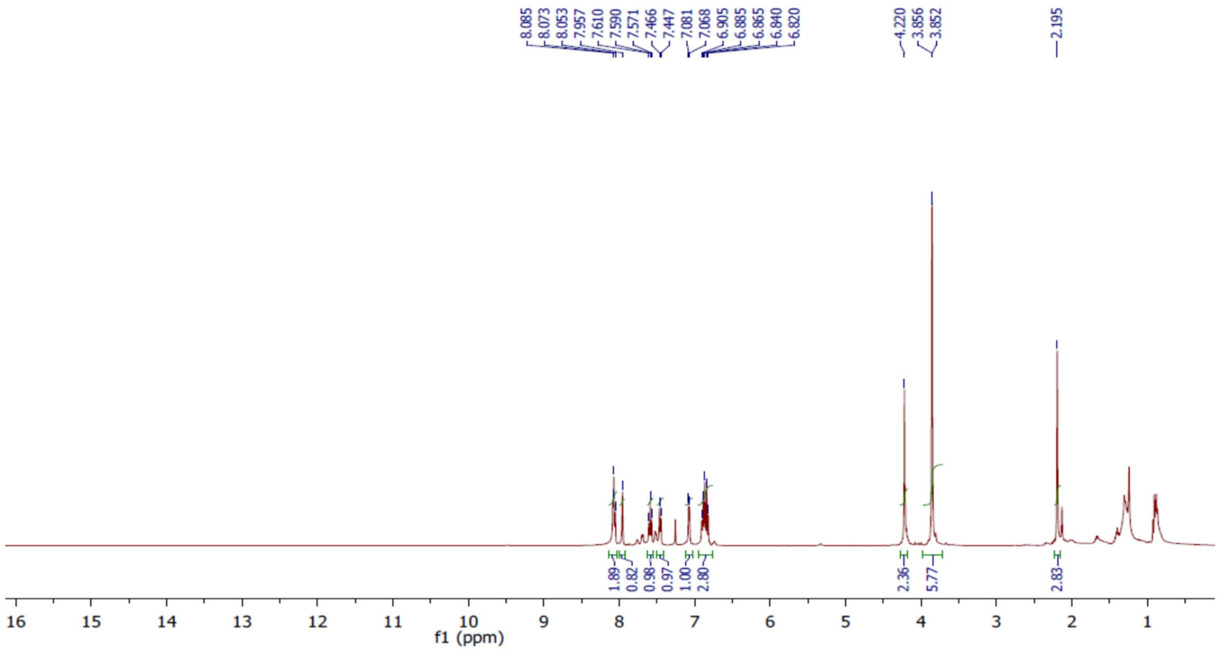

^{1}H NMR spectrum of 5p

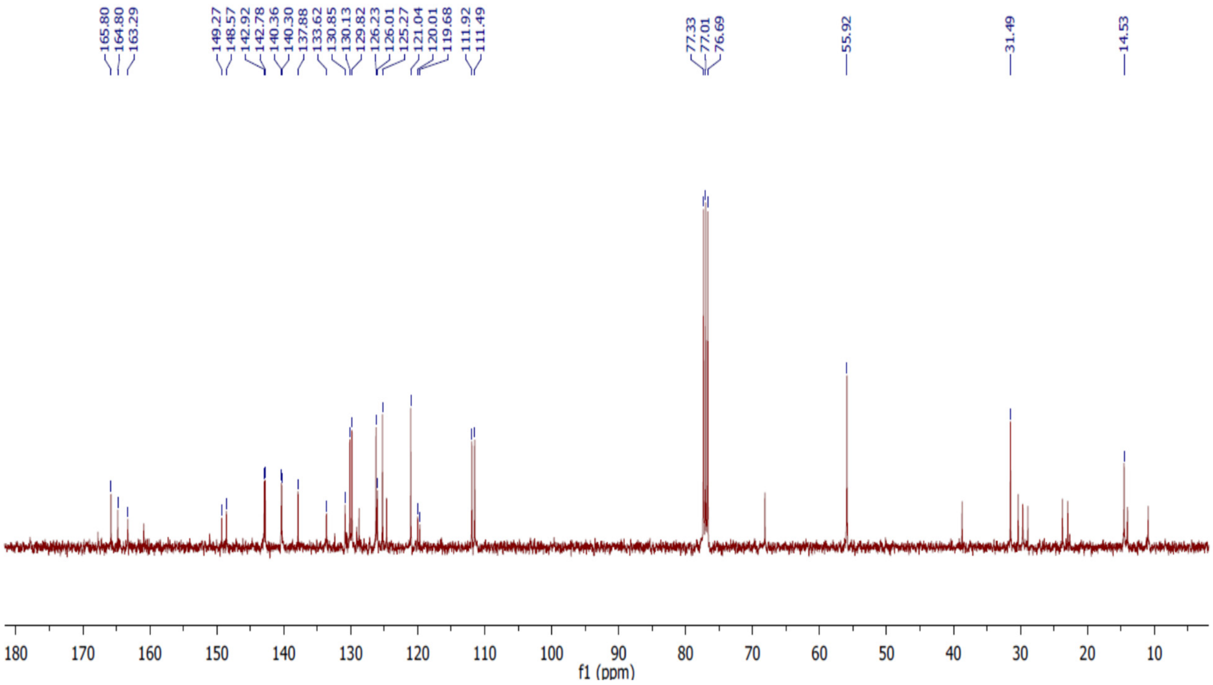

^{13}C NMR spectrum of 5p

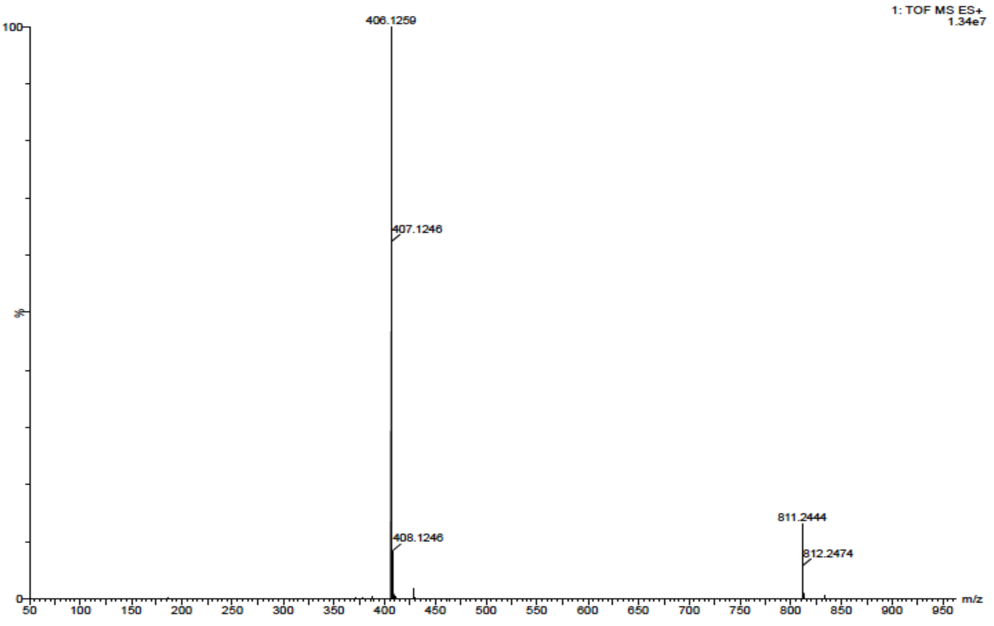

Mass spectrum of 5p

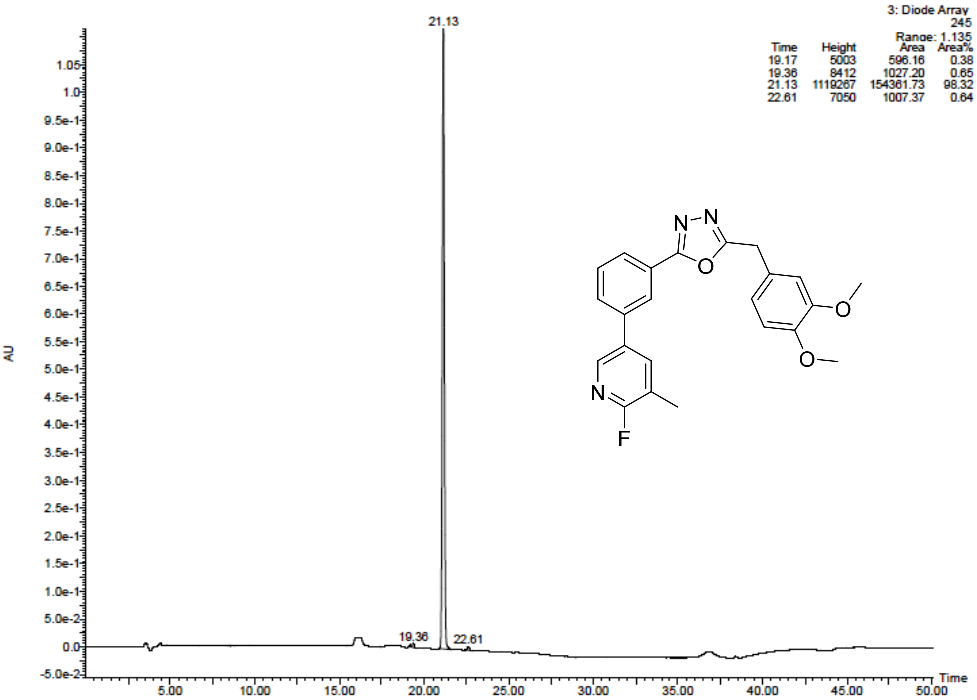

HPLC of 5p

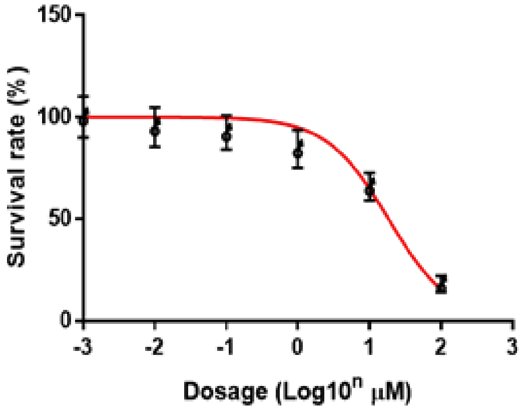

IC_{50} (μM) = 18.45
Log IC_{50} (μM) = 1.27

- Cell line: MCF7 (2000 cells/per well[96])
- Treated time: 72hrs
- Assay: MTT (90mins incubated)
- Data: 5p

Conc. (μM)	Viability	
	AVE.	± SD.
**0**	100.00	10.16
**0.01**	95.08	9.70
**0.1**	92.46	8.41
**1**	84.35	9.33
**10**	65.82	6.74
**100**	18.11	3.99

Log curve for the compound 5p

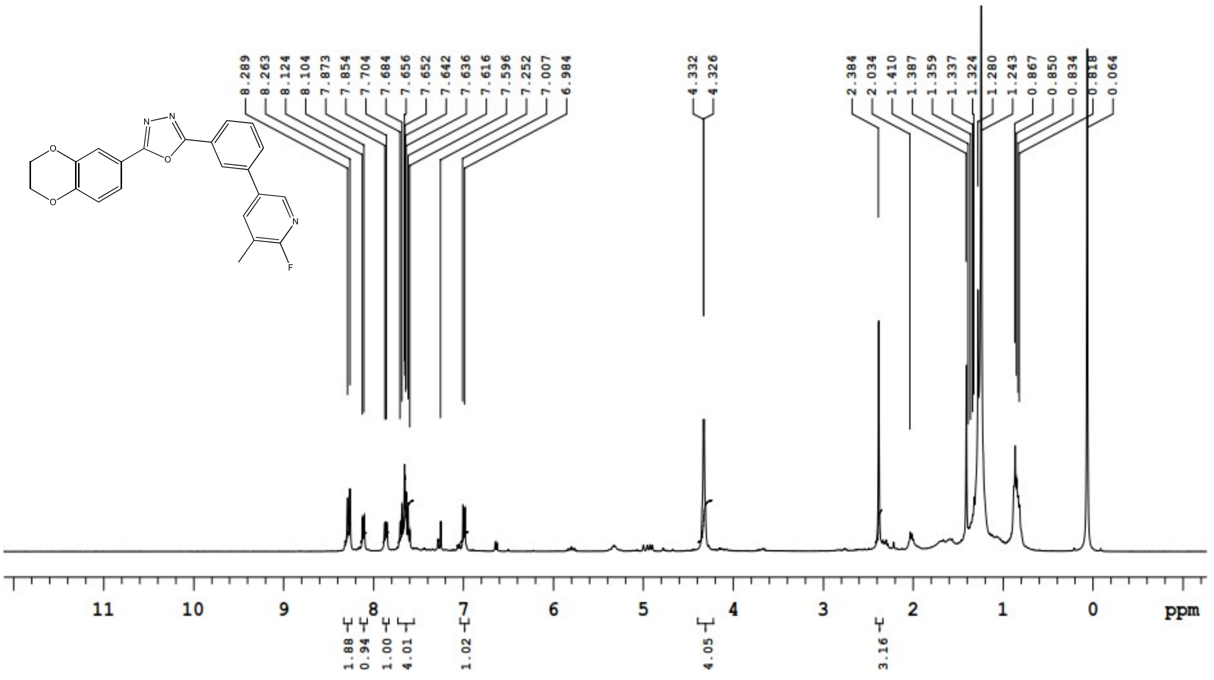

^{1}H NMR spectrum of 5q

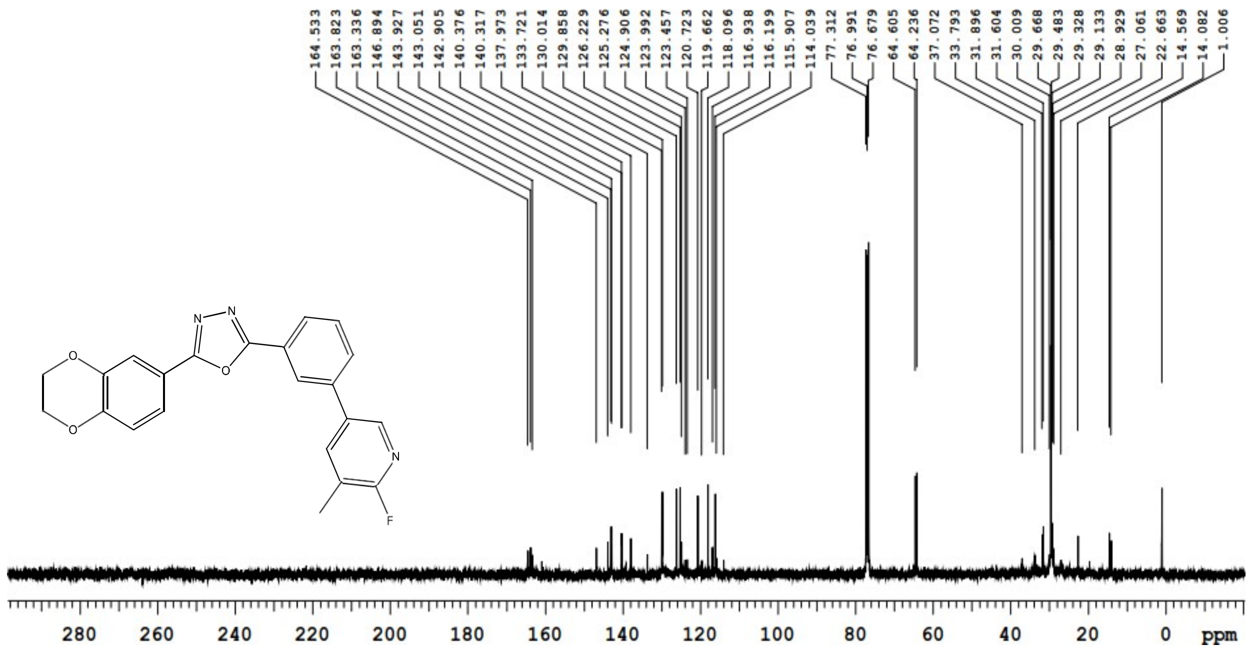

^{13}C NMR spectrum of 5q

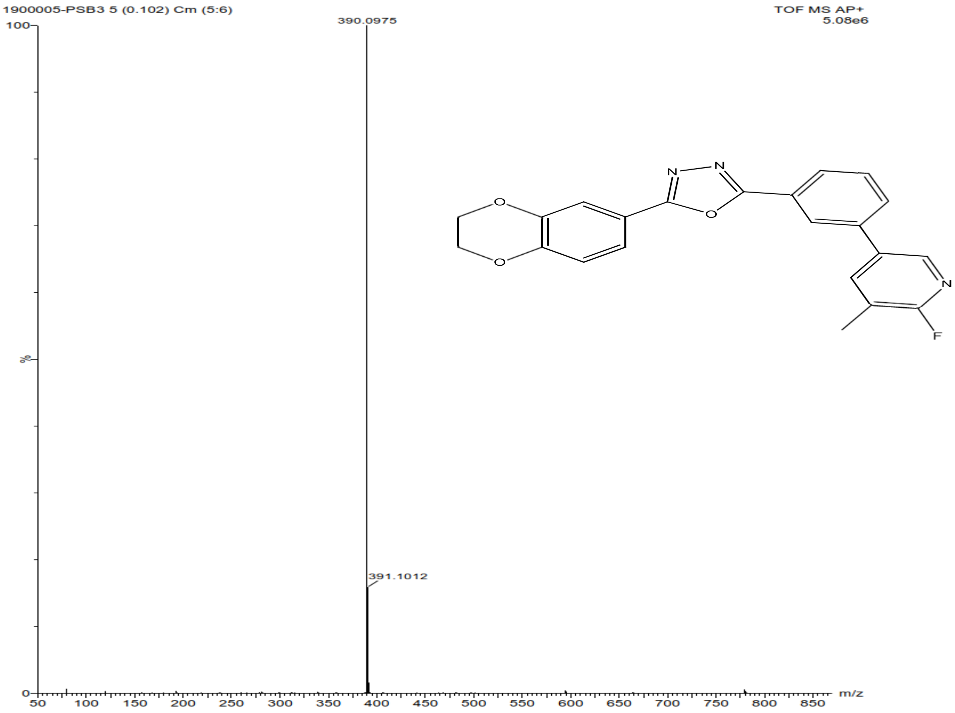

Mass spectrum of 5q

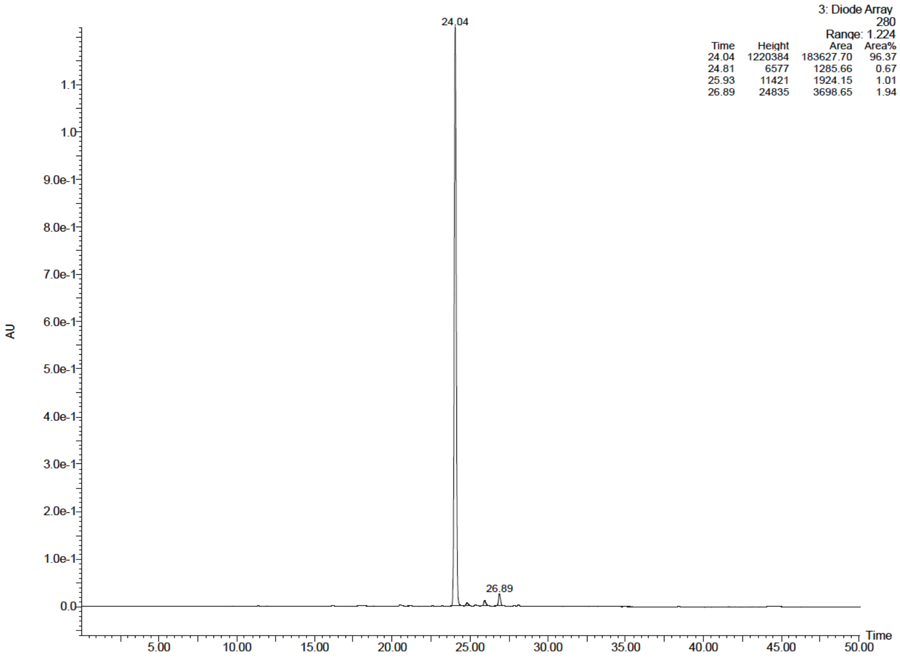

HPLC of 5q

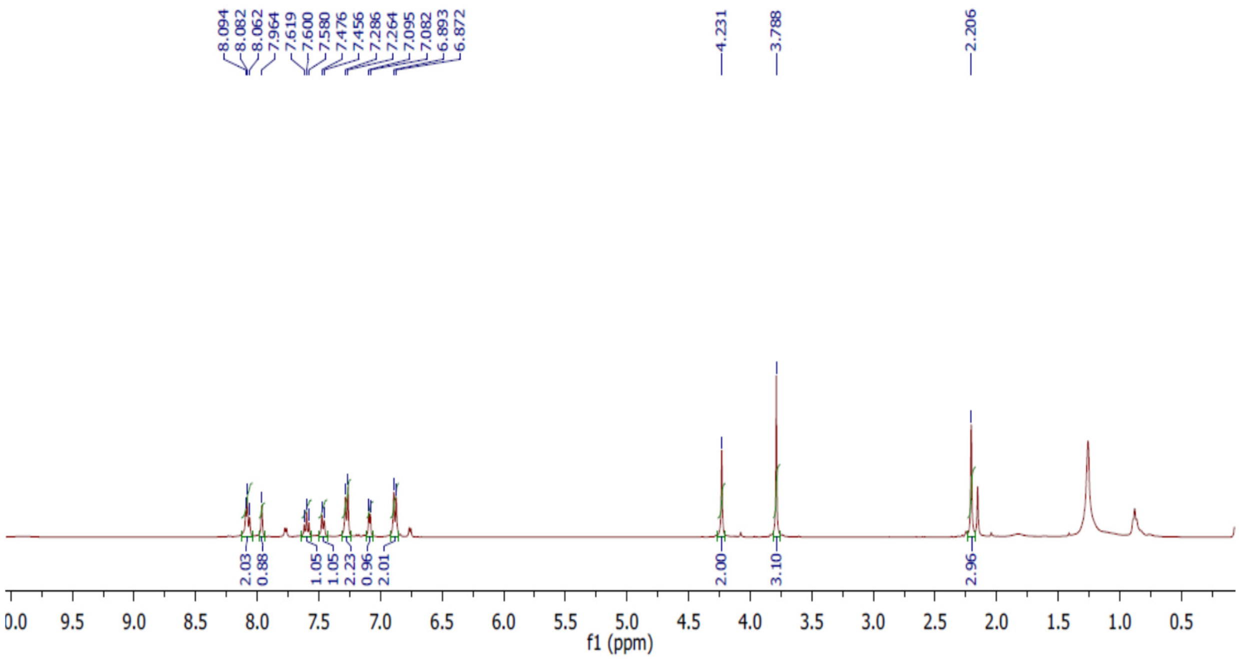

^{1}H NMR spectrum of 5r

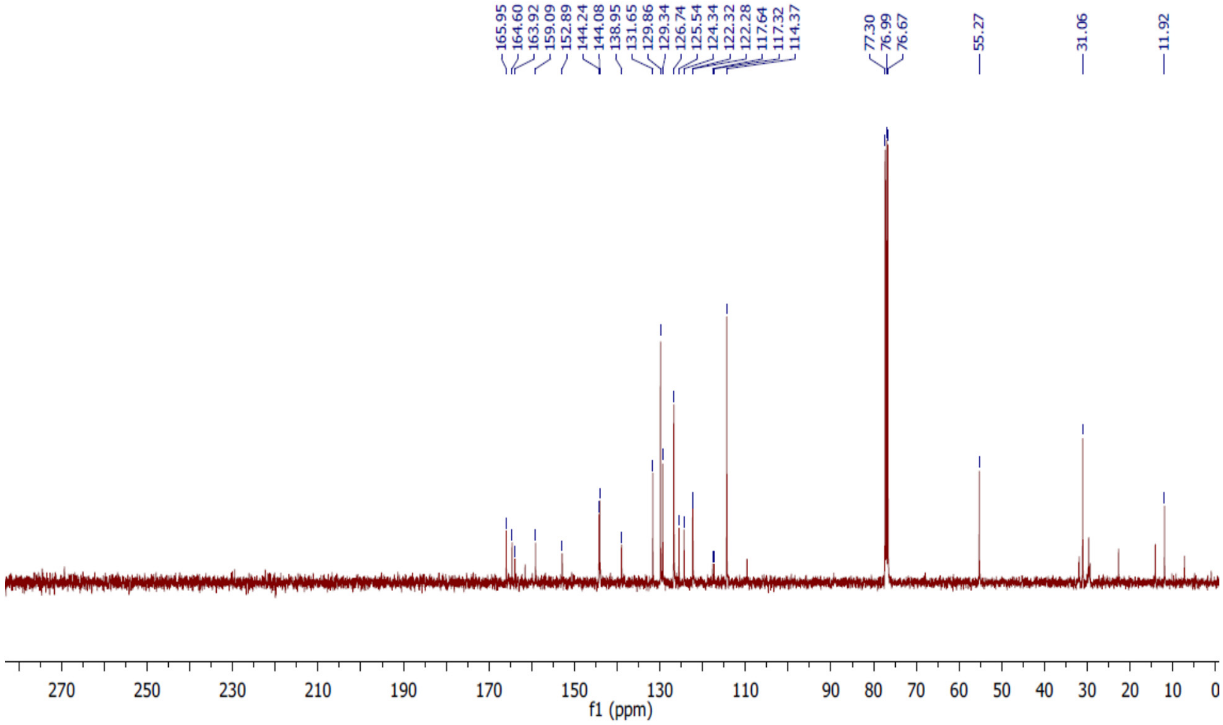

^{13}C NMR spectrum of 5r

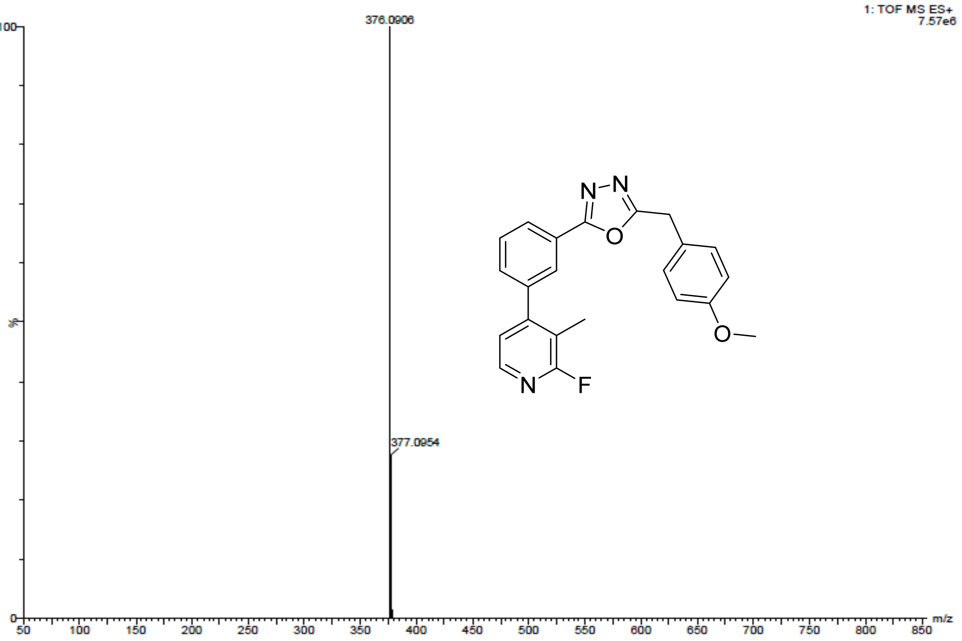

Mass spectrum of 5r

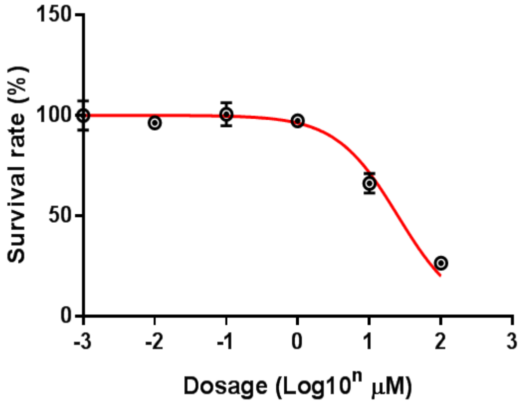

IC_{50} (μM) = 25.04
Log IC_{50} (μM) = 1.40

- Cell line: MCF7 (2000 cells/per well[96])
- Treated time: 72hrs
- Assay: MTT (90mins incubated)
- Data: 5r

Conc. (μM)	Viability	
	AVE.	± SD.
**0**	100.00	7.25
**0.01**	96.26	2.85
**0.1**	100.62	5.74
**1**	97.28	2.19
**10**	66.21	4.88
**100**	26.43	0.30

Log curve for the compound 5r

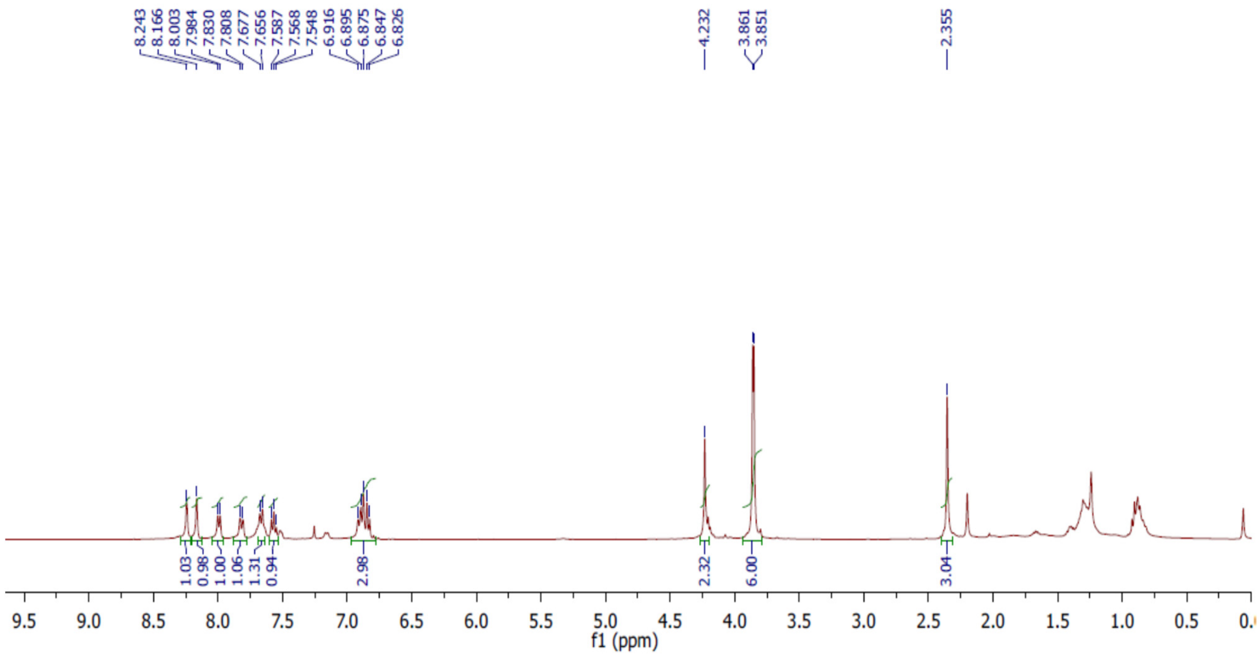

^{1}H NMR spectrum of 5s

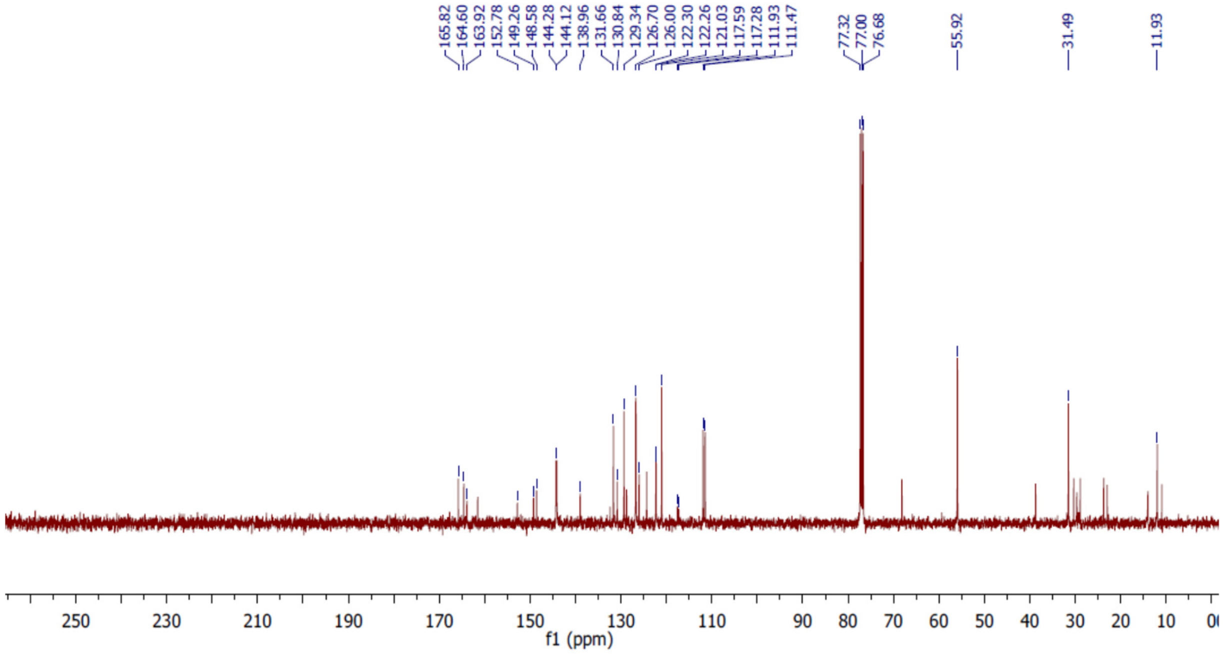

^{13}C NMR spectrum of 5s

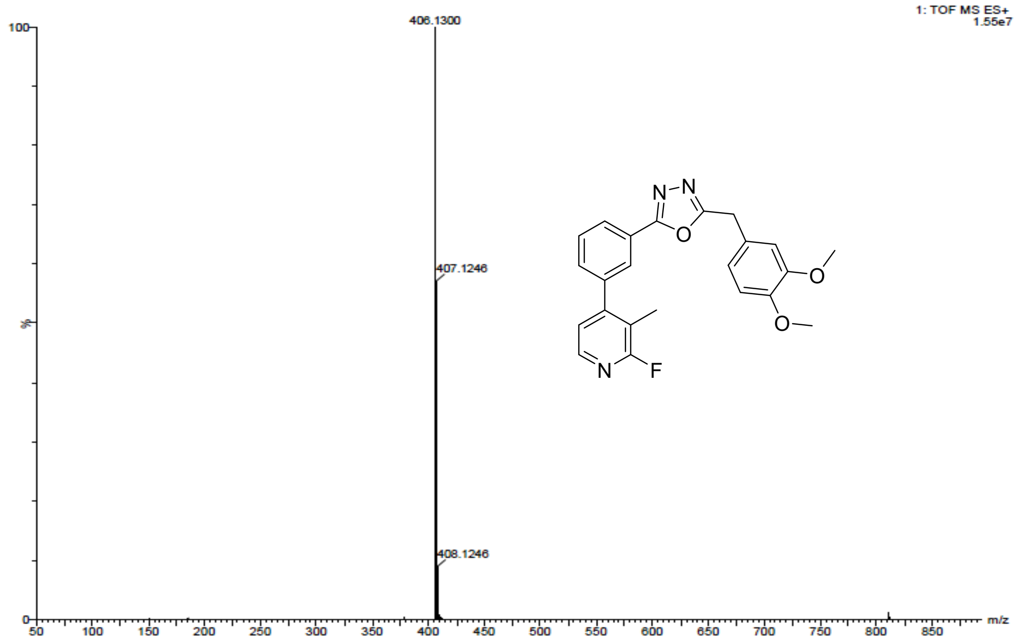

Mass spectrum of 5s

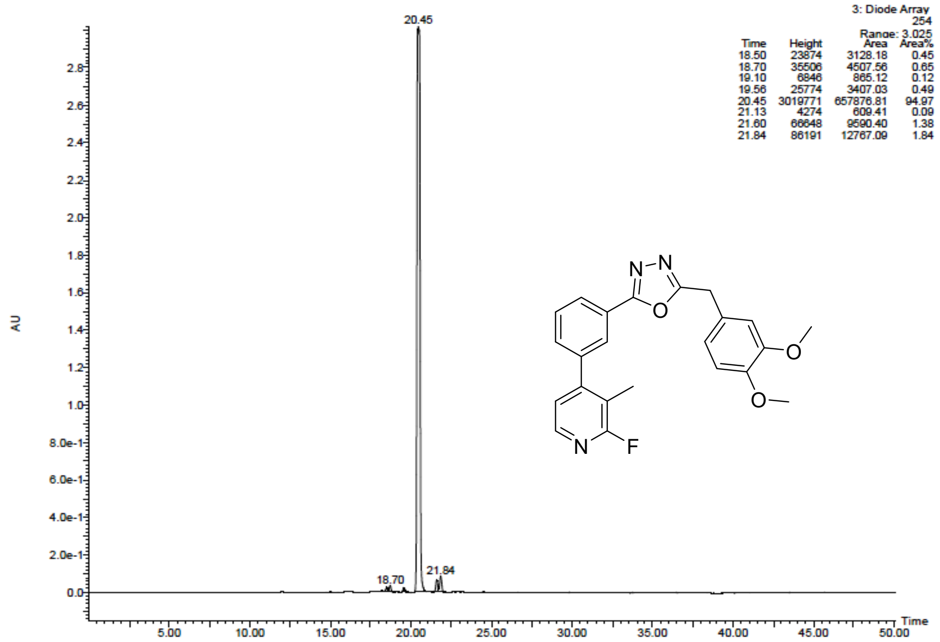

Time	Height	Area	Area%
18.50	23874	3128.18	0.45
18.70	35506	4507.56	0.65
19.10	6846	865.12	0.12
19.56	25774	3407.03	0.49
20.45	3019771	657876.81	94.97
21.13	4274	609.41	0.09
21.60	66648	9590.40	1.38
21.84	86191	12767.09	1.84

HPLC of 5s

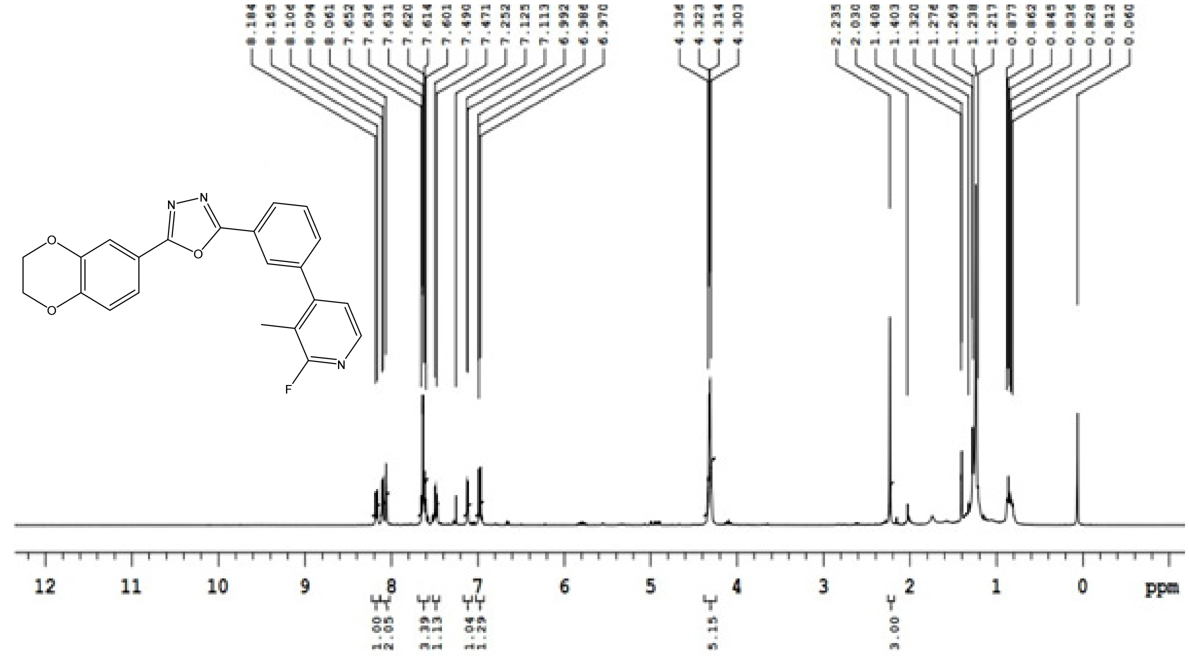

^{1}H NMR spectrum of 5t

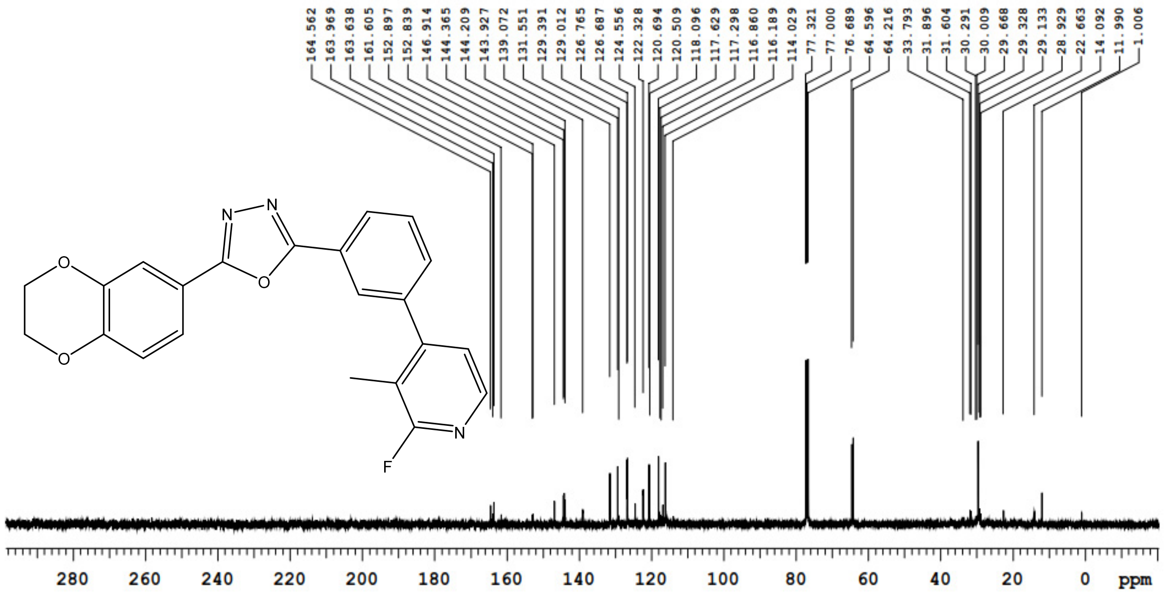

^{13}C NMR spectrum of 5t

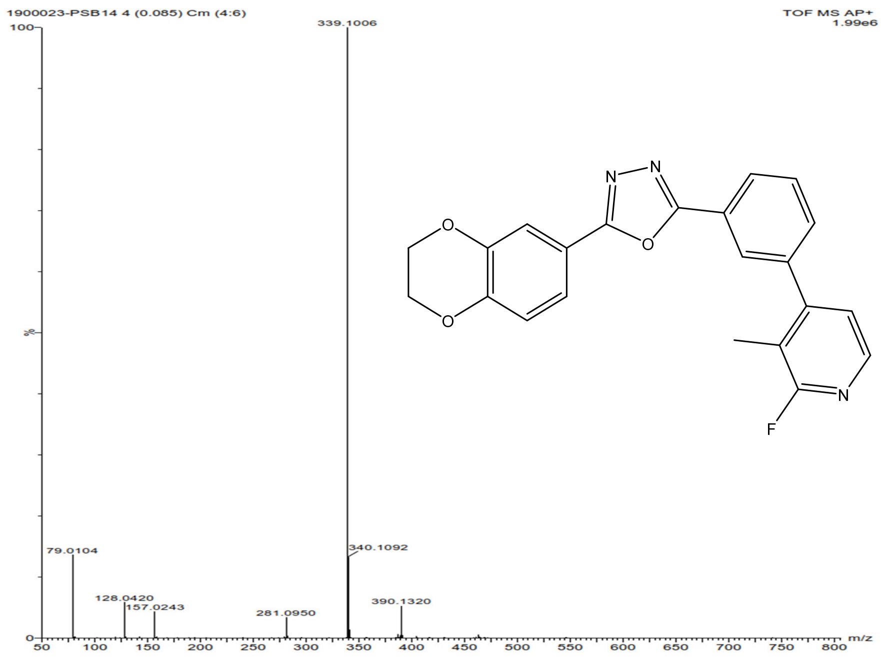

Mass spectrum of 5t

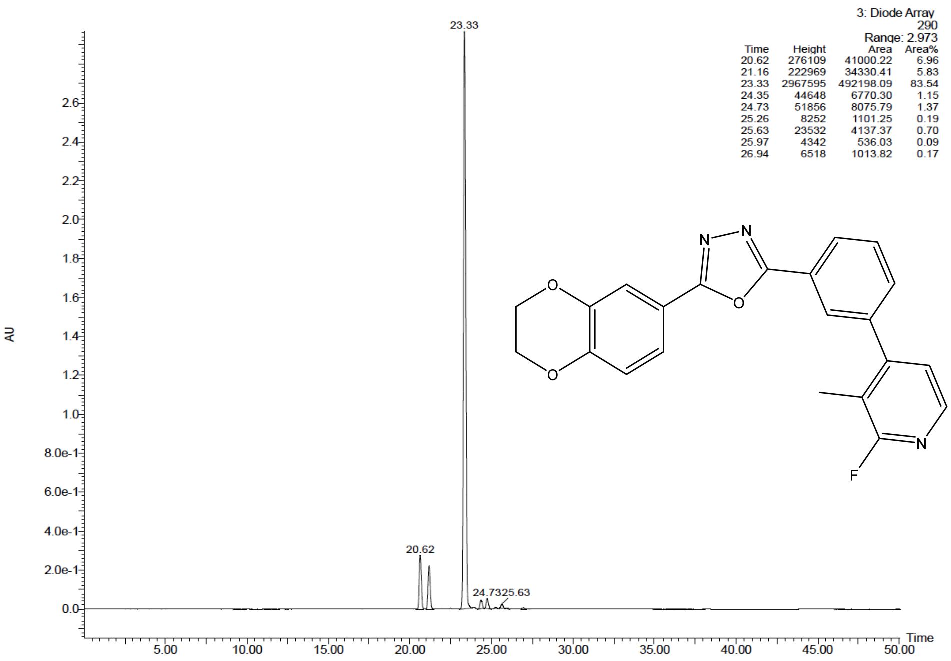

HPLC of 5t

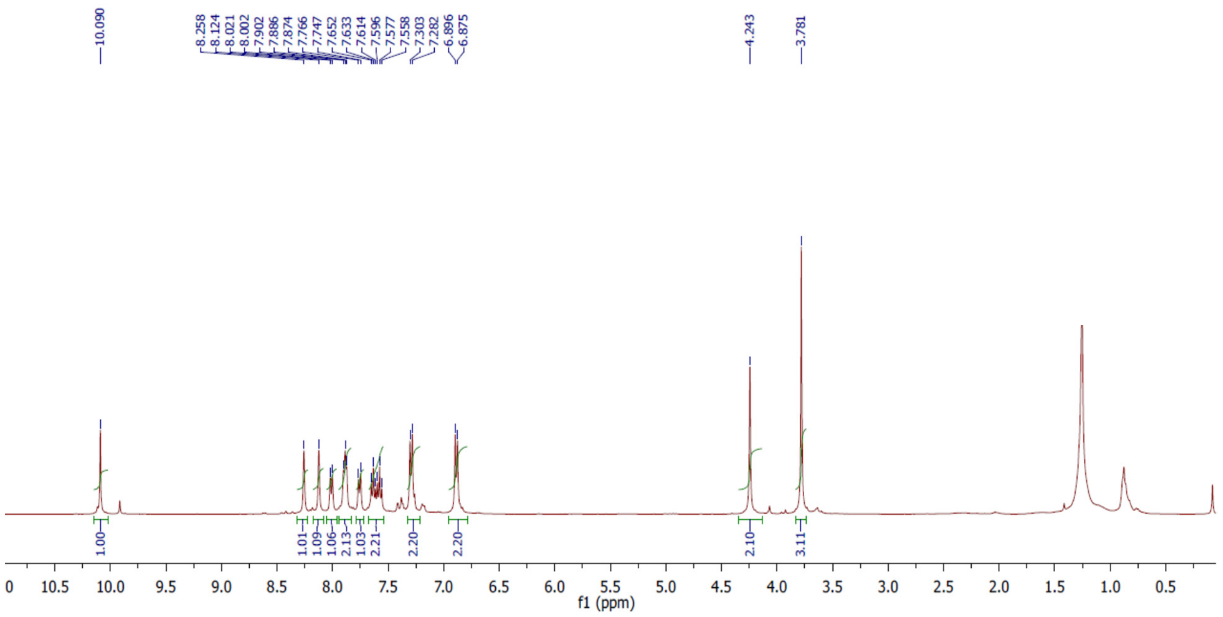

^{1}H NMR spectrum of 5u

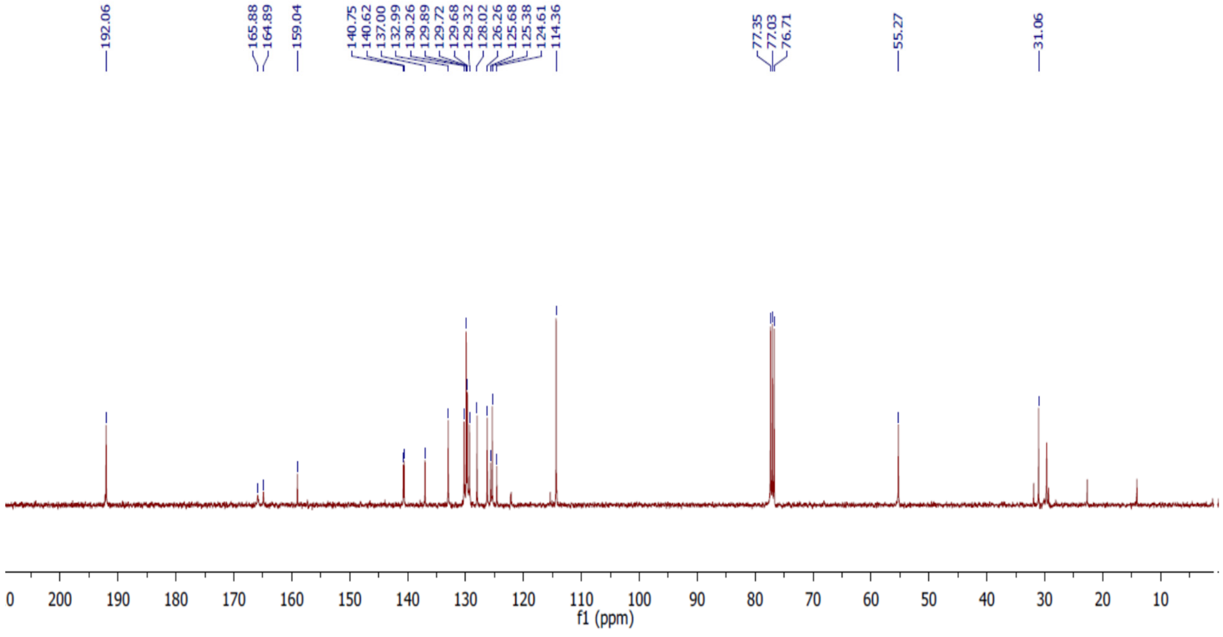

^{13}C NMR spectrum of 5u

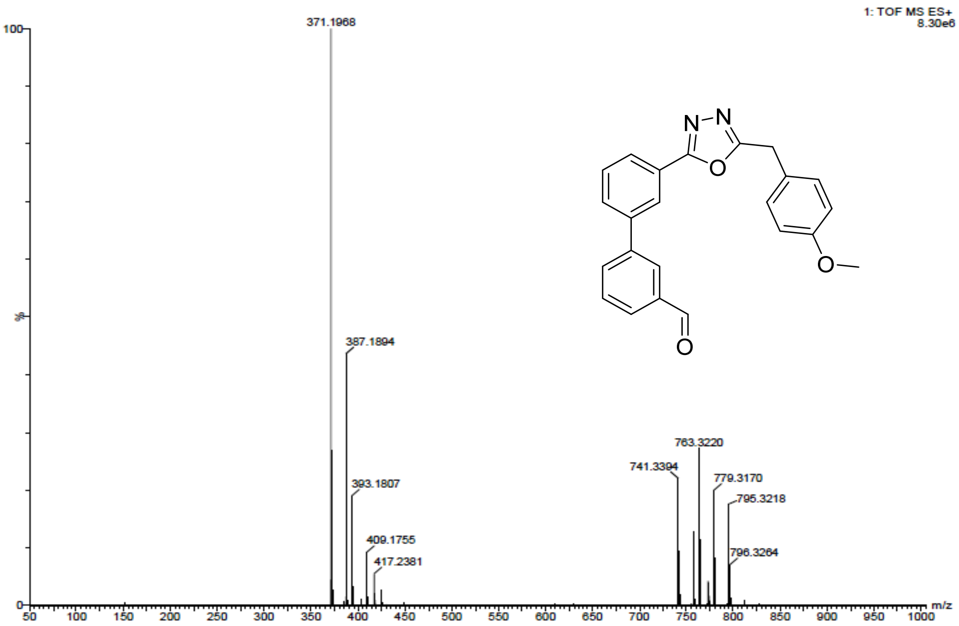

Mass spectrum of 5u

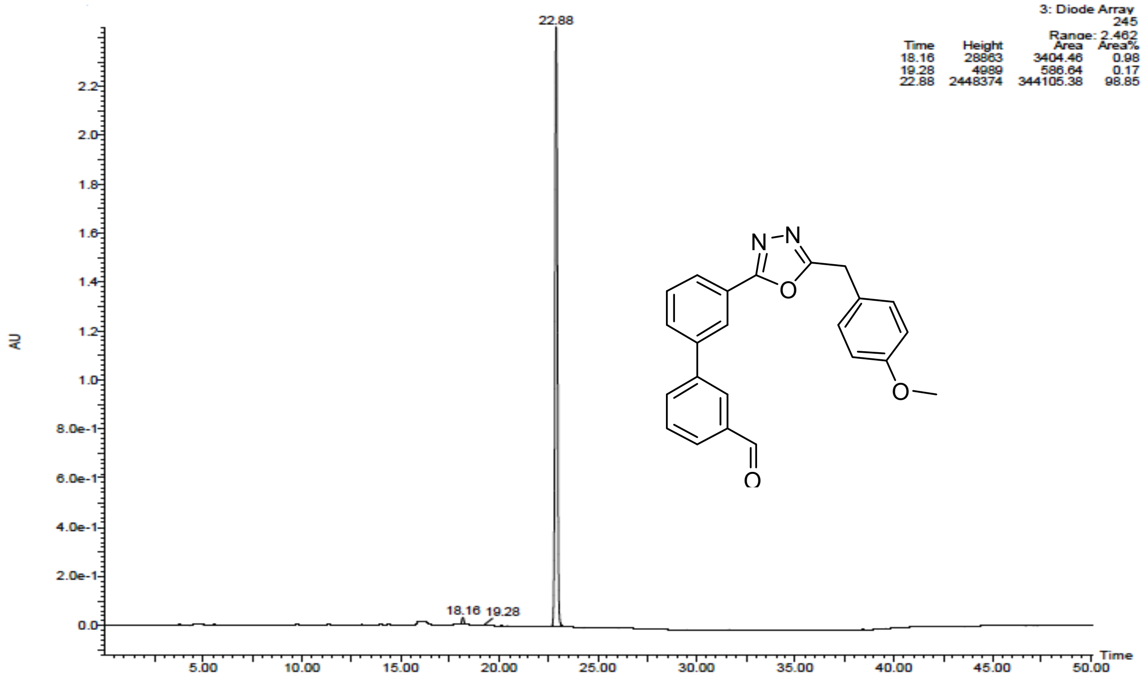

HPLC of 5u

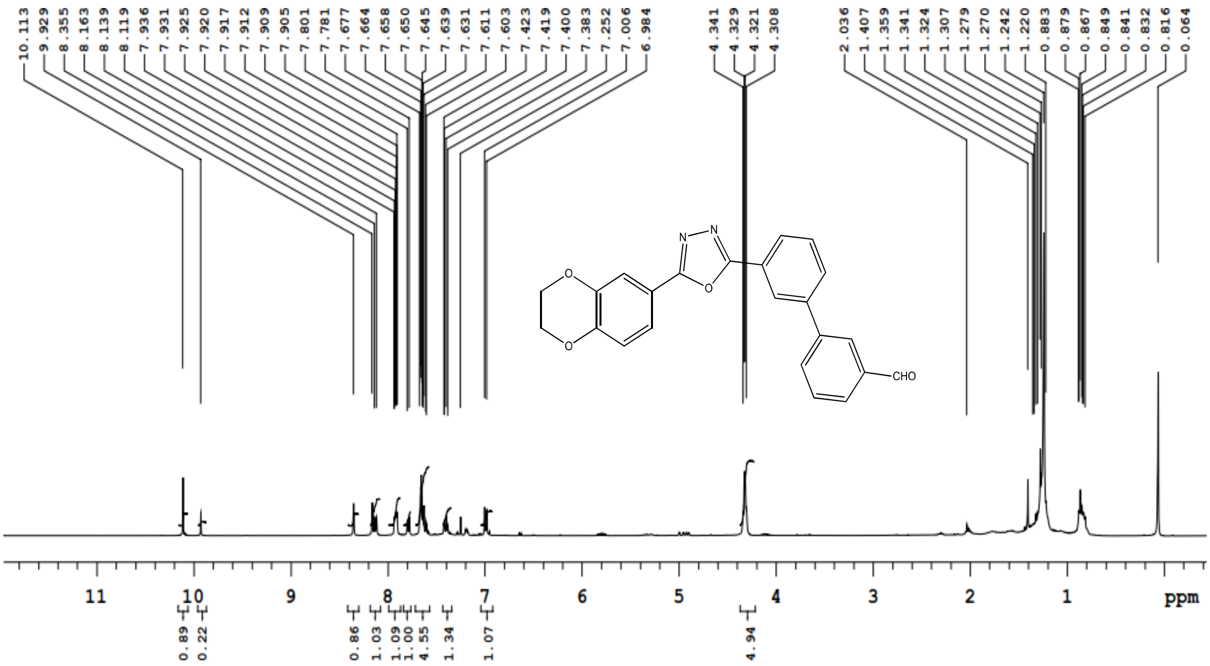

^{1}H NMR spectrum of 5v

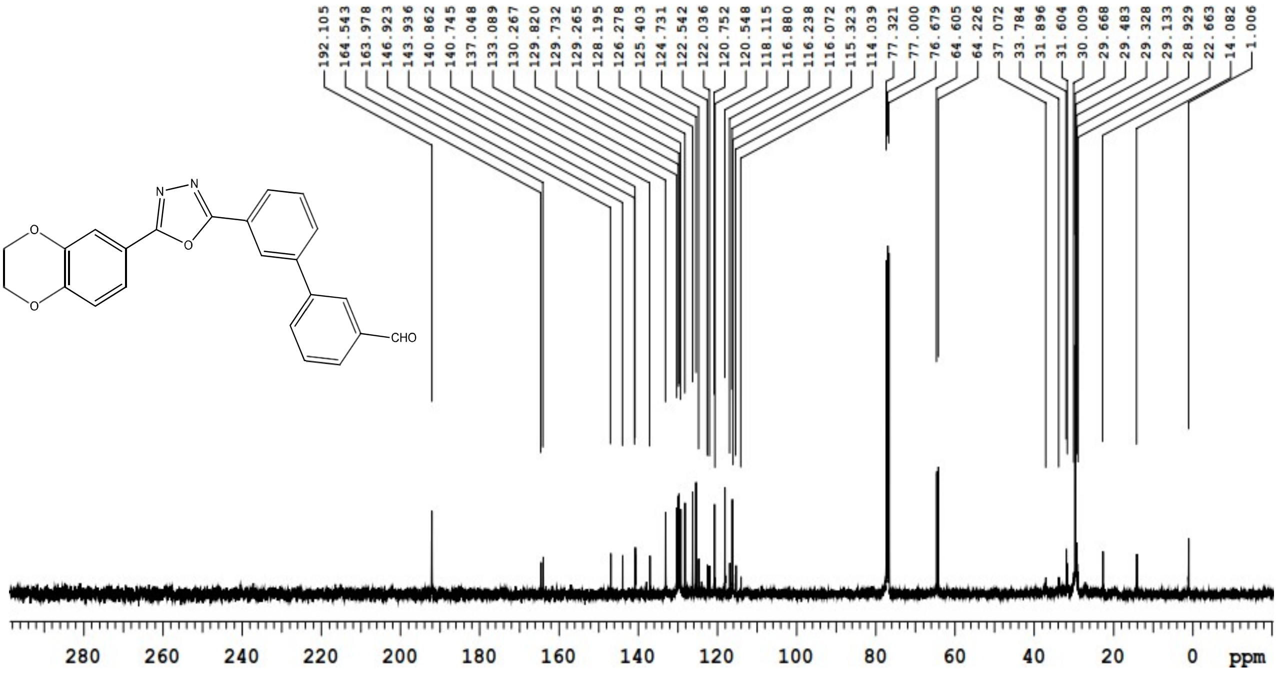

^{13}C NMR spectrum of 5v

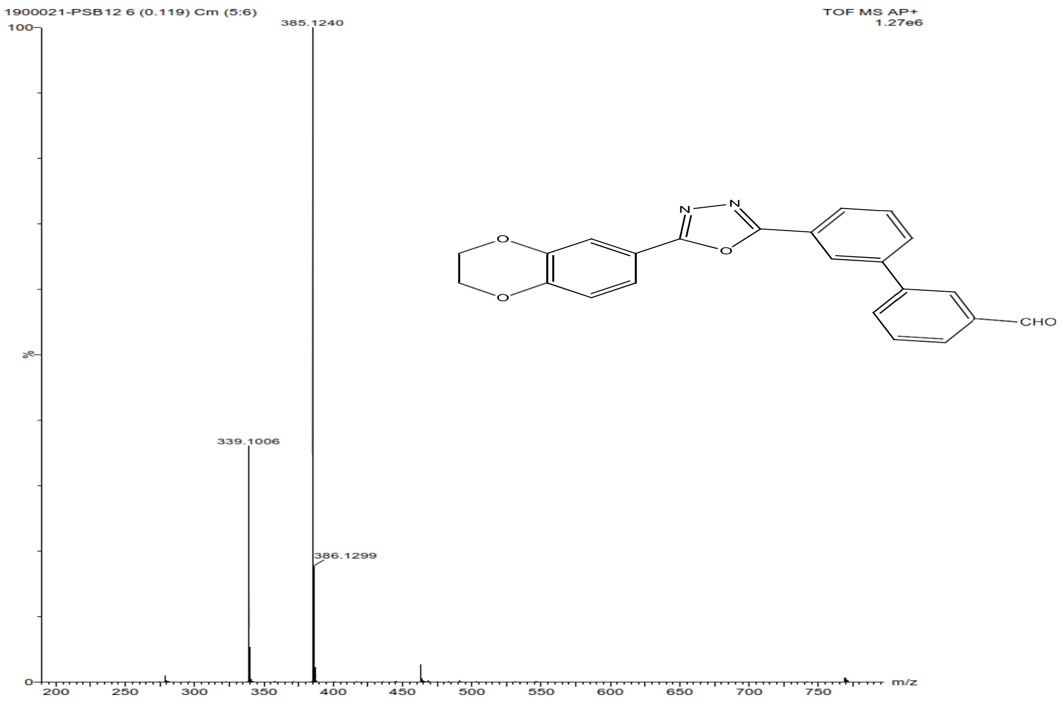

Mass spectrum of 5v

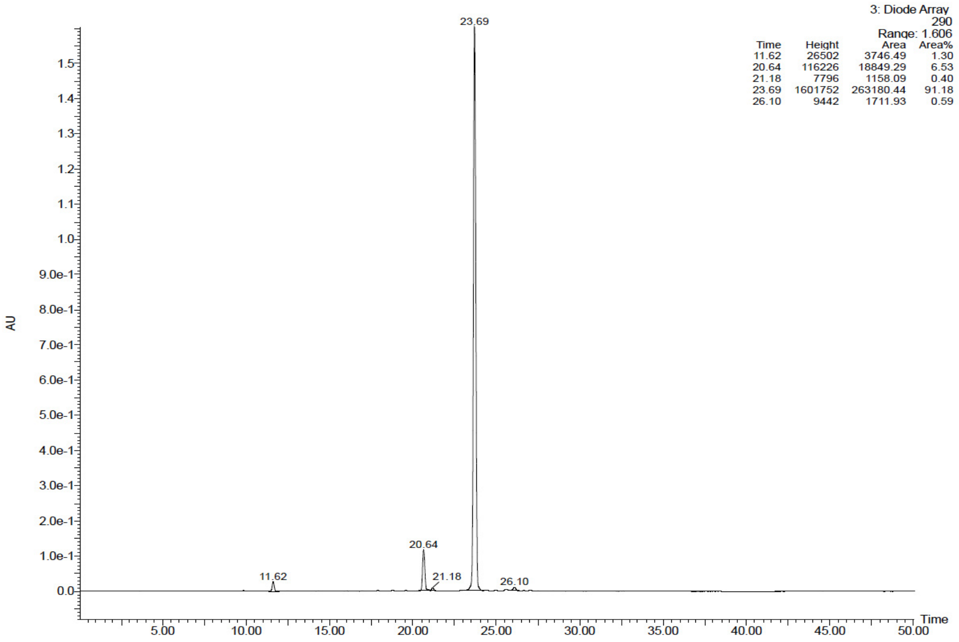

HPLC of 5v

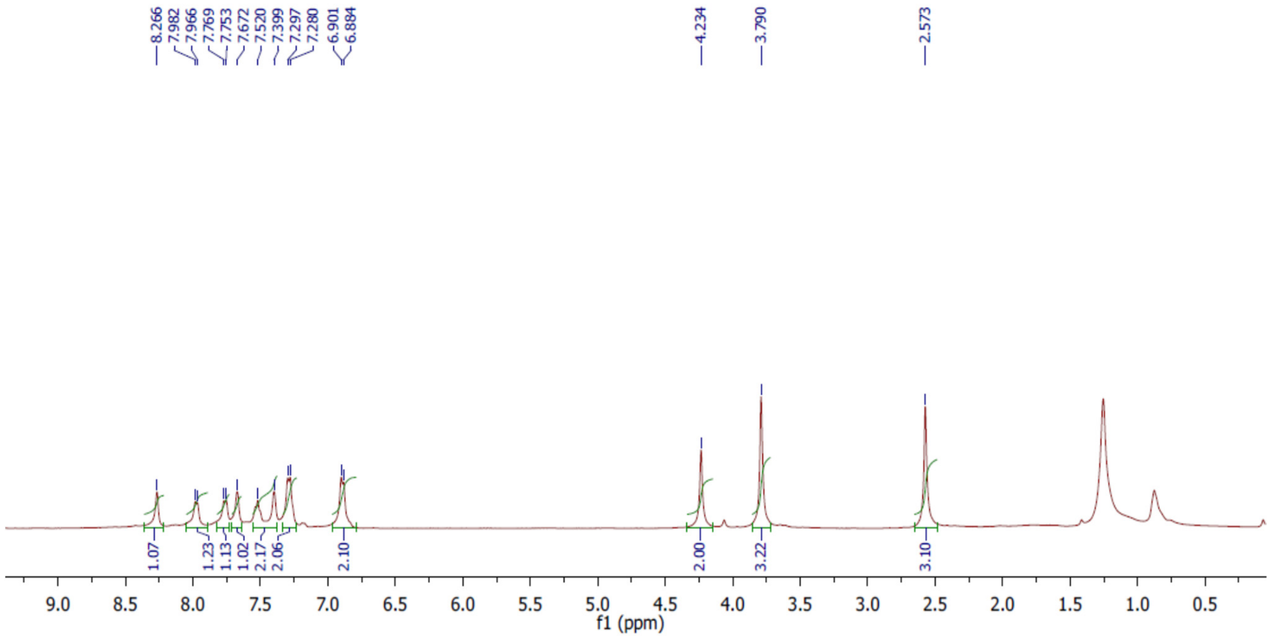

^{1}H NMR spectrum of 5w

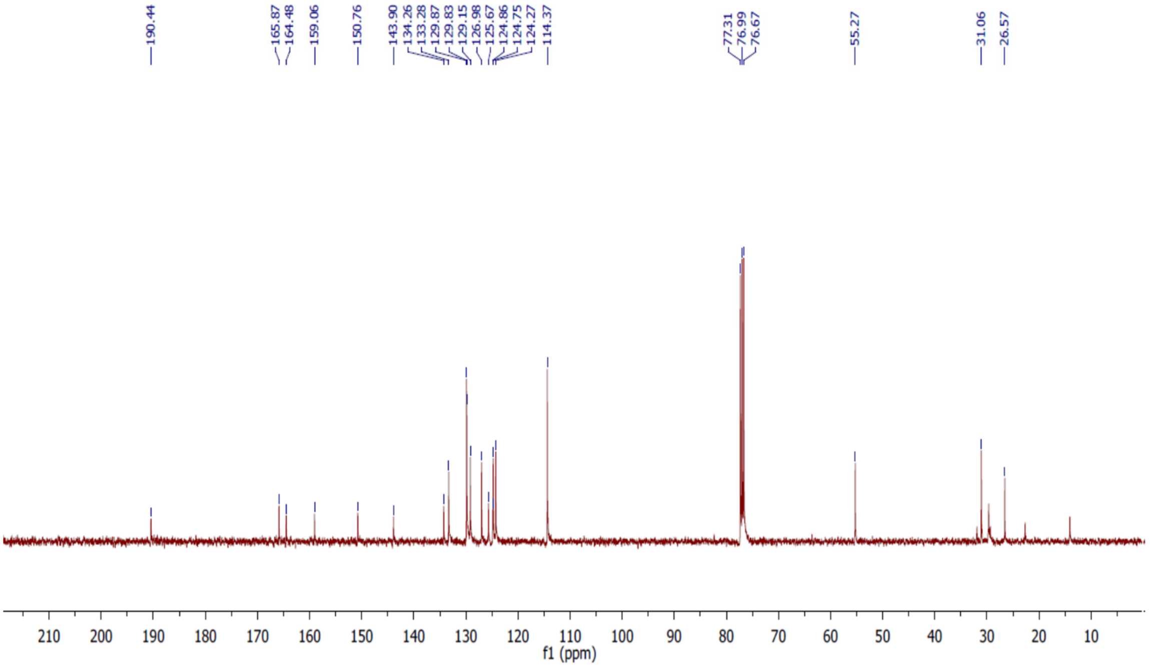

^{13}C NMR spectrum of 5w

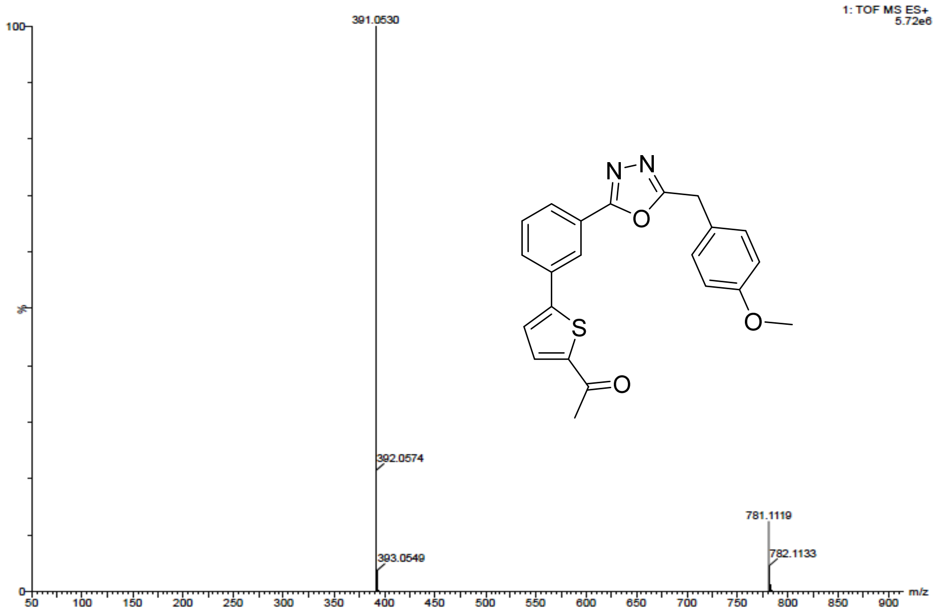

Mass spectrum of 5w

- Cell line: MCF7 (2000 cells/per well[96])
- Treated time: 72hrs
- Assay: MTT (90mins incubated)
- Data: 5w

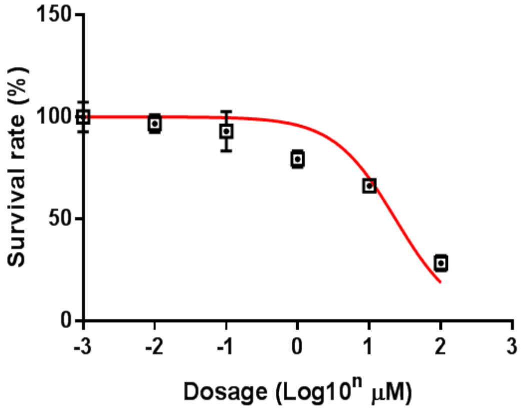

IC_{50} (μM) = 23.14
Log IC_{50} (μM) = 1.36

Conc. (μM)	Viability	
	AVE.	± SD.
**0**	100.00	7.25
**0.01**	96.74	4.40
**0.1**	92.88	9.64
**1**	79.33	4.04
**10**	66.15	2.62
**100**	28.37	3.72

Log curve for the compound 5w

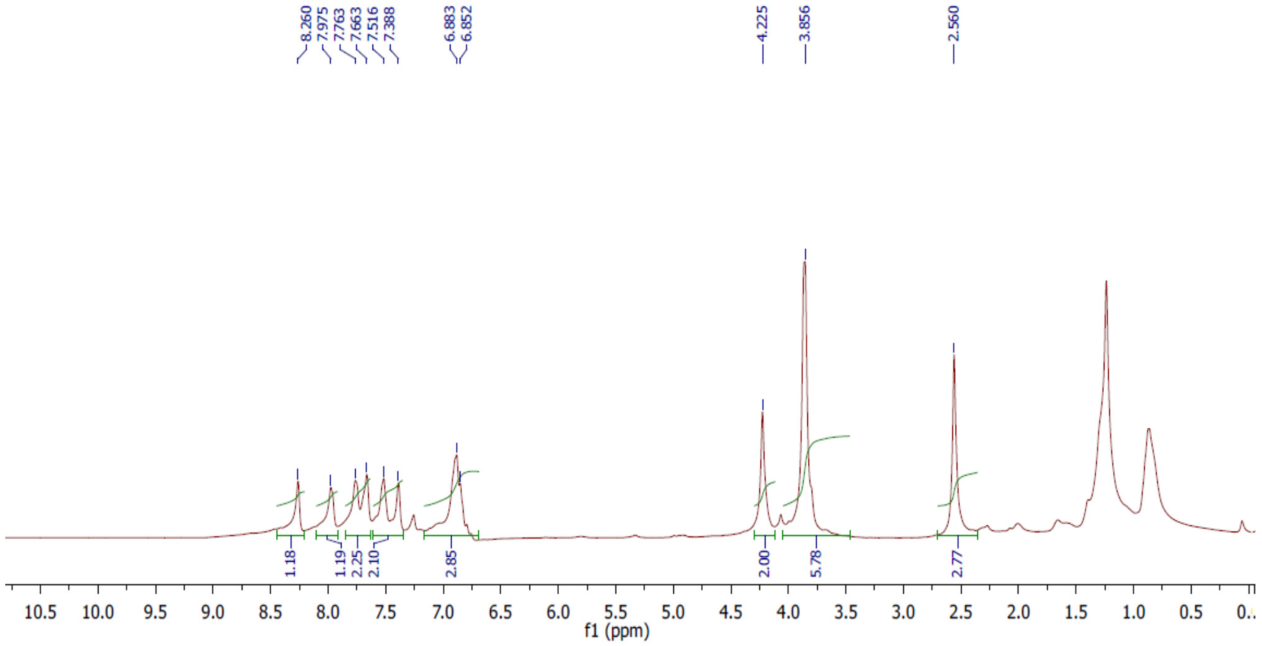

¹H NMR spectrum of 5x

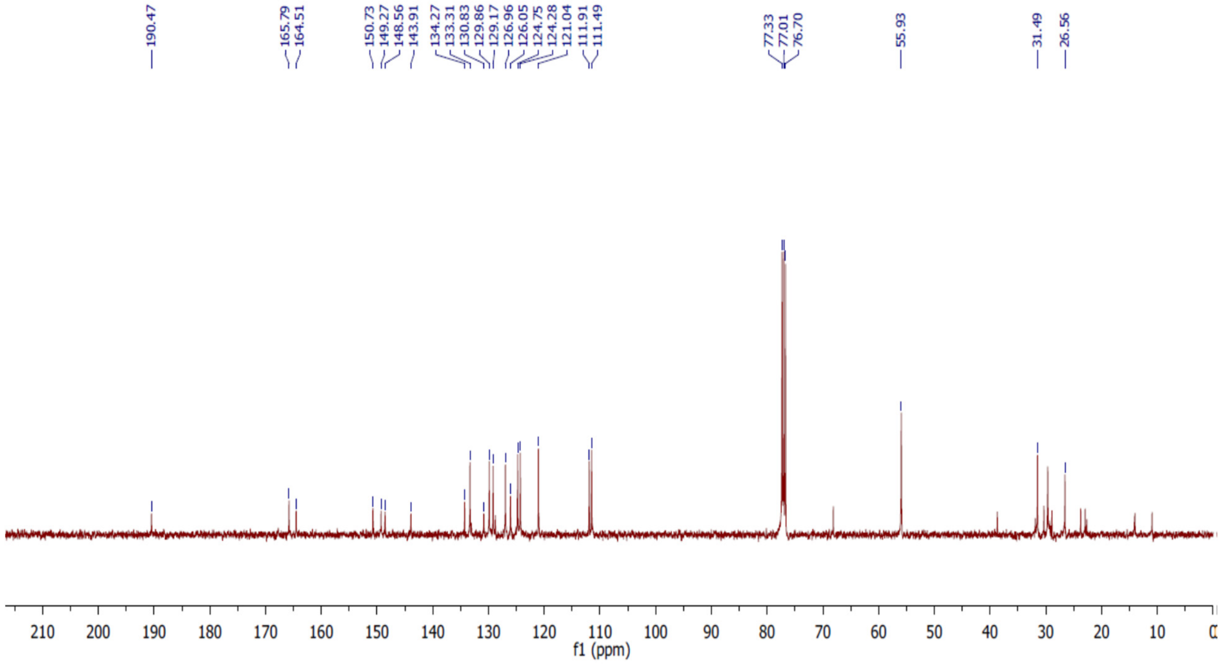

¹³C NMR spectrum of 5x

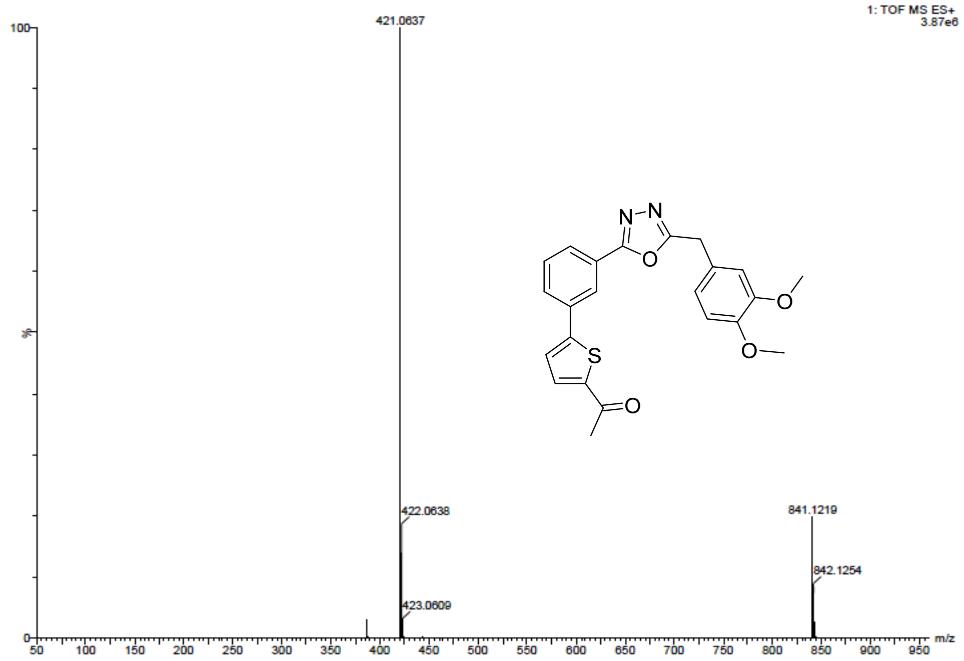

Mass spectrum of 5x

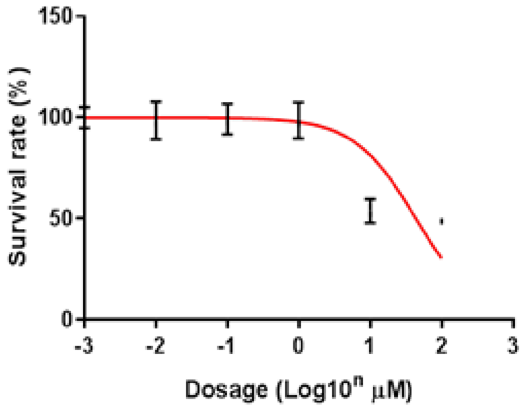

IC$_{50}$ (μM) = -
Log IC$_{50}$ (μM) = -

- Cell line: MCF7 (2000 cells/per well[96])
- Treated time: 72hrs
- Assay: MTT (90mins incubated)
- Data: 5x

Conc. (μM)	Viability	
	AVE.	± SD.
0	100.00	5.17
0.01	98.53	9.30
0.1	99.23	7.53
1	98.52	8.89
10	53.76	5.88
100	50.21	2.24

Log curve for the compound 5x
